# Supplementary material for: Mechanistic insights into photochemical nickel-catalyzed cross-couplings enabled by energy transfer
Source: Nat Commun. 2022 May 18;13:2737. doi: 10.1038/s41467-022-30278-8 (PMC9117274; doi:10.1038/s41467-022-30278-8)
Supplement: Supplementary file 1 — Supplementary Information [file 41467_2022_30278_MOESM1_ESM.pdf]

Supplementary Information

Mechanistic insights into photochemical nickel-catalyzed cross-couplings enabled by energy transfer

Rajesh Kancherla*[1,4], Krishnamoorthy Muralirajan[1,4], Bholanath Maity[1,4], Safakath Karuthedath[2], Gadde Sathish Kumar[1], Frédéric Laquai[2]*, Luigi Cavallo[1]* & Magnus Rueping[1,3]*

[1]KAUST Catalysis Center (KCC), Physical Science and Engineering Division, King Abdullah University of Science and Technology (KAUST), Thuwal 23955-6900, Kingdom of Saudi Arabia.
[2]KAUST Solar Center (KSC), Physical Science and Engineering Division, King Abdullah University of Science and Technology (KAUST), Thuwal 23955-6900, Kingdom of Saudi Arabia.
[3]RWTH Aachen University, Institute for Molecular Imaging, Forckenbeckstrasse 55, 52074.
[4]These authors contributed equally: Rajesh Kancherla, Krishnamoorthy Muralirajan, Bholanath Maity.
*email: magnus.rueping@kaust.edu.sa

Table of contents

Supplementary Methods **S3**

1. General information ………………………………………………………………... S3
2. Optimization studies ……………………………………………………………….. S4
3. General procedure for the synthesis of compounds 1 to 28 …………………….. S6
4. Preparation of Ni(dtbbpy)alkyl bromide complex mixture ……………………... S19
5. Preparation of Ni(dOMebpy)alkyl bromide complex mixture ………………….. S20
6. Steady-state Stern-Volmer quenching experiments …………………………...... S21
7. Time-resolved Stern-Volmer quenching experiments ………………………….. S22
8. Stoichiometric reaction of (dtbbpy)Ni-alkyl bromide complex with THF ……... S24
9. Kinetic isotope effect experiments ……………………………………………… S25
10. Catalytic reaction using Mischler's ketone ……………………………………... S27
11. Dependence of the reaction rate on light intensity ……………………………… S27
12. Time-resolved absorption studies ………………………………………………. S28
13. Picosecond and nanosecond-TA spectroscopy using Ni(dtbbpy)(*o*-tolyl)Cl …… S32
14. Computational methods ………………………………………………………… S34

Supplementary Discussion **S38**

1. Dexter triplet-triplet energy transfer (TTET) …………………………………… S38
2. Energy profile for the electron transfer pathway of reductive elimination step … S40
3. Alternative mechanism involving NiI/III intermediate …………………………. S40
4. PES and free energy of C_{α}−H bond activation of THF by active Br atom ……… S41
5. Discussion regarding the triplet states of nickel ………………………………… S41
6. NMR spectra …………………………………………………………………….. S43

Supplementary References **S73**

Supplementary Methods

1. General information

Reagent Information. Unless otherwise stated, all reactions were carried out under argon atmosphere in screw cap reaction tubes. All the reagents and solvents were bought from Sigma Aldrich and Alfa Aesar in a sure-seal bottle and were used as received. Ir[dF(CF_3)ppy]$_2$(dtbbpy)PF_6 was prepared by the procedure available in the literature[1] and the other photocatalysts were obtained from Sigma Aldrich and Strem chemicals. For column chromatography, silica gel (100–200 mesh) from Aldrich was used. A gradient elution using DCM/hexane and Et_2O/pentane was performed based on Merck aluminium TLC sheets (silica gel 60 F_{254}). Organic solutions were concentrated under reduced pressure on a Büchi rotary evaporator.

Analytical Information. All isolated compounds are characterized by ^{1}H NMR, ^{13}C NMR spectroscopy and gas chromatography-mass spectra (GC-MS), and high-resolution mass spectra (HRMS). Copies of the ^{1}H NMR, ^{13}C NMR can be found in the supporting information. ^{1}H NMR spectra were recorded in deuterated solvents either on a Bruker Avance-II 500 (500 and 126 MHz) or 400 (400 and 101 MHz) instrument and are internally referenced to residual protic solvent signals. The ^{1}H NMR spectra are reported as δ/ppm downfield from tetramethylsilane (multiplicity, number of protons, assignment, coupling constant *J*/Hz). All ^{1}H NMR experiments are reported as follows unless otherwise stated: chemical shift (δ ppm), integration, multiplicity (s = singlet, d = doublet, t = triplet, q = quartet, m = multiplet, dd = doublet of doublets and dt = doublet of triplets, respectively), and coupling constants (Hz). ^{13}C NMR spectra were recorded in deuterated solvents on Bruker Avance-II spectrometers at 101 or 126 MHz, with the central peak of the deuterated solvent as the internal standard. Chemical shifts (δ) are given in parts per million (ppm), and coupling constants (*J*) are given in Hertz (Hz) rounded to the nearest 0.1 Hz. The ^{13}C NMR spectra are reported as δ/ppm and were obtained with ^{1}H decoupling (note: $CDCl_3$ referenced at δ 7.26 ppm for ^{1}H NMR, 77.16 ppm for ^{13}C NMR). Assignments are aided by the use of DEPT-135 spectra where necessary. All GCMS analysis was done by Agilent 7890A GC system connected with 5975C inert XL EI/CI MSD (with triple-axis detector). High-resolution mass spectra (HRMS) analysis was performed using a Thermo LTQ Velos Orbitrap mass spectrometer (Thermo Scientific, Pittsburgh, PA, USA) equipped with an atmospheric pressure chemical ionization (APCI) source. The mass scan range was set to 100–2000 *m/z,* with a resolving power of 100,000. Luminescence intensities were recorded using a fluoromax-4 spectrophotometer from Horiba Scientific. Linear absorption spectra were collected on an Agilent 8453 Spectrophotometer.

Transient absorption (TA) spectroscopy was carried out using a home-built pump-probe setup. The output of a titanium:sapphire amplifier (Coherent LEGEND DUO, 4.5 mJ, 3 kHz, 100 fs) was split

into three beams (2 mJ, 1 mJ, and 1.5 mJ). Two of them were used to separately pump two optical parametric amplifiers (OPA) (Light Conversion TOPAS Prime). The TOPAS 1 generates tunable pump pulses, while the TOPAS 2 generates signal (1300 nm) and idler (2000 nm) only. TOPAS 2 was used to produce a white-light supercontinuum from 350 to 1100 nm by sending the 1300 nm pulses through a calcium fluoride (CaF2) crystal which is mounted on a continuously moving stage. The excitation light (pump pulse) was provided by an actively Q-switched Nd:YVO4 laser (InnoLas picolo AOT) frequency-doubled to provide pulses at 355 nm. The pump laser was triggered by an electronic delay generator (Stanford Research Systems DG535) itself triggered by the transistor-transistor logic (TTL) sync from the Legend DUO, allowing control of the delay between pump and probe with a jitter of roughly 100 ps. Pump and probe beams were focused on the sample. The transmitted fraction of the white light was guided to a custom-made prism spectrograph (Entwicklungsbüro Stresing) where it was dispersed by a prism onto a 512-pixel complementary metal-oxide-semiconductor (CMOS) linear image sensor (Hamamatsu G11608-512DA). The probe pulse repetition rate was 3 kHz and the excitation pulses were directly generated at 1.5 kHz frequency, while the detector array was read out at 3 kHz. Adjacent diode readings corresponding to the transmission of the sample after excitation and in the absence of an excitation pulse were used to calculate $\Delta T/T$ (or DAS). Measurements were averaged over several thousand shots to obtain a good signal-to-noise ratio. The delay at which pump and probe arrive simultaneously on the sample (i.e., zero time) was determined from the point of the maximum positive slope of the TA signal rise for each wavelength.

All reaction mixtures were irradiated with 34 W Kessil KSH150B from 4 cm away. The emission maximum of the light source used is 425 nm. Regular fans are employed to maintain the temperature at room temperature.

2. Optimization studies

Procedure for reaction optimization: An oven-dried screw cap reaction tube and a 5 mL vial equipped with a PTFE-coated stir bar were brought into the N_2-filled glove box. Ni(cod)$_2$ (2 mol%), 4,4′-dimethoxy-2,2′-bipyridyl (4,4′-dOMe-bpy, 2.2 mol%) and THF (1.5 mL) was added in to 5 mL vial and stirred well for 15 min to give a deep purple color solution (Mixture 1). The other reaction tube was charged with Ir[dF(CF$_3$)ppy]$_2$(dtbbpy)PF$_6$ (PS1, 2 mol%), K_2CO_3 (2 equiv.), THF (0.5 mL), (3-bromopropyl)benzene (0.1 mmol, 1 equiv.) and stirred. Mixture 1 was then added to the reaction tube, capped with Teflon septum and parafilmed. The reaction tube was removed from the glove box and irradiated using 34 W blue LEDs while stirring at RT (under fan cooling to keep the reaction at room temperature). After 48 hours, the reaction mixture was diluted with EtOAc then trimethoxy benzene was added as the internal standard. Yields were determined by the ^{1}H NMR by the integration method and by the Gas Chromatography.

Supplementary Table 1. Optimization of the reaction conditions[a]

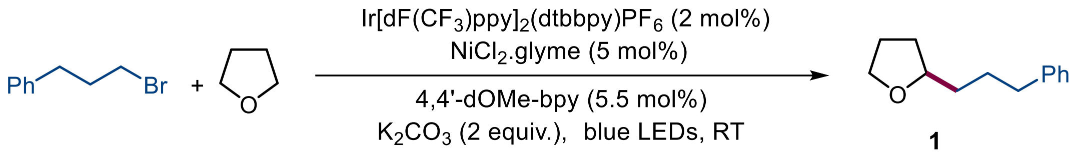

Entry	Change in the standard reaction conditions	Yield (**1**, %)[b]
1	$NiCl_2$.glyme (5 mol%), 4,4'-dtbbpy (6 mol%)	36
2	None	54
3	$Ni(cod)_2$ (5 mol%), 4,4'-dOMe-bpy (5.5 mol%)	77
4	$Ni(cod)_2$ (10 mol%), 4,4'-dOMe-bpy (11 mol%)	75
5	H_2O (10 equiv.) as additive	73
6	1 mol% $Ir[dF(CF_3)ppy]_2(dtbbpy)PF_6$, $Ni(cod)_2$ (5 mol%)	74
7	$Ir[ppy]_2(dtbbpy)PF_6$ instead of PS1	0
8	$Ru(bpz)_3{\cdot}2PF_6$ instead of PS1	0
9	(9-MesAcr)ClO_4 instead of PS1	0
10	4-CzIPN (2 mol%) instead of PS1, $Ni(cod)_2$ (5 mol%)	30
11	without [Ir] photosensitizer	0
12	without [Ni] catalyst	0
13	without Light source	0
14	without base	trace
15	without fan cooling (around 50 °C)	51
16	(3-chloropropyl)benzene instead of alkyl bromide	0

[a]Alkyl bromide (0.1 mmol), THF (0.05 M, 2 mL), $Ir[dF(CF_3)ppy]_2(dtbbpy)PF_6$ (2 mol%), $NiCl_2$.glyme (5 mol%), 4,4'-dOMe-bpy (4,4'-dimethoxy-2,2′-bipyridyl) (5.5 mol%), K_2CO_3 (2 equiv.), 34 W blue LEDs, Ar, 48 h, room temperature. [b] Yield determined by GC.

Supplementary Table 2. Optimization by varying the concentration of THF

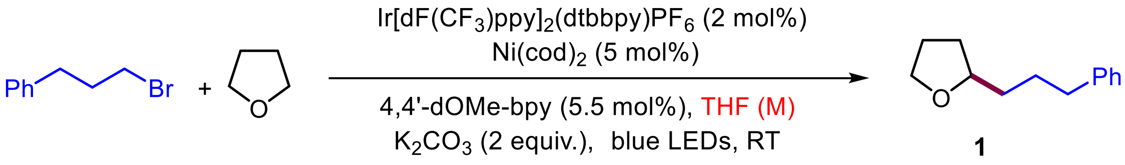

Entry	THF as solvent	Yield (**1**, %)
1	0.1 M	48
2	0.066 M	67
3	0.05 M	77
4	0.04 M	73

Supplementary Table 3. Optimization by varying the base

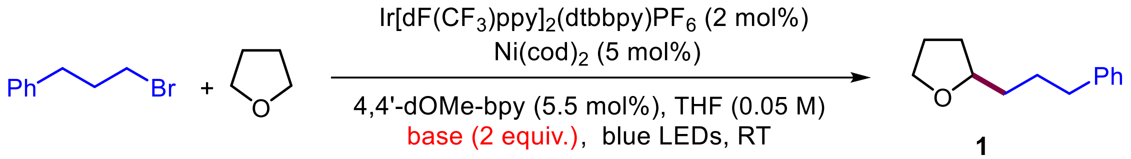

1

Entry	base (2 equiv)	Yield (**1**, %)
1	K_3PO_4	39
2	Cs_2CO_3	74
3	KOH	trace
4	K_2CO_3	77
5	KO*t*Bu	trace
6	$NaHCO_3$	39
7	Na_2CO_3	71

3. General procedure for the synthesis of compounds 1 to 28

An oven-dried screw cap reaction tube and a 5 mL vial equipped with a PTFE-coated stir bar were brought into the N_2-filled glove box. $Ni(cod)_2$ (2.75 mg, 0.01 mmol, 5 mol%), 4,4′-dimethoxy-2,2′-bipyridyl (2.4 mg, 0.011 mmol, 5.5 mol%) and C(sp^3)–H coupling partner (3 mL) was added in to 5 mL vial and stirred well for 15 min to give a deep purple color solution (Mixture 1). The other reaction tube was charged with $Ir[dF(CF_3)ppy]_2(dtbbpy)PF_6$ (4.5 mg, 0.004 mmol, 2 mol%), K_2CO_3 (55 mg, 0.4 mmol, 2 equiv.), C(sp^3)–H coupling partner (1 mL), alkyl bromides (0.2 mmol, 1 equiv.) and stirred. Mixture 1 was then added to the reaction tube, capped with Teflon septum and parafilmed. The reaction tube was removed from the glove box and irradiated using 34 W blue LEDs while stirring at RT (under fan cooling to keep the reaction at room temperature). After 48 hours, the reaction was filtered through a small bed of celite and silica and concentrated *in vacuo*. The residue was purified by column chromatography using silica gel (100-200 mesh size) and DCM/hexane or Et_2O/pentane as the eluent. In the case of 1,4-dioxane and toluene coupling partners, two 34 W blue LEDs were used to irradiate for 96 hours.

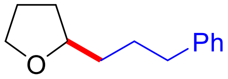

2-(3-Phenylpropyl)tetrahydrofuran (1): Prepared following the general procedure outlined above using $Ir[dF(CF_3)ppy]_2(dtbbpy)PF_6$ (4.5 mg, 0.004 mmol, 2 mol%), $Ni(cod)_2$ (2.75 mg, 0.01 mmol, 5 mol%), 4,4′-dOMe-bpy (2.4 mg, 0.011 mmol, 5.5 mol%), (3-bromopropyl)benzene (30.4 μL, 0.2 mmol), K_2CO_3 (55 mg, 0.4 mmol, 2 equiv.) and THF (4 mL). Pure product was obtained

as clear oil in 75% (28.5 mg) yield after column chromatography of the crude reaction mixture (silica gel, gradient 0 to 5% Et_2O/pentane).

^{1}H NMR (500 MHz, $CDCl_3$) δ 7.28 (t, J = 7.6 Hz, 2H), 7.16 – 7.19 (m, 3H), 3.88 – 3.78 (m, 2H), 3.73 – 3.69 (m, 1H), 2.69 – 2.60 (m, 2H), 1.99 – 1.93 (m, 1H), 1.91 – 1.71 (m, 3H), 1.67 – 1.59 (m, 2H), 1.54 – 1.39 (m, 2H). ^{13}C NMR (126 MHz, $CDCl_3$) δ 142.58, 128.55, 128.37, 125.78, 79.36, 77.41, 76.91, 67.77, 36.09, 35.43, 31.48, 28.32, 25.85. HRMS ESI (m/z): $(M-H)^+$ calcd. for $C_{13}H_{17}O$, 189.1273; found, 189.1269. GC-MS (m/z): 190.1 $[M]^+$, 172.1, 144.1, 131.1, 104.0, 91.0, 71.0, 43.1. Data are consistent with those reported in the literature.[2]

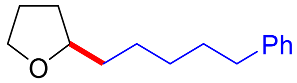

2-(5-Phenylpentyl)tetrahydrofuran (2): Prepared following the general procedure outlined above using $Ir[dF(CF_3)ppy]_2(dtbbpy)PF_6$ (4.5 mg, 0.004 mmol, 2 mol%), $Ni(cod)_2$ (2.75 mg, 0.01 mmol, 5 mol%), 4,4′-dOMe-bpy (2.4 mg, 0.011 mmol, 5.5 mol%), (5-bromopentyl)benzene (45.4 µL, 0.2 mmol), K_2CO_3 (55 mg, 0.4 mmol, 2 equiv.) and THF (4 mL). Pure product was obtained as clear oil in 77% (33.5 mg) yield after column chromatography of the crude reaction mixture (silica gel, gradient 0 to 5% Et_2O/pentane).

^{1}H NMR (400 MHz, $CDCl_3$) δ 7.28 (t, J = 7.6 Hz, 2H), 7.19 – 7.16 (m, 3H), 3.89 – 3.84 (m, 1H), 3.81 – 3.75 (m, 1H), 3.74 – 3.69 (m, 1H), 2.62 (t, J = 7.6 Hz, 2H), 2.01 – 1.81 (m, 3H), 1.68 – 1.55 (m, 3H), 1.50 – 1.35 (m, 6H). ^{13}C NMR (101 MHz, $CDCl_3$) δ 142.91, 128.50, 128.33, 125.67, 79.50, 67.72, 36.02, 35.79, 31.59, 31.51, 29.52, 26.39, 25.84. HRMS ESI (m/z): $(M+H)^+$ calcd. for $C_{15}H_{23}O$, 219.1743; found, 219.1739. GC-MS (m/z): 218.1 $[M]^+$, 200.1, 158.1, 144.1, 129.1, 117.0, 104.0, 91.0, 71.0, 55.0, 43.0.

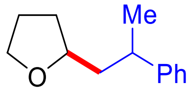

2-(2-Phenylpropyl)tetrahydrofuran (3): Prepared following the general procedure outlined above using $Ir[dF(CF_3)ppy]_2(dtbbpy)PF_6$ (4.5 mg, 0.004 mmol, 2 mol%), $Ni(cod)_2$ (2.75 mg, 0.01 mmol, 5 mol%), 4,4′-dOMe-bpy (2.4 mg, 0.011 mmol, 5.5 mol%), (1-bromopropan-2-yl)benzene (40 mg, 0.2 mmol), K_2CO_3 (55 mg, 0.4 mmol, 2 equiv.) and THF (4 mL). Pure product was obtained as clear oil in 49% (18.6 mg) yield after column chromatography of the crude reaction mixture (silica gel, gradient 0 to 5% Et_2O/pentane).

^{1}H NMR (500 MHz, $CDCl_3$) δ 7.32 – 7.28 (m, 2H), 7.24 – 7.18 (m, 3H), 3.88 – 3.83 (m, 1H), 3.74 – 3.55 (m, 2H), 2.97 – 2.82 (m, 1H), 2.01 – 1.62 (m, 5H), 1.49 – 1.24 (m, 4H). ^{13}C NMR (126 MHz, $CDCl_3$) δ 147.52, 147.16, 128.51, 128.44, 127.31, 126.95, 126.06, 77.52, 77.21, 67.61, 44.64, 44.16, 37.61, 37.10, 31.86, 31.50, 25.78, 23.20, 22.33. HRMS ESI (m/z): $(M-H)^+$ calcd. for

$C_{13}H_{19}O$, 191.1430; found, 191.1426. GC-MS (*m/z*): 190.1 $[M]^+$, 143, 131, 118, 105, 91, 85, 77, 71, 51, 43.

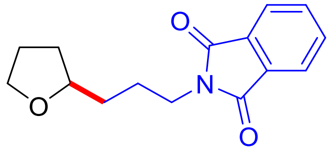

2-(3-(Tetrahydrofuran-2-yl)propyl)isoindoline-1,3-dione (4): Prepared following the general procedure outlined above using Ir[dF(CF_3)ppy]$_2$(dtbbpy)PF_6 (4.5 mg, 0.004 mmol, 2 mol%), Ni(cod)$_2$ (2.75 mg, 0.01 mmol, 5 mol%), 4,4′-dOMe-bpy (2.4 mg, 0.011 mmol, 5.5 mol%), *N*-(3-bromopropyl)phthalimide (53.6 mg, 0.2 mmol), K_2CO_3 (55 mg, 0.4 mmol, 2 equiv.) and THF (4 mL). Pure product was obtained as clear oil in 63% (32.5 mg) yield after column chromatography of the crude reaction mixture (silica gel, gradient 5 to 15% DCM/hexane).

^{1}H NMR (400 MHz, $CDCl_3$) δ 7.82 (dd, *J* = 5.4, 3.1 Hz, 2H), 7.69 (dd, *J* = 5.4, 3.0 Hz, 2H), 3.84 – 3.77 (m, 2H), 3.74 – 3.65 (m, 3H), 1.99 – 1.91 (m, 1H), 1.89 – 1.38 (m, 7H). ^{13}C NMR (101 MHz, $CDCl_3$) δ 168.53, 133.97, 132.26, 123.28, 78.81, 77.48, 76.84, 67.80, 38.09, 32.93, 31.43, 25.83, 25.63. HRMS ESI (m/z): $(M+H)^+$ calcd. for $C_{15}H_{18}O_3N$, 260.1281; found, 260.1275. GC-MS (*m/z*): 259.1 $[M]^+$, 231.1, 216.1, 173.0, 160.0, 130.0, 110.0, 84.0, 71.0, 43.1. Data are consistent with those reported in the literature.[3]

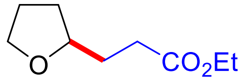

Ethyl 3-(tetrahydrofuran-2-yl)propanoate (5): Prepared following the general procedure outlined above using Ir[dF(CF_3)ppy]$_2$(dtbbpy)PF_6 (4.5 mg, 0.004 mmol, 2 mol%), Ni(cod)$_2$ (2.75 mg, 0.01 mmol, 5 mol%), 4,4′-dOMe-bpy (2.4 mg, 0.011 mmol, 5.5 mol%), ethyl 3-bromopropanoate (25.6 μL, 0.2 mmol), K_2CO_3 (55 mg, 0.4 mmol, 2 equiv.) and THF (4 mL). Pure product was obtained as clear oil in 43% (14.8 mg) yield after column chromatography of the crude reaction mixture (silica gel, gradient 0 to 20% DCM/hexane).

^{1}H NMR (500 MHz, $CDCl_3$) δ 4.12 (q, *J* = 5 Hz, 2H), 3.85 – 3.80 (m, 2H), 3.71 (td, *J* = 8.0, 6.2 Hz, 1H), 2.47 – 2.29 (m, 2H), 2.01 – 1.95 (m, 1H), 1.91 – 1.62 (m, 4H), 1.50 – 1.43 (m, 1H), 1.24 (t, *J* = 7.1 Hz, 3H). ^{13}C NMR (126 MHz, $CDCl_3$) δ 173.74, 173.50, 78.29, 67.81, 60.43, 34.06, 31.29, 30.83, 25.82, 24.50, 14.34. HRMS ESI (m/z): $(M+H)^+$ calcd. for $C_9H_{17}O_3$, 173.1172; found, 173.1167. GC-MS (*m/z*): 172.1 $[M]^+$, 171.1, 144.1, 127.1, 98.0, 85.0, 71.0, 55.0, 43.0.

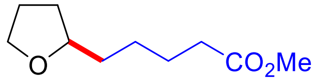

Methyl 5-(tetrahydrofuran-2-yl)pentanoate (6): Prepared following the general procedure outlined above using Ir[dF(CF_3)ppy]$_2$(dtbbpy)PF_6 (4.5 mg, 0.004 mmol, 2 mol%), Ni(cod)$_2$ (2.75

mg, 0.01 mmol, 5 mol%), 4,4′-dOMe-bpy (2.4 mg, 0.011 mmol, 5.5 mol%), methyl 5-bromopentanoate (29 µL, 0.2 mmol), K_2CO_3 (55 mg, 0.4 mmol, 2 equiv.) and THF (4 mL). Pure product was obtained as clear oil in 61% (22.6 mg) yield after column chromatography of the crude reaction mixture (silica gel, gradient 0 to 20% DCM/hexane).

^{1}H NMR (400 MHz, $CDCl_3$) δ 3.86 – 3.66 (m, 3H), 3.65 (s, 3H), 2.30 (t, *J* = 7.5 Hz, 2H), 1.96 – 1.82 (m, 3H), 1.68 – 1.31 (m, 7H). ^{13}C NMR (101 MHz, $CDCl_3$) δ 174.26, 79.24, 67.74, 51.58, 35.44, 34.16, 31.49, 26.09, 25.82, 25.14. HRMS ESI (m/z): $(M+H)^+$ calcd. for $C_{10}H_{19}O_3$, 187.1328; found, 187.1323. GC-MS (*m/z*): 186.0 $[M]^+$, 155.1, 126.0, 71.0, 55.0, 43.0. Data are consistent with those reported in the literature.[4]

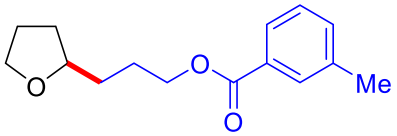

3-(Tetrahydrofuran-2-yl)propyl 3-methylbenzoate (7): Prepared following the general procedure outlined above using Ir[dF(CF_3)ppy]$_2$(dtbbpy)PF_6 (4.5 mg, 0.004 mmol, 2 mol%), Ni(cod)$_2$ (2.75 mg, 0.01 mmol, 5 mol%), 4,4′-dOMe-bpy (2.4 mg, 0.011 mmol, 5.5 mol%), 3-bromopropyl 3-methylbenzoate (51.5 mg, 0.2 mmol), K_2CO_3 (55 mg, 0.4 mmol, 2 equiv.) and THF (4 mL). Pure product was obtained as clear oil in 65% (32.2 mg) yield after column chromatography of the crude reaction mixture (silica gel, gradient 0 to 20% DCM/hexane).

^{1}H NMR (500 MHz, $CDCl_3$) δ 7.84 – 7.82 (m, 2H), 7.36 – 7.29 (m, 2H), 4.37 – 4.29 (m, 2H), 3.89 – 3.82 (m, 2H), 3.74 – 3.70 (m, 1H), 2.39 (s, 3H), 2.03 – 1.97 (m, 1H), 1.95 – 1.77 (m, 4H), 1.73 – 1.59 (m, 2H), 1.50 – 1.41 (m, 1H). ^{13}C NMR (126 MHz, $CDCl_3$) δ 166.90, 138.18, 133.70, 130.38, 130.15, 128.31, 126.77, 78.92, 67.81, 65.00, 32.22, 31.48, 25.82, 21.39. HRMS ESI (m/z): $(M+H)^+$ calcd. for $C_{15}H_{21}O_3$, 249.1485; found, 249.1480. GC-MS (*m/z*): 248.1 $[M]^+$, 220.1, 205.1, 136.0, 119.0, 91.0, 84.0, 71.0, 43.1.

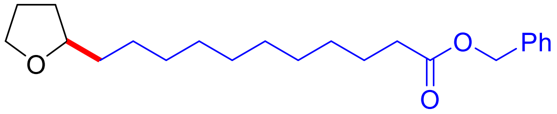

Benzyl 11-(tetrahydrofuran-2-yl)undecanoate (8): Prepared following the general procedure outlined above using Ir[dF(CF_3)ppy]$_2$(dtbbpy)PF_6 (2.25 mg, 0.002 mmol, 2 mol%), Ni(cod)$_2$ (1.37 mg, 5 µmol, 5 mol%), 4,4′-dOMe-bpy (1.2 mg, 5.5 µmol, 5.5 mol%), benzyl 11-bromoundecanoatee (36 mg, 0.1 mmol), K_2CO_3 (27.5 mg, 0.2 mmol, 2 equiv.) and THF (2 mL). Pure product was obtained as clear oil in 60% (20.7 mg) yield after column chromatography of the crude reaction mixture (silica gel, gradient 0 to 15% DCM/hexane).

^{1}H NMR (400 MHz, $CDCl_3$) δ 7.38 – 7.29 (m, 5H), 5.11 (s, 2H), 3.88 – 3.83 (m, 1H), 3.80 – 3.67 (m, 2H), 2.35 (t, *J* = 7.5 Hz, 2H), 1.99 – 1.80 (m, 3H), 1.68 – 1.60 (m, 3H), 1.47 – 1.39 (m, 3H),

1.31 – 1.25 (m, 13H). ^{13}C NMR (101 MHz, $CDCl_3$) δ 173.84, 136.27, 128.66, 128.29, 79.60, 67.73, 66.19, 35.89, 34.48, 31.52, 29.87, 29.71, 29.64, 29.56, 29.37, 29.26, 26.55, 25.86, 25.10. HRMS ESI (m/z): $(M+H)^+$ calcd. for $C_{22}H_{35}O_3$, 347.2580; found, 347.2576. GC-MS (*m/z*): 346.2 $[M]^+$, 345.1, 285.1, 255.2, 207.0, 108.0, 91.0, 71.0, 55.0, 43.0.

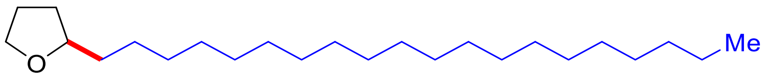

2-Icosyltetrahydrofuran (9): Prepared following the general procedure outlined above using Ir[dF(CF_3)ppy]$_2$(dtbbpy)PF_6 (4.5 mg, 0.004 mmol, 2 mol%), Ni(cod)$_2$ (2.75 mg, 0.01 mmol, 5 mol%), 4,4′-dOMe-bpy (2.4 mg, 0.011 mmol, 5.5 mol%), 1-bromoicosane (73 mg, 0.2 mmol), K_2CO_3 (55 mg, 0.4 mmol, 2 equiv.) and THF (4 mL). Pure product was obtained as solid in 67% (47 mg) yield after column chromatography of the crude reaction mixture (silica gel, gradient 0 to 3% Et_2O/pentane).

^{1}H NMR (400 MHz, $CDCl_3$) δ 3.88 – 3.82 (m, 1H), 3.80 – 3.67 (m, 2H), 1.99 – 1.83 (m, 3H), 1.61 – 1.51 (m, 1H), 1.46 – 1.37 (m, 3H), 1.25 (s, 35H), 0.87 (t, J = 6.7 Hz, 3H). ^{13}C NMR (101 MHz, $CDCl_3$) δ 79.63, 67.73, 35.91, 32.09, 31.53, 29.92, 29.79, 29.52, 26.57, 25.87, 22.85, 14.27. HRMS ESI (m/z): $(M+H)^+$ calcd. for $C_{24}H_{49}O$, 353.3777; found, 353.3769. GC-MS (*m/z*): 352.4 $[M]^+$, 351.4, 334.3, 207.0, 123.1, 109.1, 97.1, 83.1, 71.0, 55.0, 43.1.

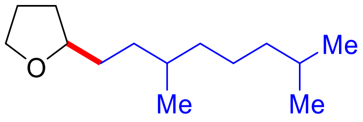

2-(3,7-Dimethyloctyl)tetrahydrofuran (10): Prepared following the general procedure outlined above using Ir[dF(CF_3)ppy]$_2$(dtbbpy)PF_6 (4.5 mg, 0.004 mmol, 2 mol%), Ni(cod)$_2$ (2.75 mg, 0.01 mmol, 5 mol%), 4,4′-dOMe-bpy (2.4 mg, 0.011 mmol, 5.5 mol%), 1-bromo-3,7-dimethyloctane (41.4 µL, 0.2 mmol), K_2CO_3 (55 mg, 0.4 mmol, 2 equiv.) and THF (4 mL). Pure product was obtained as clear oil in 52% (22 mg) yield after column chromatography of the crude reaction mixture (silica gel, gradient 0 to 3% Et_2O/pentane).

^{1}H NMR (400 MHz, $CDCl_3$) δ 3.88 – 3.83 (m, 1H), 3.77 – 3.67 (m, 2H), 2.01 – 1.81 (m, 3H), 1.65 – 1.35 (m, 6H), 1.32 – 1.18 (m, 4H), 1.15 – 1.04 (m, 3H), 0.85 (d, J = 6.6 Hz, 9H). ^{13}C NMR (101 MHz, $CDCl_3$) δ 80.01, 79.97, 67.74, 39.48, 37.36, 37.30, 33.71, 33.38, 33.36, 33.06, 33.03, 31.54, 31.52, 28.10, 25.85, 24.89, 24.87, 22.85, 22.76, 19.78. HRMS ESI (m/z): $(M+H)^+$ calcd. for $C_{14}H_{29}O$, 213.2212; found, 213.2206. GC-MS (*m/z*): 212.1 $[M]^+$, 194.2, 126.1, 109.1, 81.1, 71.0, 55.1, 43.1.

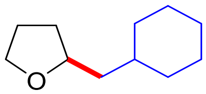

2-(Cyclohexylmethyl)tetrahydrofuran (11): Prepared following the general procedure outlined above using Ir[dF(CF_3)ppy]$_2$(dtbbpy)PF_6 (4.5 mg, 0.004 mmol, 2 mol%), Ni(cod)$_2$ (2.75 mg, 0.01 mmol, 5 mol%), 4,4′-dOMe-bpy (2.4 mg, 0.011 mmol, 5.5 mol%), (bromomethyl)cyclohexane (27.2 μL, 0.2 mmol), K_2CO_3 (55 mg, 0.4 mmol, 2 equiv.) and THF (4 mL). Pure product was obtained as clear oil in 54% (18.2 mg) yield after column chromatography of the crude reaction mixture (silica gel, gradient 0 to 5% Et_2O/pentane).

^{1}H NMR (400 MHz, $CDCl_3$) δ 3.93 – 3.81 (m, 2H), 3.72 – 3.66 (m, 1H), 2.02 – 1.75 (m, 4H), 1.75 – 1.59 (m, 4H), 1.52 – 1.07 (m, 7H), 0.97 – 0.83 (m, 2H). ^{13}C NMR (101 MHz, $CDCl_3$) δ 77.25, 67.65, 43.75, 35.32, 34.17, 33.38, 32.09, 26.77, 26.49, 26.40, 25.82. GC-MS (*m/z*): 168.1 [M]$^+$, 150.1, 82.1, 71.1, 55.1, 43.1. Data are consistent with those reported in the literature.[5]

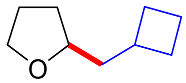

2-(Cyclobutylmethyl)tetrahydrofuran (12): Prepared following the general procedure outlined above using Ir[dF(CF_3)ppy]$_2$(dtbbpy)PF_6 (4.5 mg, 0.004 mmol, 2 mol%), Ni(cod)$_2$ (2.75 mg, 0.01 mmol, 5 mol%), 4,4′-dOMe-bpy (2.4 mg, 0.011 mmol, 5.5 mol%), (bromomethyl)cyclobutane (23 μL, 0.2 mmol), K_2CO_3 (55 mg, 0.4 mmol, 2 equiv.) and THF (4 mL). Pure product was obtained as clear oil in 47% (13.2 mg) yield after column chromatography of the crude reaction mixture (silica gel, gradient 0 to 3% Et_2O/pentane).

^{1}H NMR (400 MHz, $CDCl_3$) δ 3.87 – 3.80 (m, 1H), 3.74 – 3.64 (m, 2H), 2.38 (h, J = 7.9 Hz, 1H), 2.11 – 2.0 (m, 2H), 1.96 – 1.57 (m, 8H), 1.54 – 1.35 (m, 2H). ^{13}C NMR (101 MHz, $CDCl_3$) δ 77.98, 67.59, 42.95, 33.62, 31.58, 28.85, 28.83, 25.86, 18.96. HRMS ESI (m/z): (M+H)$^+$ calcd. for $C_9H_{17}O$, 141.1279; found, 141.1265. GC-MS (*m/z*): 140.1 [M]$^+$, 125.1, 112.1, 84.0, 71.0, 55.1, 43.1

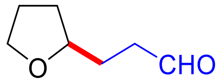

3-(Tetrahydrofuran-2-yl)propanal (13): An oven-dried screw cap reaction tube and a 5 mL vial equipped with a PTFE-coated stir bar were brought into the N_2-filled glove box. Ni(cod)$_2$ (2.75 mg, 0.01 mmol, 5 mol%), 4,4′-dimethoxy-2,2′-bipyridyl (2.4 mg, 0.011 mmol, 5.5 mol%) and THF (3 mL) was added in to 5 mL vial and stirred well for 15 min to give a dark purple color solution (Mixture 1). The other reaction tube was charged with Ir[dF(CF_3)ppy]$_2$(dtbbpy)PF_6 (4.5 mg, 0.004 mmol, 2 mol%), K_2CO_3 (55 mg, 0.4 mmol, 2 equiv.), THF coupling partner (1 mL) and 2-(2-bromoethyl)-1,3-dioxolane (24 μL, 0.2 mmol), and stirred. Mixture 1 was then added to the reaction tube, capped with Teflon septum and parafilmed. The reaction tube was removed from the glove box and irradiated using 34 W blue LEDs while stirring at RT (under fan cooling to keep the reaction at room temperature). After 48 hours, the reaction was concentrated *in vacuo*. To the

crude concentrate, 5 mL of THF and 5 mL 1M HCl were added and stirred well for 1 hour. Dilute it with 25 mL $NaHCO_3$ and extracted with ethyl acetate (3 x 25 mL). The pure product was obtained as a clear oil in 62% (15.7 mg) yield after column chromatography of the crude reaction mixture (silica gel, gradient 5 to 20% DCM/hexane).

^1H NMR (400 MHz, $CDCl_3$) δ 9.78 (s, 1H), 3.88 – 3.79 (m, 2H), 3.73 – 3.67 (m, 1H), 2.62 – 2.46 (m, 2H), 2.03 – 1.74 (m, 5H), 1.52 – 1.43 (m, 1H). ^{13}C NMR (101 MHz, $CDCl_3$) δ 202.45, 78.28, 67.86, 40.88, 31.31, 28.10, 25.89. HRMS ESI (m/z): $(M+H)^+$ calcd. for $C_7H_{13}O_2$, 129.0916; found, 129.0901. GC-MS (*m/z*): 127.9 $[M]^+$, 127.1, 100.0, 84.0, 71.0, 55.0, 43.0, 29.0.

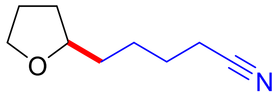

5-(Tetrahydrofuran-2-yl)pentanenitrile (14): Prepared following the general procedure outlined above using Ir[dF(CF_3)ppy]$_2$(dtbbpy)PF_6 (4.5 mg, 0.004 mmol, 2 mol%), Ni(cod)$_2$ (2.75 mg, 0.01 mmol, 5 mol%), 4,4′-dOMe-bpy (2.4 mg, 0.011 mmol, 5.5 mol%), 5-bromopentanenitrile (23.3 µL, 0.2 mmol), K_2CO_3 (55 mg, 0.4 mmol, 2 equiv.) and THF (4 mL). The title compound was obtained as a mixture along with homo-coupled product of alkyl halide (decanedinitrile) in 5:1 ratio as clear oil in 65% (combined yield) yield after column chromatography of the crude reaction mixture (silica gel, gradient 5 to 30% DCM/hexane).

^1H NMR (400 MHz, $CDCl_3$) δ 3.86 – 3.74 (m, 2H), 3.72 – 3.67 (m, 1H), 2.36 – 2.31 (m, 2H), 2.01 – 1.84 (m, 3H), 1.73 - 1.61 (m, 2H), 1.58 – 1.40 (m, 5H). ^{13}C NMR (101 MHz, $CDCl_3$) δ 119.83, 81.61, 78.92, 68.49, 67.80, 34.86, 31.49, 25.79, 25.68, 25.56, 17.23. HRMS ESI (m/z): $(M+H)^+$ calcd. for $C_9H_{16}ON$, 154.1226; found, 154.1224. GC-MS (*m/z*): 153.1 $[M]^+$, 110.1, 97.1, 84.1, 71.0, 55.0, 41.0. Data are consistent with those reported in the literature.[6]

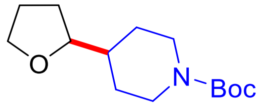

***tert*-Butyl 4-(tetrahydrofuran-2-yl)piperidine-1-carboxylate (15):** Prepared following the general procedure outlined above using Ir[dF(CF_3)ppy]$_2$(dtbbpy)PF_6 (4.5 mg, 0.004 mmol, 2 mol%), Ni(cod)$_2$ (2.75 mg, 0.01 mmol, 5 mol%), 4,4′-dOMe-bpy (2.4 mg, 0.011 mmol, 5.5 mol%), *tert*-butyl 4-bromopiperidine-1-carboxylate (53 mg, 0.2 mmol), K_2CO_3 (55 mg, 0.4 mmol, 2 equiv.) and THF (4 mL). Pure product was obtained as clear oil in 60% (30.6 mg) yield after column chromatography of the crude reaction mixture (silica gel, gradient 1 to 15% EA/hexane).

^1H NMR (400 MHz, $CDCl_3$) δ 4.10 (br, 2H), 3.83 – 3.78 (m, 1H), 3.73 – 3.68 (m, 1H), 3.50 (q, J = 7.3 Hz, 1H), 2.66 – 2.61 (m, 2H), 1.94 – 1.81 (m, 4H), 1.64 – 1.44 (m, 3H), 1.43 (s, 9H), 1.21 – 1.09 (m, 2H). ^{13}C NMR (101 MHz, $CDCl_3$), δ 154.97, 154.92, 83.07, 79.35, 67.91, 43.99, 41.58,

41.16, 29.29, 29.14, 28.58, 28.28, 25.92. HRMS ESI (m/z): $(M+H)^+$ calcd. for $C_{14}H_{26}O_3N$, 256.1907; found, 256.1895. GC-MS (*m/z*): 255.1 $[M]^+$, 198.1, 154.1, 127.1, 84.0, 71.0, 57.1, 41.0.

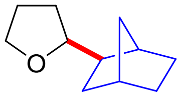

2-(Bicyclo[2.2.1]heptan-2-yl)tetrahydrofuran (16): Prepared following the general procedure outlined above using Ir[dF(CF_3)ppy]$_2$(dtbbpy)PF_6 (4.5 mg, 0.004 mmol, 2 mol%), Ni(cod)$_2$ (2.75 mg, 0.01 mmol, 5 mol%), 4,4′-dOMe-bpy (2.4 mg, 0.011 mmol, 5.5 mol%), exo-2-bromonorbornane (26 µL, 0.2 mmol), K_2CO_3 (55 mg, 0.4 mmol, 2 equiv.) and THF (4 mL). Pure product was obtained as clear oil in 52% (17.2 mg, 1:1 d.r.) yield after column chromatography of the crude reaction mixture (silica gel, gradient 0 to 3% Et_2O/pentane).

^{1}H NMR (400 MHz, $CDCl_3$) (dr, 1:1), δ 3.90 – 3.78 (m, 1H), 3.75 – 3.62 (m, 1H), 3.53 – 3.33 (m, 1H), 2.34 – 1.76 (m, 5H), 1.56 – 1.28 (m, 6H), 1.24 – 0.95 (m, 4H). ^{13}C NMR (101 MHz, $CDCl_3$) (dr, 1:1), δ 83.85, 82.79, 68.00, 67.67, 48.18, 47.89, 39.85, 39.04, 36.86, 36.62, 36.11, 35.39, 35.28, 34.39, 31.09, 30.65, 30.09, 29.65, 29.02, 28.93, 25.94, 25.71. HRMS ESI (m/z): $(M+H)^+$ calcd. for $C_{11}H_{19}O$, 167.1436; found, 167.1422. GC-MS (*m/z*): 166.1 $[M]^+$, 95.0, 81.0, 71.0, 55.0, 41.0. Data are consistent with those reported in the literature.[7]

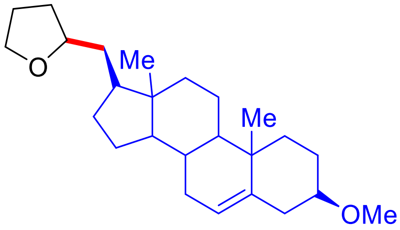

2-(((3R,17S)-3-Methoxy-10,13-dimethyl-2,3,4,7,8,9,10,11,12,13,14,15,16,17-tetradecahydro-1*H*-cyclopenta[*a*]phenanthren-17-yl)methyl)tetrahydrofuran (17): Prepared following the general procedure outlined above using Ir[dF(CF_3)ppy]$_2$(dtbbpy)PF_6 (2.25 mg, 0.002 mmol, 2 mol%), Ni(cod)$_2$ (1.37 mg, 5 µmol, 5 mol%), 4,4′-dOMe-bpy (1.2 mg, 5.5 µmol, 5.5 mol%), 17*β*-(bromomethyl)-3*β* -methoxy-5-androstene (38 mg, 0.1 mmol), K_2CO_3 (27.5 mg, 0.2 mmol, 2 equiv.) and THF (2 mL). Pure product was obtained as solid in 39% (14.5 mg) yield after column chromatography of the crude reaction mixture (silica gel, gradient 0 to 10% Et_2O/pentane).

^{1}H NMR (500 MHz, $CDCl_3$) δ 5.35 (s, 1H), 3.88 – 3.74 (m, 2H), 3.72 – 3.67 (m, 1H), 3.35 (s, 3H), 3.08 – 3.03 (m, 1H), 2.40 – 2.37 (m, 1H), 2.15 (t, *J* = 12.6 Hz, 1H), 2.02 – 1.80 (m, 7H), 1.71 – 1.65 (m, 2H), 1.55 – 1.39 (m, 6H), 1.34 – 1.20 (m, 3H), 1.19 – 0.91 (m, 9H), 0.58 (d, *J* = 3.0 Hz, 3H). ^{13}C NMR (126 MHz, $CDCl_3$) δ 141.05, 121.71, 80.46, 79.17, 78.74, 67.67, 67.53, 55.98, 55.96, 55.76, 50.75, 50.69, 48.28, 47.60, 42.27, 42.11, 38.83, 37.67, 37.61, 37.35, 37.13, 36.37, 36.34, 32.26, 32.16, 32.05, 32.03, 31.52, 28.53, 28.43, 28.14, 25.88, 25.76, 25.01, 24.94, 24.85, 20.96, 19.55, 12.60, 12.56, 12.51. HRMS ESI (m/z): $(M+H)^+$ calcd. for $C_{25}H_{41}O_2$, 373.3101;

found, 373.3092. GC-MS (*m/z*): 372.3 [M]$^+$, 357.2, 340.3, 325.2, 298.1, 281.0, 207.0, 159.1, 145.1, 133.1, 119.0, 105.0, 91.0, 81.0, 71.0, 55.0, 43.0.

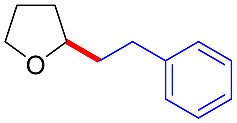

2-Phenethyltetrahydrofuran (18): Prepared following the general procedure outlined above using Ir[dF(CF_3)ppy]$_2$(dtbbpy)PF_6 (4.5 mg, 0.004 mmol, 2 mol%), Ni(cod)$_2$ (2.75 mg, 0.01 mmol, 5 mol%), 4,4′-dOMe-bpy (2.4 mg, 0.011 mmol, 5.5 mol%), (2-bromoethyl)benzene (27.4 µL, 0.2 mmol), K_2CO_3 (55 mg, 0.4 mmol, 2 equiv.) and THF (4 mL). Pure product was obtained as clear oil in 55% (19.5 mg) yield after column chromatography of the crude reaction mixture (silica gel, gradient 0 to 5% Et_2O/pentane).

^{1}H NMR (400 MHz, $CDCl_3$) δ 7.28 (t, *J* = 7.5 Hz, 2H), 7.22 – 7.17 (m, 3H), 3.92 – 3.86 (m, 1H), 3.84 – 3.79 (m, 1H), 3.77 – 3.71 (m, 1H), 2.81 – 2.74 (m, 1H), 2.70 – 2.63 (m, 1H), 2.03 – 1.73 (m, 5H), 1.53 – 1.44 (m, 1H). ^{13}C NMR (101 MHz, $CDCl_3$) δ 142.32, 128.53, 128.44, 125.85, 78.76, 67.81, 37.58, 32.84, 31.48, 25.88. HRMS ESI (m/z): (M+H)$^+$ calcd. for $C_{12}H_{17}O$, 177.12739; found, 177.12693. GC-MS (*m/z*): 176.1 [M]$^+$, 130.0, 117.0, 104.0, 91.0, 71.0, 43.0. Data are consistent with those reported in the literature.[8]

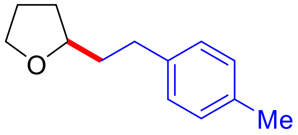

2-(4-Methylphenethyl)tetrahydrofuran (19): Prepared following the general procedure outlined above using Ir[dF(CF_3)ppy]$_2$(dtbbpy)PF_6 (4.5 mg, 0.004 mmol, 2 mol%), Ni(cod)$_2$ (2.75 mg, 0.01 mmol, 5 mol%), 4,4′-dOMe-bpy (2.4 mg, 0.011 mmol, 5.5 mol%), 1-(2-bromoethyl)-4-methylbenzene (30.5 µL, 0.2 mmol), K_2CO_3 (55 mg, 0.4 mmol, 2 equiv.) and THF (4 mL). Pure product was obtained as clear oil in 64% (24.5 mg) yield after column chromatography of the crude reaction mixture (silica gel, gradient 0 to 5% Et_2O/pentane).

^{1}H NMR (400 MHz, $CDCl_3$) δ 7.11 (s, 4H), 3.94 – 3.78 (m, 2H), 3.74 (q, *J* = 7.5 Hz, 1H), 2.77 – 2.70 (m, 1H), 2.67 – 2.60 (m, 1H), 2.33 (s, 3H), 2.04 – 1.71 (m, 5H), 1.53 – 1.44 (m, 1H). ^{13}C NMR (101 MHz, $CDCl_3$) δ 139.21, 135.25, 129.12, 128.39, 78.78, 67.78, 37.68, 32.37, 31.47, 25.86, 21.12. HRMS ESI (m/z): (M+H)$^+$ calcd. for $C_{13}H_{19}O$, 191.1430; found, 191.1427. GC-MS (*m/z*): 190.1 [M]$^+$, 157.1, 144.1, 131.1, 117.1, 105.1, 91.0, 71.0, 43.0. Data are consistent with those reported in the literature.[6]

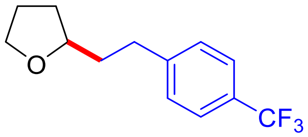

2-(4-(Trifluoromethyl)phenethyl)tetrahydrofuran (20): Prepared following the general procedure outlined above using Ir[dF(CF_3)ppy]$_2$(dtbbpy)PF_6 (4.5 mg, 0.004 mmol, 2 mol%), Ni(cod)$_2$ (2.75 mg, 0.01 mmol, 5 mol%), 4,4′-dOMe-bpy (2.4 mg, 0.011 mmol, 5.5 mol%), 1-(2-bromoethyl)-4-(trifluoromethyl)benzene (34 µL, 0.2 mmol), K_2CO_3 (55 mg, 0.4 mmol, 2 equiv.) and THF (4 mL). Pure product was obtained as clear oil in 63% (30.7 mg) yield after column chromatography of the crude reaction mixture (silica gel, gradient 0 to 5% Et_2O/pentane).

^{1}H NMR (400 MHz, $CDCl_3$) δ 7.52 (d, J = 8.0 Hz, 2H), 7.31 (d, J = 8.0 Hz, 2H), 3.92 – 3.86 (m, 1H), 3.85 – 3.70 (m, 2H), 2.85 – 2.79 (m, 1H), 2.77 – 2.66 (m, 1H), 2.03 – 1.74 (m, 5H), 1.52 – 1.43 (m, 1H). ^{13}C NMR (101 MHz, $CDCl_3$) δ 146.48, 128.84, 128.26 (q, $J_{C–F}$ = 32.32 Hz), 125.36 (q, $J_{C–F}$ = 4.04 Hz), 124.51 (q, $J_{C–F}$ = 272.7 Hz), 78.44, 67.86, 37.26, 32.69, 31.49, 25.87. ^{19}F NMR (377 MHz, $CDCl_3$) δ -62.29. HRMS ESI (m/z): $(M-H)^+$calcd. for $C_{13}H_{14}OF_3$, 243.0991; found, 243.0984. GC-MS (*m/z*): 244.1 $[M]^+$, 225.1, 172.0, 159.0, 133.0, 109.0, 98.0, 84.0, 71.0, 55.0, 43.0. Data are consistent with those reported in the literature.[6]

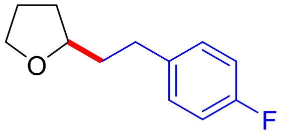

2-(4-Fluorophenethyl)tetrahydrofuran (21): Prepared following the general procedure outlined above using Ir[dF(CF_3)ppy]$_2$(dtbbpy)PF_6 (4.5 mg, 0.004 mmol, 2 mol%), Ni(cod)$_2$ (2.75 mg, 0.01 mmol, 5 mol%), 4,4′-dOMe-bpy (2.4 mg, 0.011 mmol, 5.5 mol%), 1-(2-bromoethyl)-4-fluorobenzene (28 µL, 0.2 mmol), K_2CO_3 (55 mg, 0.4 mmol, 2 equiv.) and THF (4 mL). Pure product was obtained as clear oil in 60% (23.2 mg) yield after column chromatography of the crude reaction mixture (silica gel, gradient 0 to 5% Et_2O/pentane).

^{1}H NMR (400 MHz, $CDCl_3$) δ 7.15 (t, J = 6.8 Hz, 2H), 6.95 (t, J = 8.5 Hz, 2H), 3.88 (q, J = 7.4 Hz, 1H), 3.83 – 3.70 (m, 2H), 2.77 – 2.70 (m, 1H), 2.67 – 2.59 (m, 1H), 2.00 – 1.70 (m, 5H), 1.51 – 1.42 (m, 1H). ^{13}C NMR (101 MHz, $CDCl_3$) δ 161.32 (d, $J_{C–F}$ = 244.42 Hz), 137.87 (d, $J_{C–F}$ = 3.03 Hz), 129.81 (d, $J_{C–F}$ = 8.08 Hz), 115.13 (d, $J_{C–F}$ = 20.2 Hz), 78.56, 67.81, 37.69, 32.01, 31.48, 25.86. ^{19}F NMR (377 MHz, $CDCl_3$) δ -117.95. HRMS ESI (m/z): $(M+H)^+$ calcd. for $C_{12}H_{16}OF$, 195.1179; found, 195.1174. GC-MS (*m/z*): 194.1 $[M]^+$, 161.1, 148.1, 135.0, 122.0, 109.0, 98.0, 83.0, 71.0, 43.0.

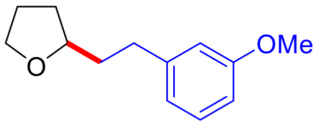

2-(3-Methoxyphenethyl)tetrahydrofuran (22): Prepared following the general procedure outlined above using Ir[dF(CF_3)ppy]$_2$(dtbbpy)PF_6 (4.5 mg, 0.004 mmol, 2 mol%), Ni(cod)$_2$ (2.75 mg, 0.01 mmol, 5 mol%), 4,4′-dOMe-bpy (2.4 mg, 0.011 mmol, 5.5 mol%), 1-(2-bromoethyl)-3-

methoxybenzene (31.4 µL, 0.2 mmol), K_2CO_3 (55 mg, 0.4 mmol, 2 equiv.) and THF (4 mL). Pure product was obtained as clear oil in 61% (25 mg) yield after column chromatography of the crude reaction mixture (silica gel, gradient 0 to 10% Et_2O/pentane).

^{1}H NMR (400 MHz, $CDCl_3$) δ 7.20 (t, J = 7.9 Hz, 1H), 6.81 (d, J = 7.7 Hz, 1H), 6.79 – 6.72 (m, 2H), 3.89 (q, J = 7.7 Hz, 1H), 3.84 – 3.80 (m, 4H), 3.74 (q, J = 7.6 Hz, 1H), 2.79 – 2.61 (m, 2H), 2.05 – 1.72 (m, 5H), 1.53 – 1.44 (m, 1H). ^{13}C NMR (101 MHz, $CDCl_3$) δ 159.73, 143.96, 129.37, 120.93, 114.22, 111.19, 78.74, 67.79, 55.23, 37.45, 32.89, 31.46, 25.86. HRMS ESI (m/z): $(M+H)^+$ calcd. for $C_{13}H_{19}O_2$, 207.1379; found, 207.1375. GC-MS (*m/z*): 206.1 $[M]^+$, 147.0, 135.0, 122.1, 107.0, 91.0, 71.0, 43.0.

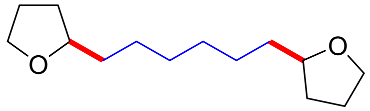

1,6-Bis(tetrahydrofuran-2-yl)hexane (23): Prepared following the general procedure outlined above using Ir[dF(CF_3)ppy]$_2$(dtbbpy)PF_6 (4.5 mg, 0.004 mmol, 2 mol%), Ni(cod)$_2$ (2.75 mg, 0.01 mmol, 5 mol%), 4,4′-dOMe-bpy (2.4 mg, 0.011 mmol, 5.5 mol%), 1,6-dibromohexane (15.4 µL, 0.1 mmol), K_2CO_3 (55 mg, 0.4 mmol, 2 equiv.) and THF (4 mL). Pure product was obtained as clear oil in 49% (11 mg) yield after column chromatography of the crude reaction mixture (silica gel, gradient 0 to 10% Et_2O/pentane).

^{1}H NMR (400 MHz, $CDCl_3$) δ 3.84 (q, J = 7.3 Hz, 2H), 3.79 – 3.67 (m, 4H), 1.99 – 1.78 (m, 6H), 1.6 – 1.5 (m, 2H), 1.46 – 1.30 (m, 12H). ^{13}C NMR (101 MHz, $CDCl_3$)) δ 79.59, 67.73, 35.85, 31.51, 29.84, 26.47, 25.86. HRMS ESI (m/z): $(M+H)^+$ calcd. for $C_{14}H_{27}O_2$, 227.2005; found, 227.2000. GC-MS (*m/z*): 226.2 $[M]^+$, 183.2, 97.0, 84.0, 71.0, 55.1, 43.1. Data are consistent with those reported in the literature.[9]

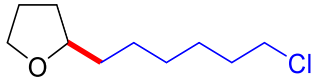

2-(6-Chlorohexyl)tetrahydrofuran (24): Prepared following the general procedure outlined above using Ir[dF(CF_3)ppy]$_2$(dtbbpy)PF_6 (4.5 mg, 0.004 mmol, 2 mol%), Ni(cod)$_2$ (2.75 mg, 0.01 mmol, 5 mol%), 4,4′-dOMe-bpy (2.4 mg, 0.011 mmol, 5.5 mol%), 1-bromo-6-chlorohexane (29.8 µL, 0.2 mmol), K_2CO_3 (55 mg, 0.4 mmol, 2 equiv.) and THF (4 mL). Pure product was obtained as clear oil in 61% (23.2 mg) yield after column chromatography of the crude reaction mixture (silica gel, gradient 0 to 5% Et_2O/pentane).

^{1}H NMR (400 MHz, $CDCl_3$) δ 3.87 – 3.81 (m, 1H), 3.79 – 3.66 (m, 2H), 3.51 (t, J = 6.8 Hz, 2H), 1.99 – 1.91 (m, 1H), 1.89 – 1.81 (m, 2H), 1.75 (p, J = 6.9 Hz, 2H), 1.59 – 1.32 (m, 9H). ^{13}C NMR (101 MHz, $CDCl_3$)) δ 79.43, 67.73, 45.23, 35.73, 32.67, 31.51, 29.08, 26.95, 26.35, 25.83. HRMS ESI (m/z): $(M+H)^+$ calcd. for $C_{10}H_{20}OCl$, 191.1197; found, 191.1193. GC-MS (*m/z*): 189.8 $[M]^+$, 71.0, 56.1, 43.1. Data are consistent with those reported in the literature.[3]

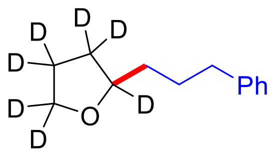

2-(3-Phenylpropyl)tetrahydrofuran-2,3,3,4,4,5,5-d$_7$ (25): Prepared following the general procedure outlined above using Ir[dF(CF_3)ppy]$_2$(dtbbpy)PF_6 (2.25 mg, 0.002 mmol, 2 mol%), Ni(cod)$_2$ (1.37 mg, 5 μmol, 5 mol%), 4,4′-dOMe-bpy (1.2 mg, 5.5 μmol, 5.5 mol%), (3-bromopropyl)benzene (15.2 μL, 0.1 mmol), K_2CO_3 (27.5 mg, 0.2 mmol, 2 equiv.) and d^8-THF (2 mL). Pure product was obtained as clear oil in 57% (11.2 mg) yield after column chromatography of the crude reaction mixture (silica gel, gradient 0 to 5% Et_2O/pentane).

^{1}H NMR (400 MHz, $CDCl_3$) δ 7.31 (t, J = 7.6 Hz, 2H), 7.2 – 7.16 (m, 3H), 2.7 – 2.6 (m, 2H), 1.82 – 1.47 (m, 4H). ^{13}C NMR (101 MHz, $CDCl_3$) δ 142.60, 128.55, 128.37, 125.79, 79.01 – 78.57 (m), 67.13– 66.71 (m), 36.11, 35.28, 30.67 – 30.28 (m), 28.26, 25.20 – 24.80 (m). HRMS ESI (m/z): $(M-H)^+$ calcd. for $C_{13}H_{10}D_7O$, 196.1718; found, 196.1705. GC-MS (m/z): 197.2 $[M]^+$, 179.2, 132.0, 104.1, 91.0, 78.1, 65.1, 50.1.

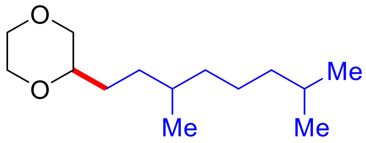

2-(3,7-Dimethyloctyl)-1,4-dioxane (26): An oven-dried screw cap reaction tube and a 5 mL vial equipped with a PTFE-coated stir bar were brought into the N_2-filled glove box. Ni(cod)$_2$ (2.75 mg, 0.01 mmol, 5 mol%), 4,4′-dimethoxy-2,2′-bipyridyl (2.4 mg, 0.011 mmol, 5.5 mol%) and 1,4-dioxane (3 mL) was added in to 5 mL vial and stirred well for 15 min to give a deep purple color solution (Mixture 1). The other reaction tube was charged with Ir[dF(CF_3)ppy]$_2$(dtbbpy)PF_6 (4.5 mg, 0.004 mmol, 2 mol%), K_2CO_3 (55 mg, 0.4 mmol, 2 equiv.), 1,4-dioxane (1 mL), 1-bromo-3,7-dimethyloctane (41.4 μL, 0.2 mmol), and stirred. Mixture 1 was then added to the reaction tube, capped with Teflon septum and parafilmed. The reaction tube was removed from the glove box and irradiated using 34 W x 2 blue LEDs while stirring at RT (under fan cooling to keep the reaction at room temperature). After 96 hours, the reaction was filtered through a small bed of celite and silica and concentrated *in vacuo*. The pure product was obtained as a clear oil in 24% (11 mg) yield after column chromatography of the crude reaction mixture (silica gel, gradient 0 to 10% Et_2O/pentane).

^{1}H NMR (400 MHz, $CDCl_3$) δ 3.78 – 3.67 (m, 4H), 3.59 (td, J = 11.2, 3.3 Hz, 1H), 3.52 – 3.46 (m, 1H), 3.29 – 3.24 (m, 1H), 1.56 – 1.02 (m, 12H), 0.86 – 0.84 (m, 9H). ^{13}C NMR (101 MHz, $CDCl_3$)) δ 76.06, 76.00, 71.62, 71.59, 67.03, 66.71, 39.43, 37.26, 37.22, 33.00, 32.94, 32.41, 29.46, 29.43, 28.10, 24.89, 24.86, 22.85, 22.76, 19.69, 19.63. HRMS ESI (m/z): $(M-H)^+$ calcd. for

$C_{14}H_{27}O_2$, 227.2005; found, 227.1999. GC-MS (*m/z*): 228.1 $[M]^+$, 166.2, 143.2, 126.1, 110.1, 95.1, 87.0, 69.0, 57.1, 43.0.

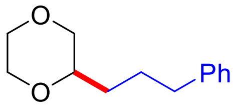

2-(3-Phenylpropyl)-1,4-dioxane (27): An oven-dried screw cap reaction tube and a 5 mL vial equipped with a PTFE-coated stir bar were brought into the N_2-filled glove box. Ni(cod)$_2$ (2.75 mg, 0.01 mmol, 5 mol%), 4,4′-dimethoxy-2,2′-bipyridyl (2.4 mg, 0.011 mmol, 5.5 mol%) and 1,4-dioxane (3 mL) was added in to 5 mL vial and stirred well for 15 min to give a deep purple color solution (Mixture 1). The other reaction tube was charged with Ir[dF(CF$_3$)ppy]$_2$(dtbbpy)PF$_6$ (4.5 mg, 0.004 mmol, 2 mol%), K_2CO_3 (55 mg, 0.4 mmol, 2 equiv.), 1,4-dioxane (1 mL), (3-bromopropyl)benzene (30.4 µL, 0.2 mmol), and stirred. Mixture 1 was then added to the reaction tube, capped with Teflon septum and parafilmed. The reaction tube was removed from the glove box and irradiated using 34 W x 2 blue LEDs while stirring at RT (under fan cooling to keep the reaction at room temperature). After 96 hours, the reaction was filtered through a small bed of celite and silica and concentrated *in vacuo*. The pure product was obtained as a clear oil in 26% (10.7 mg) yield after column chromatography of the crude reaction mixture (silica gel, gradient 0 to 15% Et_2O/pentane).

^{1}H NMR (500 MHz, $CDCl_3$) δ 7.27 (t, *J* = 8 Hz, 2H), 7.19 – 7.16 (m, 3H), 3.77 – 3.68 (m, 4H), 3.61 – 3.52 (m, 2H), 3.26 (t, *J* = 8 Hz, 2H), 2.62 (t, *J* = 8 Hz, 2H), 1.83 – 1.74 (m, 1H), 1.69 – 1.62 (m, 1H), 1.49 – 1.41 (m, 1H), 1.39 – 1.32 (m, 1H). ^{13}C NMR (126 MHz, $CDCl_3$) δ 142.21, 128.54, 128.45, 125.92, 75.43, 71.48, 66.99, 66.68, 35.96, 31.35, 27.04. HRMS ESI (m/z): $(M-H)^+$ calcd. for $C_{13}H_{17}O_2$, 205.1223; found, 205.1218. GC-MS (*m/z*): 206.1 $[M]^+$, 144.1, 104.0, 91.0, 65.0. Data are consistent with those reported in the literature.[3]

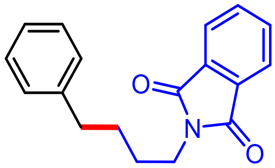

2-(4-phenylbutyl)isoindoline-1,3-dione (28): An oven-dried screw cap reaction tube and a 5 mL vial equipped with a PTFE-coated stir bar were brought into the N_2-filled glove box. $NiCl_2$ glyme (11 mg, 0.05 mmol, 10 mol%), 4,4′-di-*tert*-butyl-2,2′-dipyridyl (14.8 mg, 0.055 mmol, 11 mol%) and benzene (3.5 mL) was added in to 5 mL vial and stirred well for 15 min (Mixture 1). The other reaction tube was charged with Ir[dF(CF$_3$)ppy]$_2$(dtbbpy)PF$_6$ (11.3 mg, 0.01 mmol, 2 mol%), $NaHCO_3$ (84 mg, 1 mmol, 2 equiv.), *N*-(3-bromopropyl)phthalimide (134 mg, 0.5 mmol, 1 equiv.), toluene (530 µL, 5 mmol, 10 equiv.), benzene (0.5 mL), and stirred. Mixture 1 was then added to the reaction tube, capped with Teflon septum and parafilmed. The reaction tube was removed from

the glove box, and irradiated using 34 W x 2 blue LEDs while stirring at RT (under fan cooling to keep the reaction at room temperature). After 96 hours, the reaction was filtered through a small bed of celite and silica and concentrated *in vacuo*. The pure product was obtained as a white solid in 31% (43 mg) yield after the column chromatography of the crude reaction mixture (silica gel, gradient 0 to 5% EtOAc/petether). Care should be taken while doing column chromatography since a small amount of dehydrogenated product is seen which is getting eluted almost in the same polarity. Although an R_f of 0.5 in 15% EtOAc/petether is seen, a long column was packed with the polarity not exceeding 4 - 5% EtOAc/petether was chosen to isolate the product in pure form by running the column slowly for a long time.

^{1}H NMR (400 MHz, $CDCl_3$) δ 7.83 (dd, *J* = 5.5, 3.0 Hz, 2H), 7.70 (dd, *J* = 5.5, 3.0 Hz, 2H), 7.30 – 7.23 (m, 2H), 7.21 – 7.13 (m, 3H), 3.71 (t, *J* = 6.9 Hz, 2H), 2.66 (t, *J* = 7.3 Hz, 2H), 1.79 – 1.61 (m, 4H). ^{13}C NMR (101 MHz, $CDCl_3$)) δ 168.54, 142.09, 133.98, 132.25, 128.53, 128.43, 125.91, 123.29, 37.90, 35.50, 28.75, 28.31. GC-MS (*m/z*): calcd. for $C_{18}H_{17}NO_2$ $[M]^+$ 279.1, found 279.1. Data are consistent with those reported in the literature.[10]

4. Preparation of Ni(dtbbpy)alkyl bromide complex mixture[11-12]

All reagents were prepared in-stock solutions (volumetrically) inside a nitrogen-filled glove box. For the absorption measurements, an appropriate amount of analyte dispensed in THF was added to 2 mL quartz cuvettes, equipped with PTFE stoppers, and sealed with parafilm inside nitrogen-filled glove-box, removed from the glovebox, and the spectrum was collected. Initially, a 1:1 mixture of $Ni(cod)_2$ and 4,4′-di-*tert*-butyl-2,2′-bipyridyl (dtbbpy) in THF was stirred for 30 min under argon to give deep purple color with broad absorption at 565.5 nm confirming the formation of (dtbbpy)Ni(cod). To that, (3-bromopropyl)benzene (1.1 equiv.) was added and the mixture was stirred until the reaction mixture has changed from deep purple to light color. Complete consumption of Ni(dtbbpy)(cod) was confirmed by absorption measurements which showed the disappearance of the broad absorption of Ni(dtbbpy)(cod) (λ_{max} at 565.5 nm) and the appearance of a broad absorption with λ_{max} at 470 nm due to metal-to-ligand-charge-transfer (^{1}MLCT) (Supplementary Fig. 1). Another strong absorption band in the ultraviolet region (λ_{max} = 283 nm) due to ligand-centered $\pi \rightarrow \pi^*$ transition is also seen.

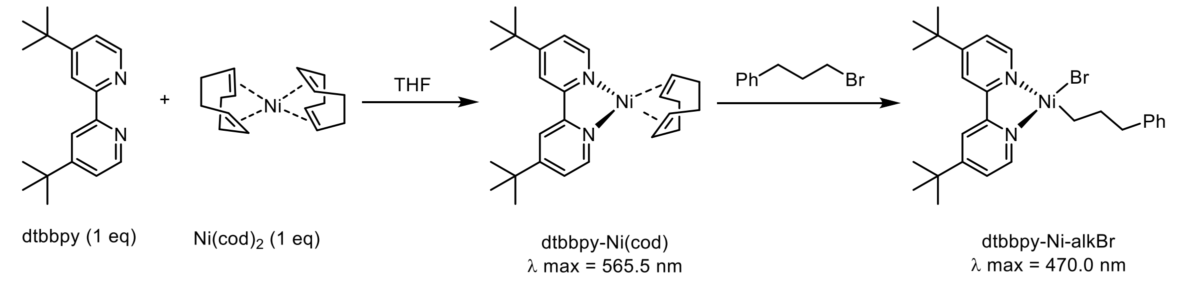

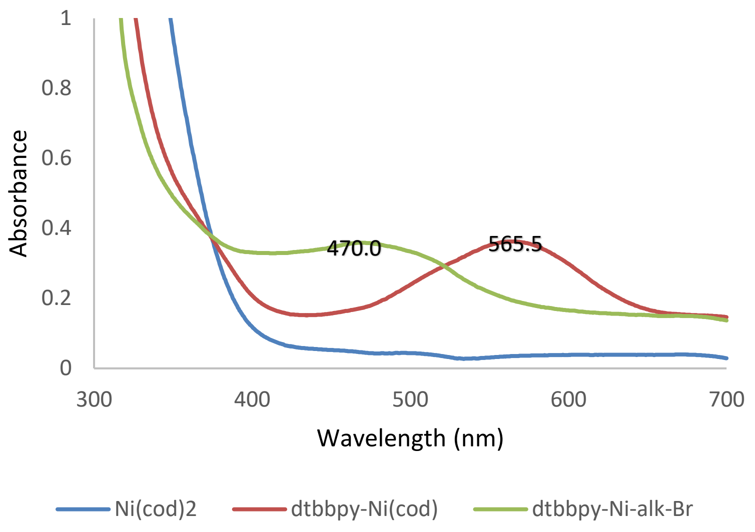

Supplementary Figure 1. The electronic absorption spectrum of (dtbbpy)Ni-alkyl bromide mixture (2.5 × 10^{-4} M) in THF in comparison with $Ni(cod)_2$ and dtbbpy-Ni(cod).

5. Preparation of Ni(dOMebpy)alkyl bromide complex mixture[11-12]

Initially, a 1:1 mixture of $Ni(cod)_2$ and 4,4′-dimethoxy-2,2′-bipyridyl (dOMebpy) in THF was stirred for 30 min under argon to give deep purple color with broad absorption (λ_{max} at 567.5 nm) conforming to the formation of (dOMebpy)Ni(cod). To that, (bromomethyl)cyclobutane (1.1 equiv.) was added and the mixture was stirred until the reaction mixture has changed from deep purple to light green color. Complete consumption of Ni(dOMebpy)(cod) was confirmed by absorption measurements which showed the disappearance of the broad absorption of Ni(dOMebpy)(cod) (λ_{max} at 567.5 nm) and the appearance of a broad absorption with λ_{max} at 452 nm which we expect due to metal-to-ligand-charge-transfer (^{1}MLCT) (Supplementary Fig. 2).

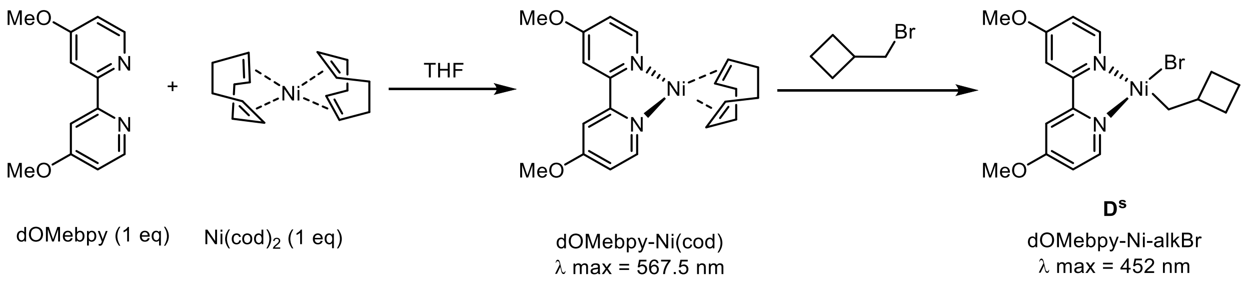

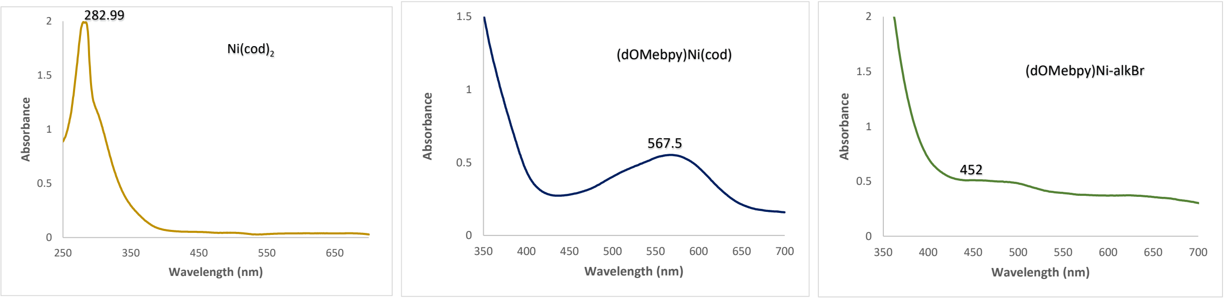

Supplementary Figure 2. The electronic absorption spectrum of (dOMebpy)Ni-alkyl bromide **D**^{s} mixture (5.0×10^{-4} M) in THF in comparison with $Ni(cod)_2$ and dOMebpy-Ni(cod).

6. Steady-state Stern-Volmer quenching experiments

Emission spectra were collected on a fluoromax-4 Spectrophotometer with excitation and emission slit widths of 2 nm. Quenching experiments were carried out using a $1x10^{-5}$ M solution of Ir[dF(CF_3)ppy]$_2$(dtbbpy)PF_6 (PS1) in THF and variable concentrations of quencher (dOMebpy)Ni-alkBr D^s (in situ prepared) dispensed in THF (2.5, 5.0, 7.5, 10, 12.5, 15, 17.5 μM). The samples were prepared in 2 mL quartz cuvettes, equipped with PTFE stoppers, and sealed with parafilm inside a nitrogen-filled glove box, removed from the glove box and an emission spectrum was collected. Samples were excited at 380 nm and the intensity of emission was monitored at 472 nm expressed as the ratio I_0/I, where I_0 is the emission intensity of PS1 at 472 nm in the absence of a quencher and I is the observed intensity, as a function of the quencher concentration was measured. Fluorescence emission spectra and Stern-Volmer plot are given in the Figures below.

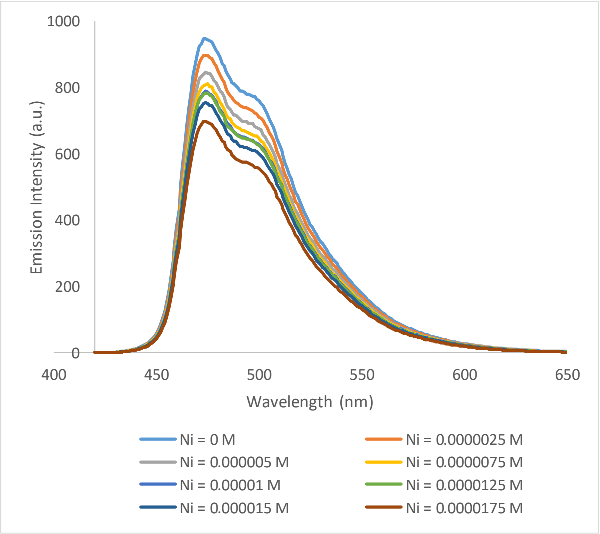

Supplementary Figure 3. Emission spectra of PS1 ($1x10^{-5}$ M) at different concentrations of (dOMebpy)Ni-alkyl bromide D^s (λ_{ex}= 380nm).

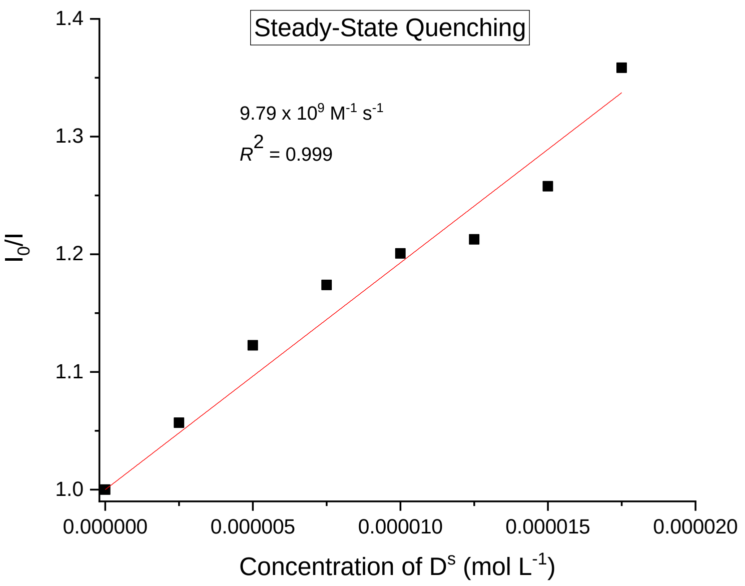

Supplementary Figure 4. The Stern-Volmer plot of PS1 ($1x10^{-5}$ M) at different concentrations of (dOMebpy)Ni-alkyl bromide D^s.

7. Photoluminescence lifetime (Time-resolved Stern-Volmer quenching) experiments

Time-resolved Stern-Volmer quenching experiments were carried out using a 0.0001 M solution of Ir[dF(CF_3)ppy]$_2$(dtbbpy)PF_6 (PS1) in THF and variable concentrations of quencher (dOMebpy)Ni-alkBr D^s (in situ prepared) dispensed in THF (25, 50, 75, 100, 125, 150, 175, 200 μM). The samples were prepared in 2 mL quartz cuvettes, equipped with screw cap PTFE stoppers, and sealed with parafilm inside the argon-filled glove-box. The intensity of the emission peak at 472 nm is expressed as the ratio k_{obs}/k_{GSR}, where k_{GSR} is the decay of PS1 at 472 nm in the absence of a quencher and k_{obs} is the observed decay, as a function of the quencher concentration was measured. An Ar-saturated 0.0001 M solution in THF was used for the determination of the photoluminescence lifetimes of the PS1. Photoluminescence decay traces were acquired based on time-correlated single-photon-counting (TCSPC) techniques using a fluoromax-4 spectrophotometer from Horiba Scientific. A 380 nm diode laser was used as the excitation source. The photoluminescence signals were obtained using an automated motorized monochromator. Time-resolved emission data were fit to a single exponential decay to extract the observed rate constant (k_{obs}). Phosphorescence emission spectra and Stern-Volmer plots for each component are given in below.

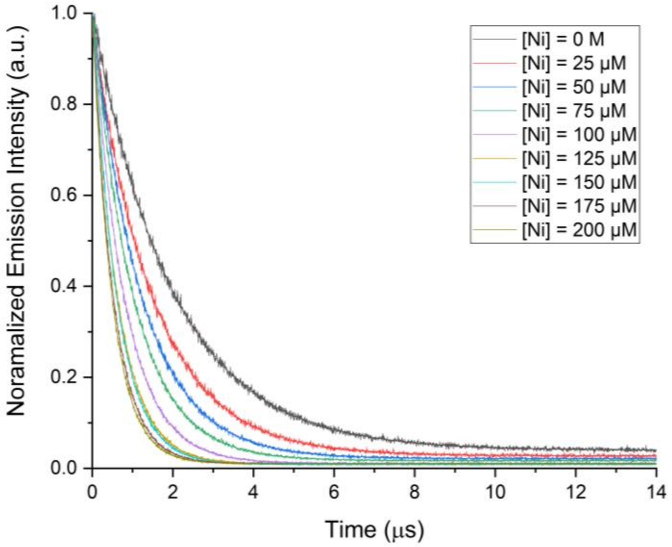

Supplementary Figure 5. Phosphorescence lifetimes of PS1 (0.0001 M) at different concentrations of quencher D^s. Spectroscopic experiments were performed one single time.

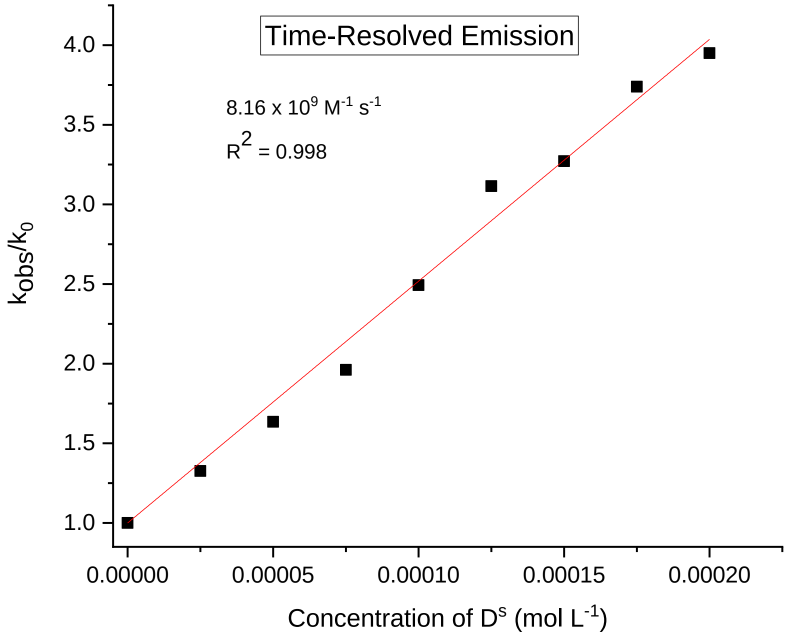

Supplementary Figure 6. Time-resolved Stern-Volmer quenching plot of PS1 (0.0001 M) at different concentrations of D^s.

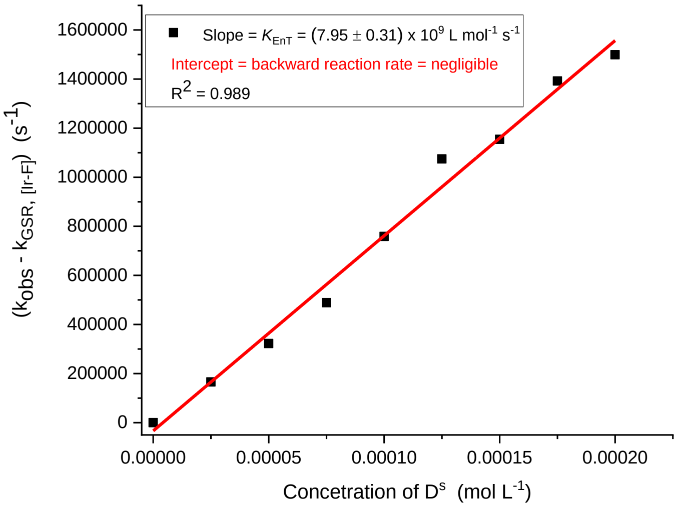

Supplementary Figure 7. A plot of the observed energy transfer rate constant (obs) of *PS1 (0.0001 M) deactivation corrected by its intrinsic ground state recovery (GSR) rate *vs.* different concentrations of quencher D^s. Data were collected by the use of phosphorescence lifetime measurements.

8. Stoichiometric reaction of (dtbbpy)Ni-alkyl bromide complex mixture with THF

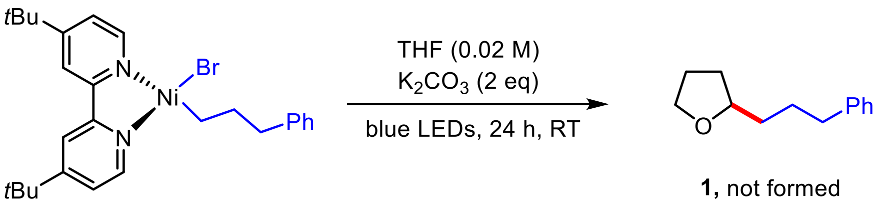

1, not formed

Reaction in presence of visible light: An oven-dried screw cap reaction tube equipped with a PTFE-coated stir bar was brought into an N_2-filled glove box. $Ni(cod)_2$ (27.5 mg, 0.1 mmol, 1 equiv.), 4,4′-di-tert-butyl-2,2′-dipyridyl (26.8 mg, 0.1 mmol, 1 equiv.) and THF (5 mL, 0.02 M) was added and stirred well for 30 min to give a deep purple color solution. To that, (3-bromopropyl)benzene (0.1 mmol) was added and the mixture was stirred until the reaction mixture has changed from deep purple to light color with the disappearance of the broad absorption of Ni(dtbbpy)(cod) (λ_{max} at 565.5 nm) and the appearance of a new broad absorption peak with λ_{max} at 470 nm due to metal-to-ligand-charge-transfer (^{1}MLCT) (Supplementary Fig. 1). To that, K_2CO_3 (27.6 mg, 0.2 mmol, 2 equiv.) was added, capped with Teflon septum, and parafilmed. The reaction tube was removed from the glove box, and irradiated using 34 W blue LEDs while stirring at RT. After 48 hours, the reaction mixture was diluted with ethyl acetate and subjected to Gas Chromatography which does not show product formation.

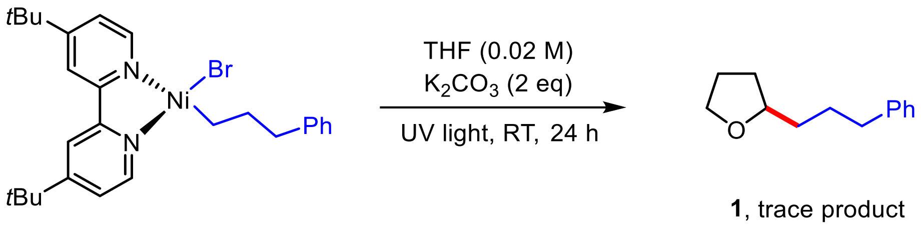

1, trace product

Reaction in presence of ultraviolet light: An oven-dried screw cap reaction tube equipped with a PTFE-coated stir bar was brought into an N_2-filled glove box. Ni(cod)$_2$ (27.5 mg, 0.1 mmol, 1 equiv.), 4,4′-di-tert-butyl-2,2′-dipyridyl (26.8 mg, 0.1 mmol, 1 equiv.) and THF (5 mL, 0.02 M) was added and stirred well for 30 min to give a deep purple color solution. To that, (3-bromopropyl)benzene (0.1 mmol) was added and the mixture was stirred until the reaction mixture has changed from deep purple to light color with the disappearance of the broad absorption of Ni(dtbbpy)(cod) (λ_{max} at 565.5 nm) and the appearance of a new broad absorption peak with λ_{max} at 470 nm due to metal-to-ligand-charge-transfer (^{1}MLCT) (Supplementary Fig. 1) and another strong absorption band in the ultraviolet region (λ_{max} = 283 nm) due to ligand-centered $\pi \rightarrow \pi^*$ transition. To that, K_2CO_3 (27.6 mg, 0.2 mmol, 2 equiv.) was added, capped with Teflon septum, and parafilmed. The reaction tube was removed from the glove box and irradiated using UV light (Rayonet photochemical reactor, 300 nm wavelength) while stirring at RT. After 24 hours, the reaction mixture was diluted with ethyl acetate and subjected to Gas Chromatography which showed only a very trace amount of product.

9. Kinetic isotope effect by intermolecular competition experiment between a mixture of THF and *d*$_8$-THF

Kinetic isotope experiments were carried out to understand the nature of the C–H functionalization. An oven-dried screw cap reaction tube and a 5 mL vial equipped with a PTFE-coated stir bar were brought into the N_2-filled glove box. Ni(cod)$_2$ (1.37 mg, 5 μmol, 5 mol%), 4,4′-dimethoxy-2,2′-bipyridyl (1.2 mg, 5.5 μmol, 5.5 mol%), THF (1.5 mL) and *d*$_8$-THF (1.5 mL) was added in to 5 mL vial and stirred well for 15 min to give a deep purple color solution (Mixture 1). The other reaction tube was charged with Ir[dF(CF$_3$)ppy]$_2$(dtbbpy)PF$_6$ (2.25 mg, 0.002 mmol, 2 mol%), K_2CO_3 (27.5 mg, 0.2 mmol, 2 equiv.), THF (0.5 mL), *d*$_8$-THF (0.5 mL), (3-bromopropyl)benzene (15.2 μL, 0.1 mmol), and stirred. Mixture 1 was then added to the reaction tube, capped with Teflon septum and parafilmed. The reaction tube was removed from the glove box, and irradiated using 34 W blue LEDs while stirring at RT (under fan cooling to keep the reaction at room temperature). After 48 hours, the reaction was filtered through a small bed of celite and silica and concentrated *in vacuo*. Analysis of the crude reaction mixture by NMR spectroscopy gave a difference in product distribution of **1** and **25** in the ratio of 1.7:1.

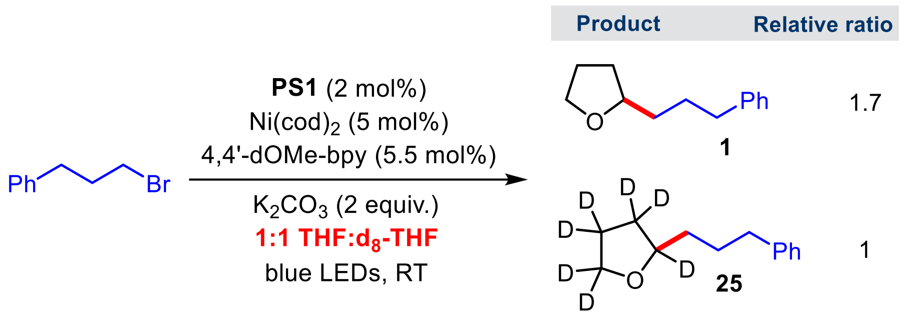

Kinetic isotope effect using rate measurement: An oven-dried screw cap reaction tube and a 5 mL vial equipped with a PTFE-coated stir bar were brought into an N_2-filled glove box. $Ni(cod)_2$ (1.37 mg, 5 µmol, 5 mol%), 4,4′-dimethoxy-2,2′-bipyridyl (1.2 mg, 5.5 µmol, 5.5 mol%), THF (1.5 mL) was added in to 5 mL vial and stirred well for 15 min to give a deep purple color solution (Mixture 1). The other reaction tube was charged with $Ir[dF(CF_3)ppy]_2(dtbbpy)PF_6$ (2.25 mg, 0.002 mmol, 2 mol%), K_2CO_3 (27.5 mg, 0.2 mmol, 2 equiv.), THF (0.5 mL), (3-bromopropyl)benzene (15.2 µL, 0.1 mmol), and stirred. Mixture 1 was then added to the reaction tube along with dodecane as internal standard (1 equiv., 0.1 mmol), capped with Teflon septum and parafilmed. The reaction tube was removed from the glove box, and irradiated using 34 W blue LEDs while stirring at RT (under fan cooling to keep the reaction at room temperature). An aliquot of the reaction mixture was collected under positive pressure of argon at different time intervals and was subjected to GCMS to see the formation of product **1**.

A similar procedure was repeated using the *d*8-THF and an aliquot of the reaction mixture was collected under positive pressure of argon at different time intervals and was subjected to GCMS to see the formation of product **25**.

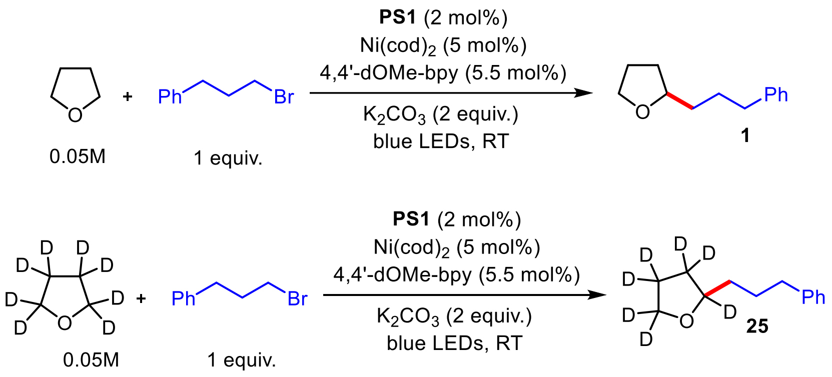

10. Catalytic reaction using Mischler's ketone

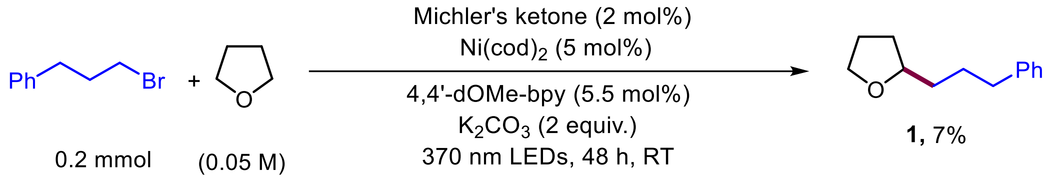

2-(3-Phenylpropyl)tetrahydrofuran (1): An oven-dried screw cap reaction tube and a 5 mL vial equipped with a PTFE-coated stir bar were brought into the N_2-filled glove box. $Ni(cod)_2$ (2.75 mg, 0.01 mmol, 5 mol%), 4,4′-dimethoxy-2,2′-bipyridine (2.4 mg, 0.011 mmol, 5.5 mol%) and THF (3 mL) was added in to 5 mL vial and stirred well for 15 min to give a deep purple color solution (Mixture 1). The other reaction tube was charged with Mischler's ketone (1 mg, 0.004 mmol, 2 mol%), K_2CO_3 (55 mg, 0.4 mmol, 2 equiv.), THF (1 mL), (3-bromopropyl)benzene (30.4 µL, 0.2 mmol) and stirred. Mixture-1 was then added to the reaction tube, capped with Teflon septum and parafilmed. The reaction tube was removed from the glove box, and irradiated using 370 nm LEDs while stirring at RT (under fan cooling to keep the reaction at room temperature). After 48 hours, the reaction was filtered through a small bed of celite and subjected to GC.

11. Dependence of the reaction rate on light intensity

Dependence of the reaction rate on light intensity is calculated by running the reactions under full and half intensity of the light source at different time intervals and the corresponding yield of **1** is measured by GCMS using dodecane as internal standard. The values suggest that more than one photon is involved in the reaction mechanism.

Supplementary Table 4. Yields of **1** measured by GCMS under full and half intensity of the light source at different time intervals using dodecane as internal standard.

Time (h)	At full intensity, product/standard ratio	At half intensity, product/standard ratio
1	0	0
2	0.0271	0.01
4	0.0913	0.029
6	0.187	0.057
8	0.279	0.095

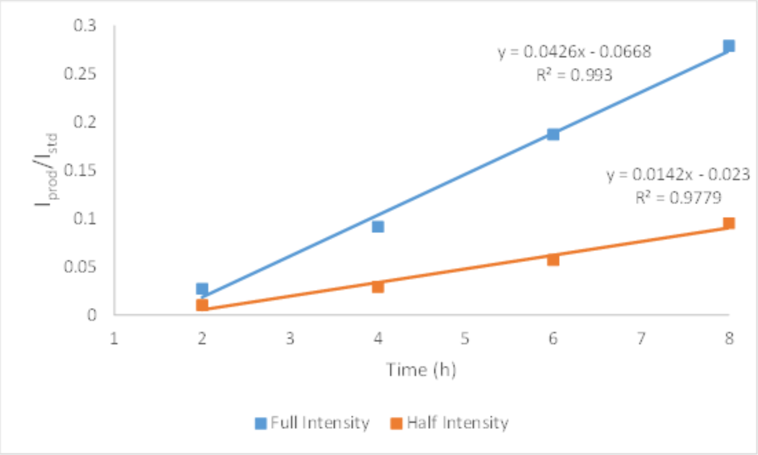

12. Time-resolved absorption studies

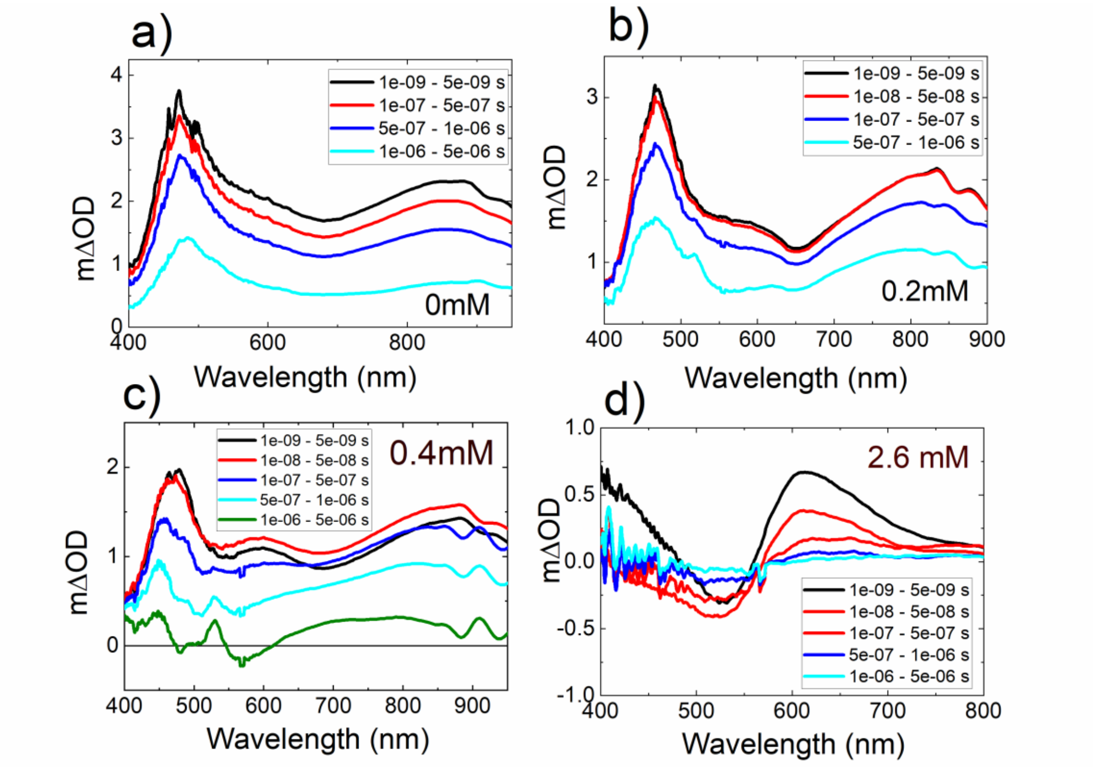

Supplementary Figure 8. a ns-TA spectra of ^{3*}PS1 (0.1 mM) in the absence of quencher D^S. **b-c** ns-TA spectra of a mixture of ^{3*}PS1 and D^S (0.2 and 0.4 mM quencher concentration) indicate the formation of $*D^T$ at 600 nm. **d** ns-TA spectra of a mixture of ^{3*}PS1 (0.1 mM) and D^S (2.6 mM), which exhibits complete quenching of the photocatalyst by D^S, resulting in a new spectral feature at 400 and 600 nm. All measurements were done under an inert atmosphere and excited at 355 nm.

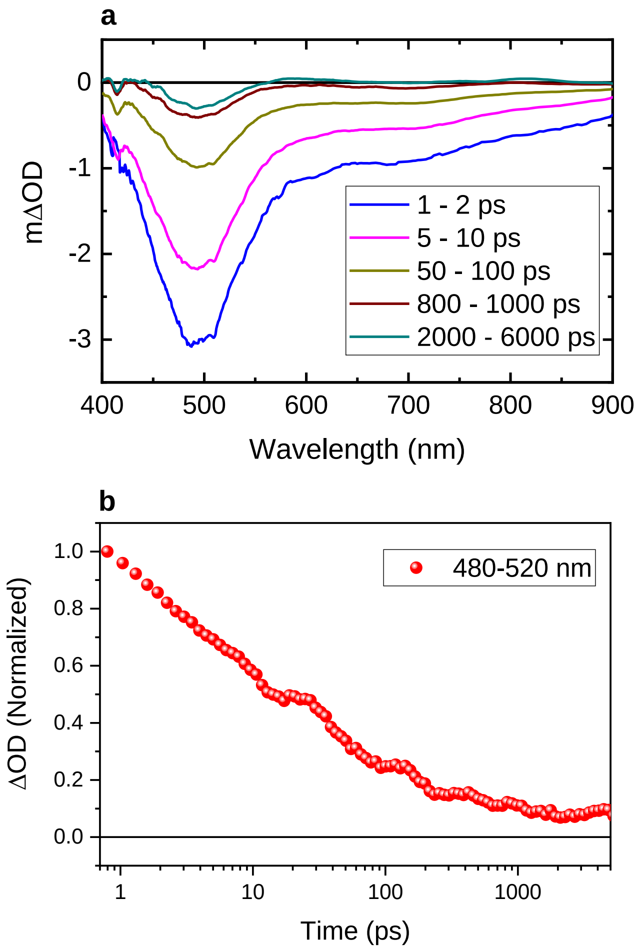

Supplementary Figure 9. a Picosecond TA-spectra, and **b** kinetics of the D^S complex excited at 375 nm in THF solution. The TA spectral evolution shows that most of the excited state population decayed within 1ns.

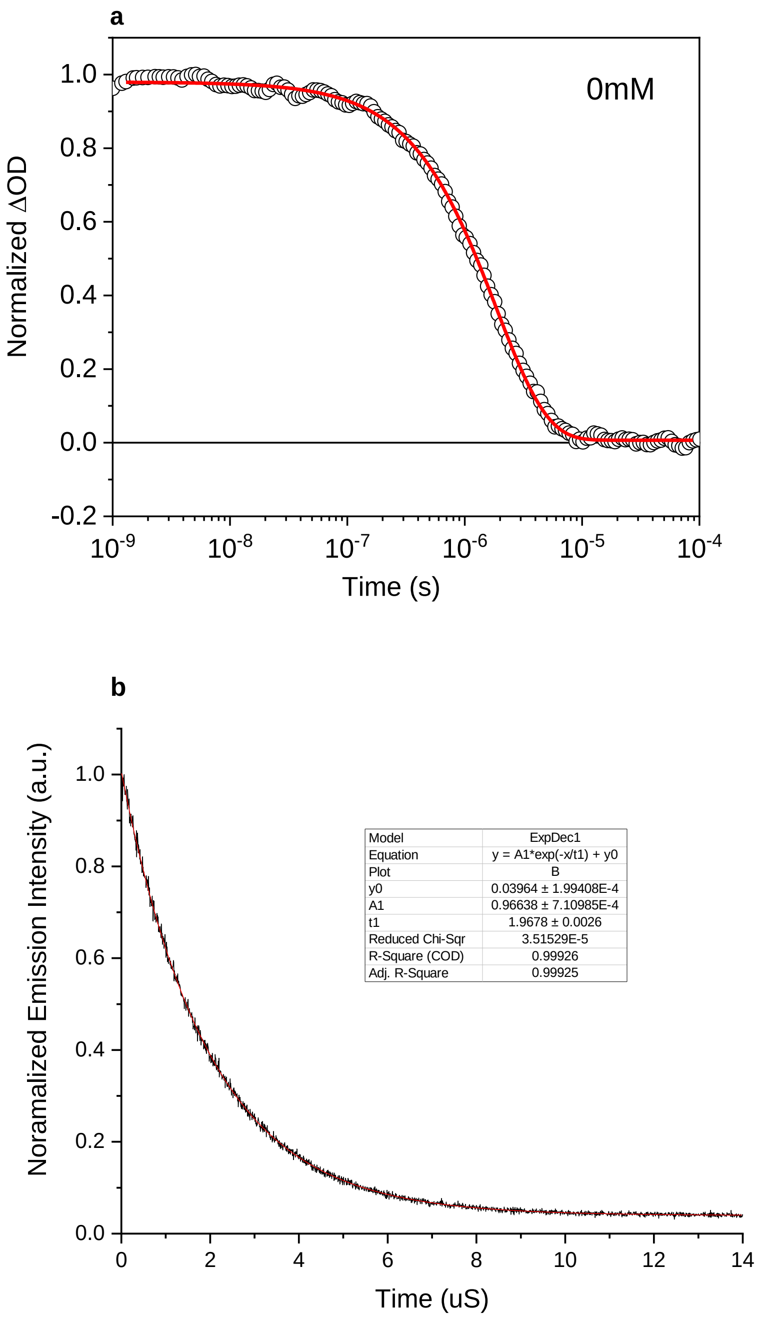

Supplementary Figure 10. a ns-TA kinetics (open symbols) of ^{3*}PS1 (0.1 mM) in the absence of quencher D^S and corresponding mono-exponential fit (red line). The exponential fit yields an inverse rate constant of 1.86±0.003μs. **b** The lifetime of ^{3*}PS determined by TRPL.

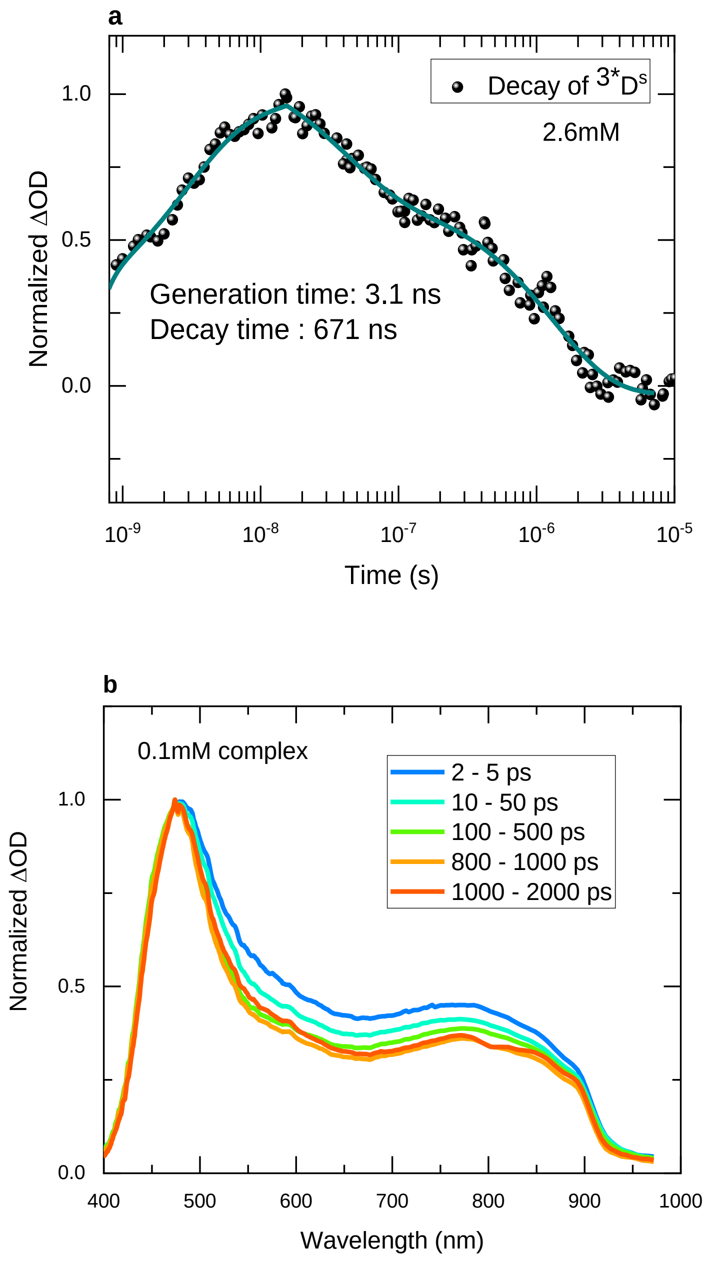

Supplementary Figure 11. a Normalized ns-TA spectra of $*D^T$ with the weighted average decay time of 671 ns. **b** Normalized picosecond TA-spectra 0.1mM complex excited at 375 nm in THF solution. The TA spectral evolution shows a slight emergence of a band at 550-650 nm region after 1ns (red line spectra) indicating the presence of a new species.

13. Picosecond and nanosecond-TA spectroscopy using Ni(dtbbpy)(*o*-tolyl)Cl

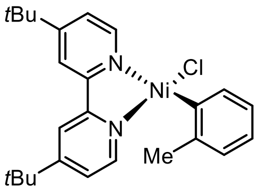

Ni(dtbbpy)(*o*-tolyl)Cl. This compound is prepared by the procedure followed by Doyle and co-workers.[11] An oven-dried screw cap reaction tube equipped with a PTFE-coated stir bar was brought into the N_2-filled glove box. Ni(cod)$_2$ (275 mg, 1 mmol), 4,4′-di-*tert*-butyl-2,2′-bipyridine (268.4 mg, 1 mmol), and THF (2.5 mL) was added and stirred for 1 hour at ambient temperature which gave the deep purple color solution. To that, 2-chlorotoulene (6 mL) was added and left to stir for 20 min. The resulting dark red solution was triturated with pentane to give the precipitate which was filtered on a frit, rinsed with pentane, and dried under high vacuum to give the title compound as a light red powder (367 mg, 81%). The ^{1}H and ^{13}C-NMR are in accord with the literature report. The title compound was used for spectroscopic studies without further purification.

^{1}H NMR (400 MHz, CD_2Cl_2): δ 9.04 (d, *J* = 5.8 Hz, 1H), 7.86 (s, 1H), 7.82 (s, 1H), 7.54 (t, *J* = 5.5 Hz, 2H), 7.16 (d, *J* = 6.1 Hz, 1H), 7.11 (d, *J* = 5.5 Hz, 1H), 6.83-6.75 (m, 3H), 3.06 (s, 3H), 1.43 (s, 9H), 1.35 (s, 9H). ^{13}C NMR (101 MHz, CD_2Cl2): δ 163.93, 162.92, 156.28, 152.89, 151.34, 150.96, 149.31, 142.65, 135.74, 127.48, 123.94, 123.58, 123.24, 122.76, 117.84, 117.04, 35.74, 35.65, 30.43, 30.19, 25.17.

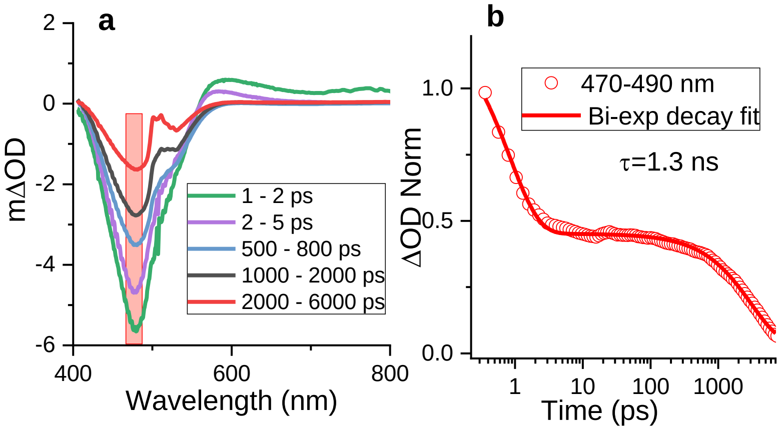

Supplementary Figure 12. a Picosecond TA-spectra, and **b** kinetics of the marked spectral region in Fig. a of the Ni($^{t\text{-Bu}}$bpy) (*o*-Tol)Cl excited at 505 nm in THF solution.

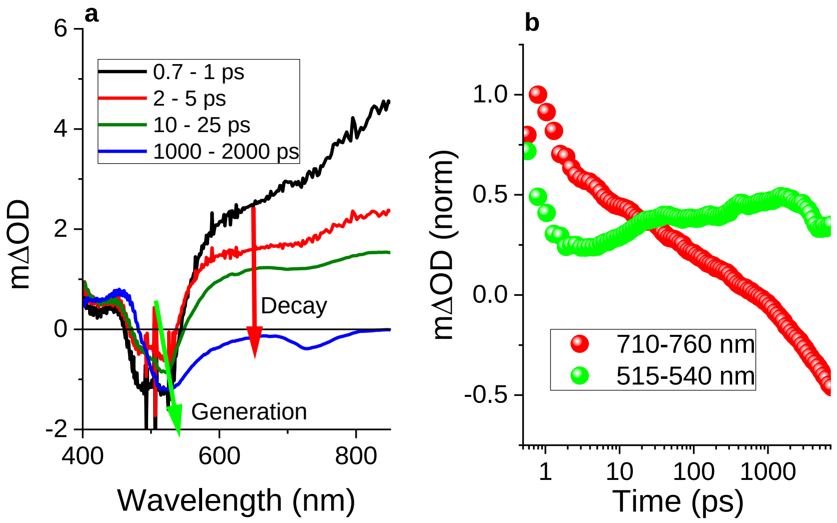

Supplementary Figure 13. a Picosecond TA-spectra, and **b** kinetics of the marked spectral region in Fig. a of the PS1 and Ni($^{t\text{-Bu}}$bpy) (*o*-Tol)Cl excited at 360 nm in THF solution.

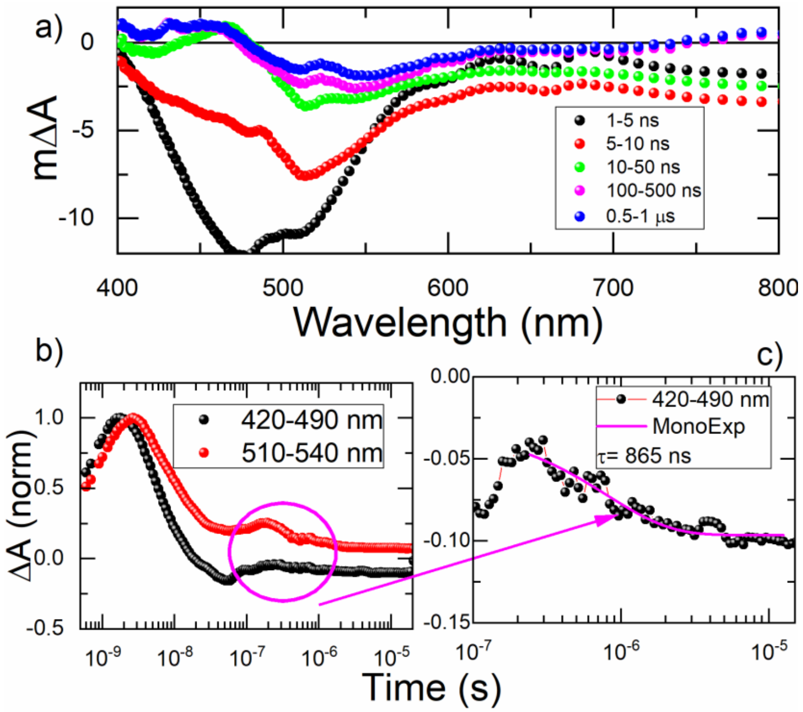

Supplementary Figure 14. a nsTA-spectra, and **b-c** kinetics of the selected spectral region in Fig. a of the PS1-Ni($^{t\text{-Bu}}$bpy) (*o*-Tol)Cl excited at 355 nm in THF solution.

14. Computational methods

All the geometries were optimized with the hybrid *meta*-generalized gradient approximation (*meta*-GGA) method with Gaussian 09 program packages,[13] using M06 functional.[14] The electronic configuration of all the non-metal elements (H, C, N, O, F and Br) were described with the Ahlrichs split-valance polarization basis function Def2-SVP[4] while Ni and Ir are treated with the triple-ζ valence basis set Def2-TZVP.[15] The geometries were optimized without any symmetry constraints. Harmonic force constants were computed at the optimized geometries to characterize the stationary points as minima or saddle points. All transition states were optimized using the default Berny algorithm implemented in the Gaussian09 code.[2] For further validation of energetics, single-point calculations were performed at the same functional, M06 employing Def2-TZVPP[4] basis sets for all atoms. The solvent effects (THF, $\varepsilon = 7.426$) were evaluated implicitly by a self-consistent reaction field (SCRF) approach for all the intermediates and transitions states, using the density-based solvation model, SMD[16] implemented in Gaussian09. Linear response time-dependent DFT (TD-DFT) calculations were carried out on the gas-phase optimized geometry using M06(SMD)/Def2-TZVPP method. Unless otherwise specified, the ΔG was used throughout the text. The ΔG value was obtained by augmenting the ΔE_e energy terms at M06(SMD)/Def2-TZVPP with the respective free energy corrections at the M06/Def2-TZVP(Ni,Ir)/Def2-SVP(C,H,N,O,F,Br) level in gas phase. NBO 3.1 is used for Weinhold's NPA (Natural Population Analysis).[17] All 3D structures were prepared using CYLView.[18]

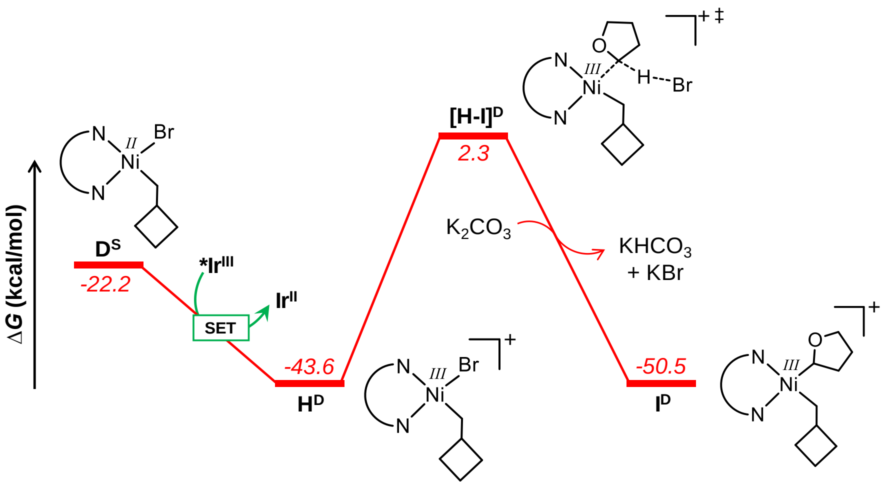

Supplementary Figure 15. Energy profile for HAT step in electron transfer pathway. For energy and other conventions refer to Fig. 4 in the manuscript.

Supplementary Table 5. Details of the vertical excitation for the species D^S. Minimum value of oscillator strength is 0.03. TD-DFT calculated absorption spectra is shown below.

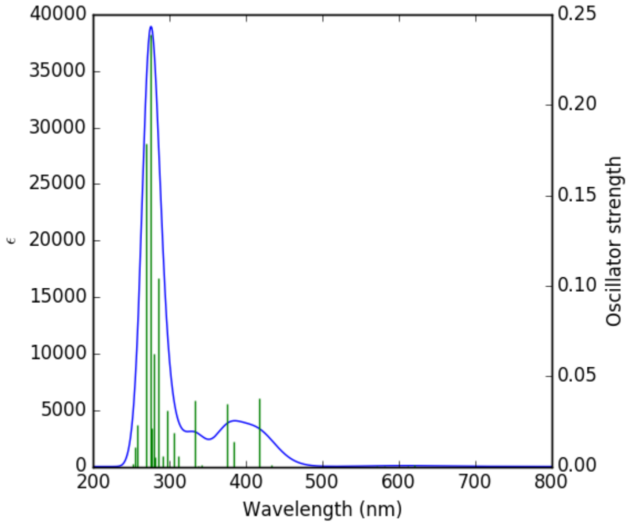

Excited singlet states	f[1]	λ_{max} (nm)[2]	Orbitals (percentage contribution)[3]	
S_6	0.036	427	HOMO-1→LUMO(67%)<br>HOMO→LUMO(24%)	HOMO-3→LUMO(3%)
S_8	0.050	377	HOMO-3→LUMO(84%)<br>HOMO-4→LUMO(3%)	HOMO-2→LUMO(5%)<br>HOMO-1→LUMO(3%)
S_{14}	0.035	306	HOMO-1→LUMO+2(74%)<br>HOMO→LUMO+2(10%)	HOMO-7→LUMO(5%)<br>HOMO-3→LUMO+1(2%)
S_{17}	0.222	294	HOMO-8→LUMO(39%)<br>HOMO-7→LUMO(18%)<br>HOMO-2→LUMO+2(17%)	HOMO-3→LUMO+1(2%)<br>HOMO-3→LUMO+2(7%)<br>HOMO-1→LUMO+2(5%)
S_{18}	0.044	288	HOMO-9→LUMO(12%)<br>HOMO-8→LUMO(28%)<br>HOMO-2→LUMO+2(40%)	HOMO-6→LUMO(3%)<br>HOMO-3→LUMO+1(2%)<br>HOMO-1→LUMO+2(3%)
S_{19}	0.107	285	HOMO-9→LUMO(36%)<br>HOMO-7→LUMO(16%)<br>HOMO-2→LUMO+2(24%	HOMO-6→LUMO(7%)<br>HOMO-4→LUMO(2%)<br>HOMO→LUMO+2(3%)
S_{21}	0.249	277	HOMO-3→LUMO+2(66%)<br>HOMO-8→LUMO(6%)<br>HOMO-5→LUMO(2%)	HOMO-4→LUMO+2(2%)<br>HOMO-2→LUMO+3(4%)<br>HOMO-1→LUMO+3(3%)

[1]The oscillator strength of the transition. [2]Wavelength of the transition. [3]Molecular orbitals involved in the transitions; H = HOMO, L = LUMO. The respective contributions are in parentheses.

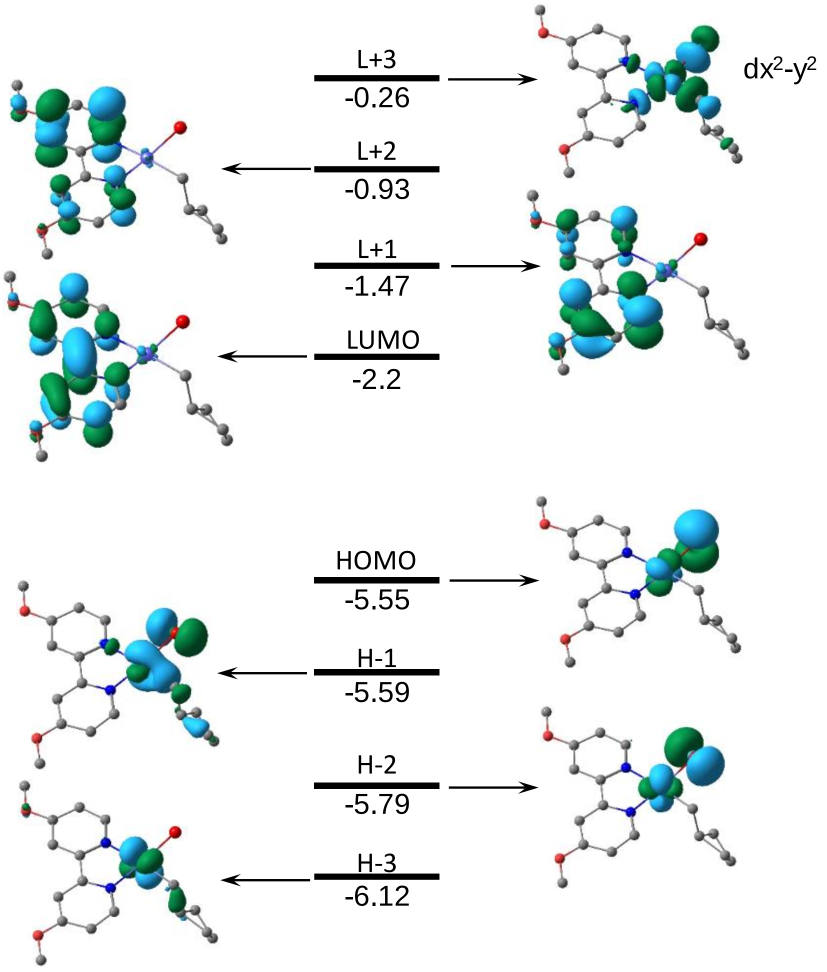

Supplementary Figure 16. KS-Molecular orbitals of D^S in the order HOMO-3, HOMO-2, HOMO-1, HOMO, LUMO, LUMO+1, LUMO+2, LUMO+3. The HOMO's and LUMO's are abbreviated as H and L. The Figure is not drawn to scale. Energy values are in eV.

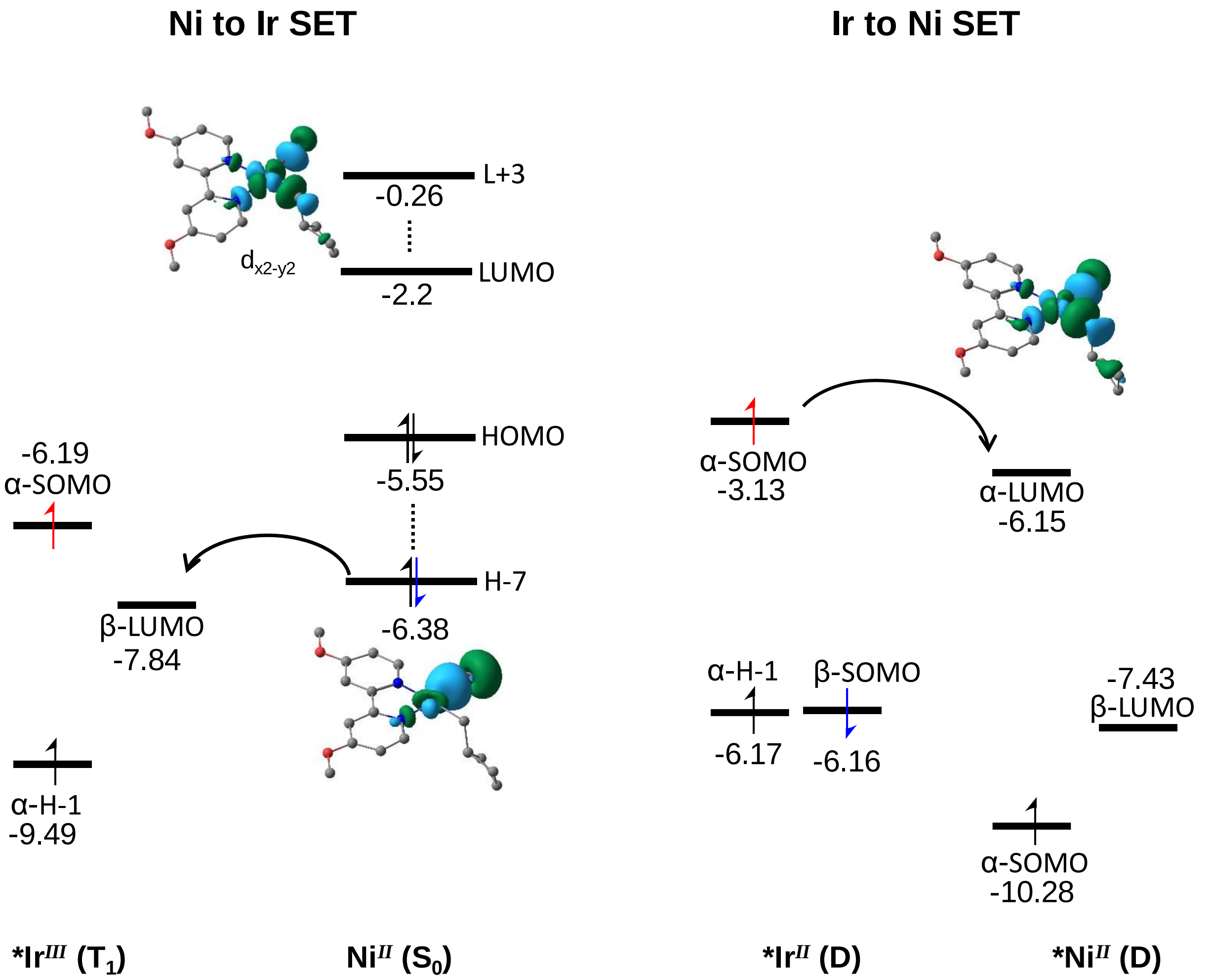

Supplementary Figure 17. Reversible electron transfer for Dexter TTET process. Energy values are in eV. **S**, **D,** and **T** in parenthesis represent singlet, doublet, and triplet electronic states respectively.

Supplementary Discussion

1. Dexter triplet-triplet energy transfer (TTET)

In principle, two types of intermolecular energy transfer (E_nT) mechanisms between *PS(T_1) and D(S_0), the Förster[19] and the Dexter[20] mechanisms, are possible. In the Förster mechanism, PS(T_1) returns to its singlet ground state with the simultaneous excitation of D(S_0) to an excited singlet state. Unfortunately, no excited singlet of Ni(II) is suitable to perform the HAT step because none of the allowed excitations populates the LUMO+3 that corresponds to the σ* Ni-Br bond, see Supplementary Table 5 and Supplementary Fig. 16. Hence the Förster energy transfer is ruled out. Simply, the EnT from a photocatalyst excited triplet state to the Ni(II) singlet ground state will lead to singlet ground state Ir and excited triplet state Ni(II) that represents two spatially separated spin reversal processes (violating Wigner's spin conversion rules)[21] which cannot be described by Förster Resonance EnT mechanism. However, the Dexter E_nT mechanism, which implies a reversal electron transfer to exchange energy between *PS(T_1) and D(S_0) following the eq 1, offers a pathway to the triplet excited state of the N(II) intermediate involving the LUMO+3. As mentioned above, the LUMO+3 corresponds to the σ* Ni-Br bond, which can undergo HAT step to perform the desired reaction.

$$*PS(T_1) + D(S_0) \rightarrow PS(S_0) + *D(T_n) \quad (1)$$

According to equation 1, D(S_0) can be excited to any of the excited triplet states having a smaller singlet-triplet gap than the lowest singlet-triplet gap of the photosensitizer. From Supplementary Fig. 18 it is obvious that D can be excited up to T_5 excited states by PS1. Therefore, the HAT transition state is ~ 9 kcal/mol above from the T_5 excited state (Fig. 4 in the manuscript). Interestingly, other photocatalysts, PS2, PS3, and PS4, having smaller singlet-triplet energy gaps, are not capable to excite D to an excited state higher than the T_4 state, which corresponds to the electron promoted to the π* of bpy ligand, not to the σ* Ni-Br bond, which is not preferred for active bromine atom generation. Moreover, the HAT step from an excited state lower than T_5 would also be kinetically slow, because a very high free energy barrier (~25 kcal/mol) would then be required to reach the transition state for the HAT step. Therefore, following the experimental findings, PS2, PS3, and PS4 photocatalysts are not effective for the C(sp^3)–H alkylation reaction.

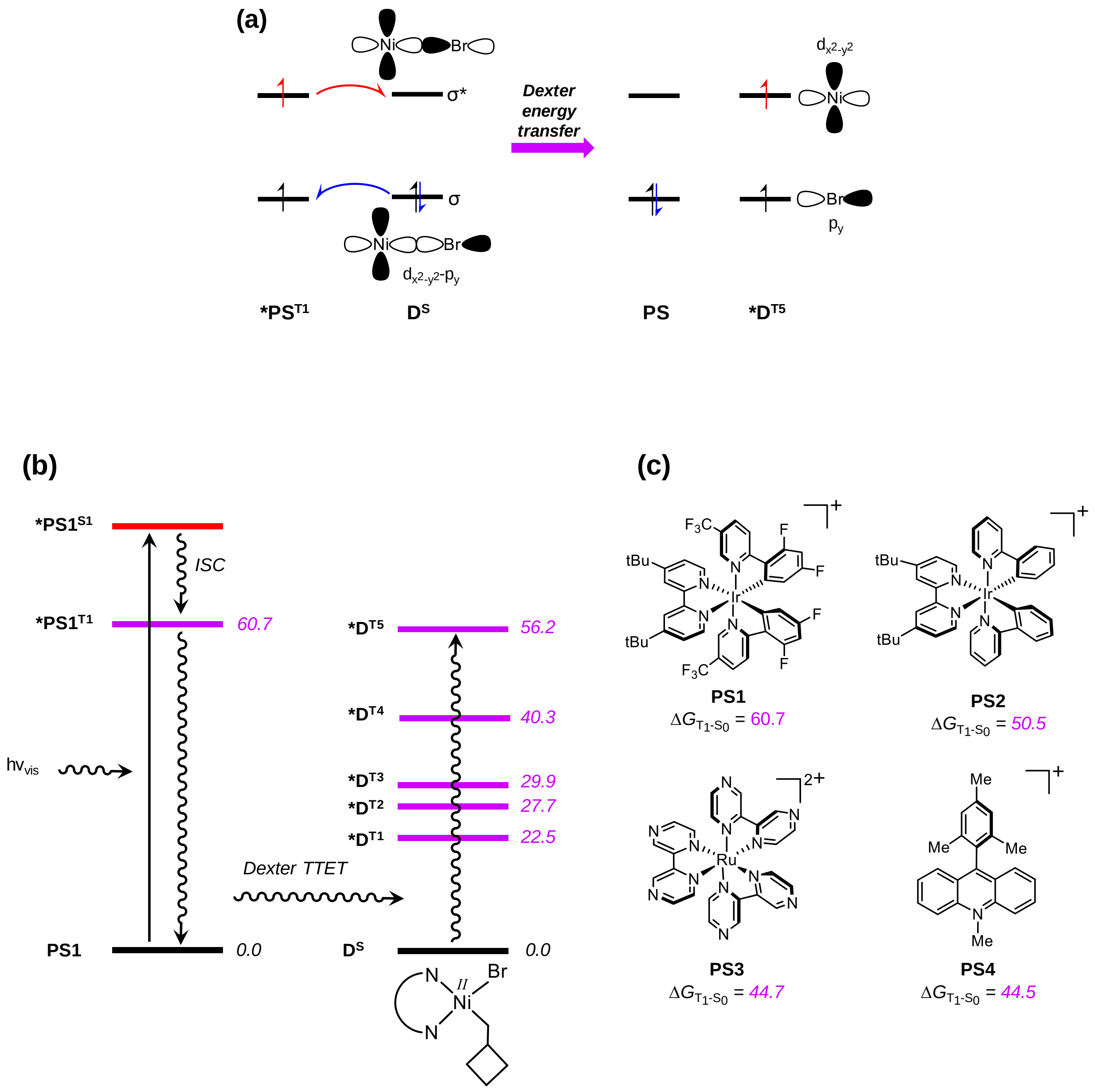

Supplementary Figure 18. a-b Dexter triplet-triplet energy transfer. PS: photocatalyst, D^s: Ni(II)(alkyl)Br (quencher), ISC: intersystem crossing, S_0: singlet ground state, S_1: first singlet excited state, T_n: n^{th} triplet excited state. **c** The chemical structures of four photocatalysts tested for the reaction. Energy values for photocatalyst are reported from DFT calculations and for intermediate D^s, all linear excitation energies at different triplet states are from TD-DFT calculations. All energy values are in kcal/mol. Energies are in kcal/mol.

2. Energy profile for the electron transfer pathway of reductive elimination step

For energy and other conventions refer to Fig. 4 in the manuscript.

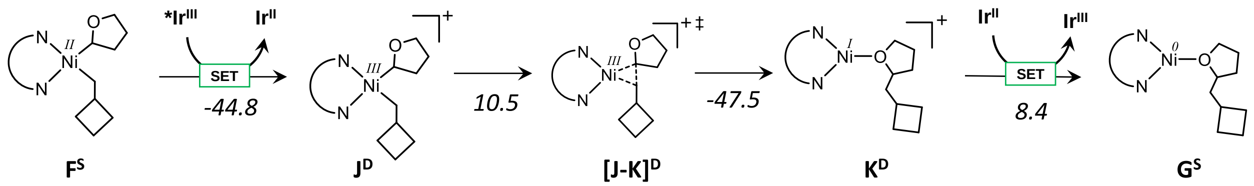

3. Alternative mechanism involving NiI/III intermediate

An alternative mechanism involving Ni(I/III) is also considered to study. In this mechanism, the D^S intermediate gets reduced to generate L^D, a step endergonic by 18.1 kcal/mol. Oxidative addition of 1_R on the L^D requires the energy barrier of 29.4 kcal/mol, generating M^D. From M^D the C_{sp3}–H bond activation is highly unfavorable because of the very high activation barrier of 69.7 kcal/mol. On the other have the intermediate M^D has a better chance to undergo reductive elimination to form the alkyl-alkyl cross-coupling product. But this product is not observed in the experiment. Therefore, these results indirectly predict that the single electron reduction of D^S is unfavored, and hence the Ni(I/III) mechanism can be excluded.

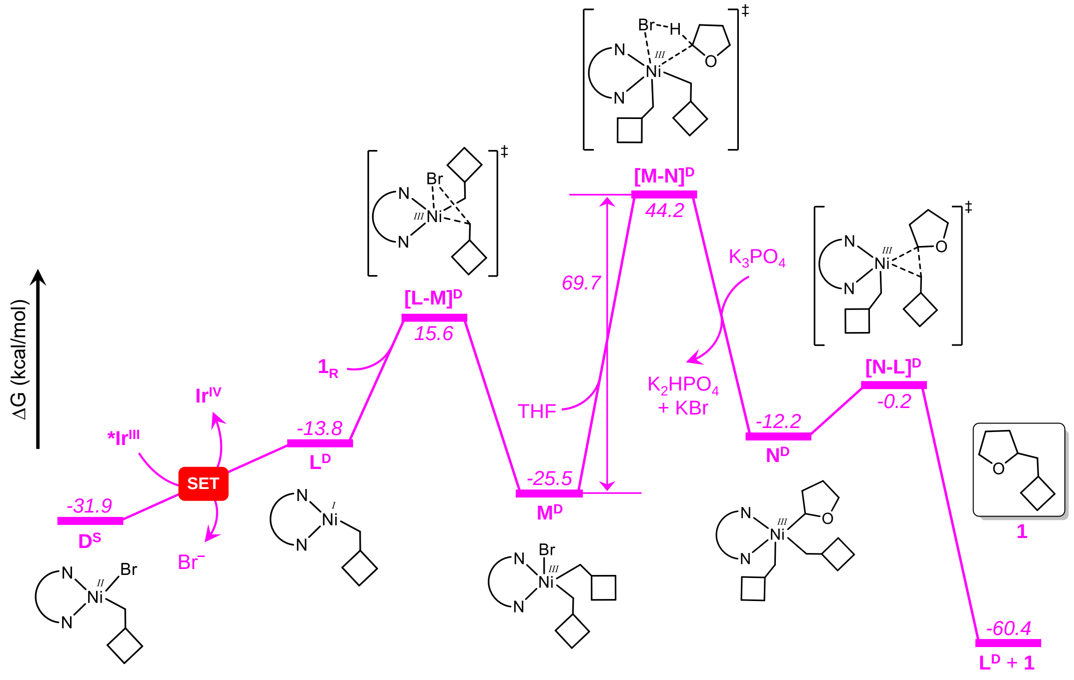

Supplementary Figure 19. Energy profile for the alternative pathway involving Ni(I/III) catalytic cycle. For energy and other conventions refer to Fig. 4 in the manuscript.

4. PES (potential energy surface) and free energy of C_α−H bond activation of THF by active Br atom

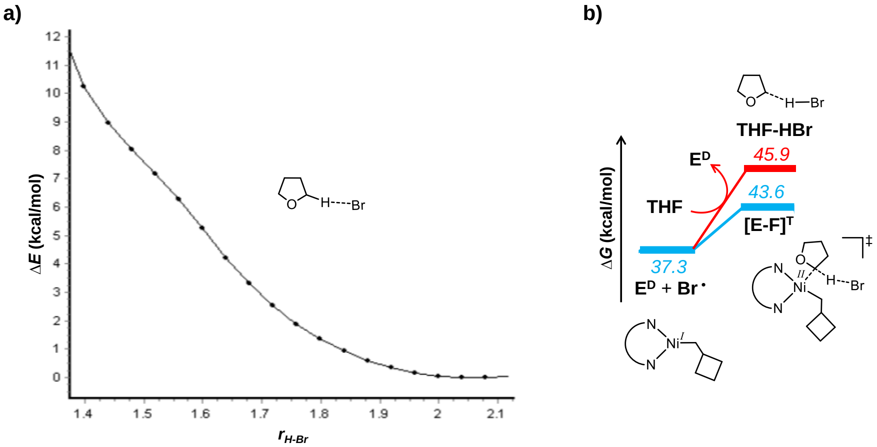

Supplementary Figure 20. a PES (potential energy surface), and **b** free energy of C_α−H bond activation of THF by active Br atom.

In an alternative pathway, the outer-sphere mechanism of C_α−H activation by active Br atom has been considered. In the PES (potential energy surface) energy is gradually increasing along the coordinate of H and Br bond formation (Supplementary Fig. 20). We have selected one structure for single-point energy calculation from the PES where the H−Br bond is already formed. That structure is unstable by 2.3 kcal/mol than the inner sphere transition state $[E\text{-}F]^T$. Finally, the outer sphere mechanism by the active Br atom is less favored than the inner-sphere mechanism.

5. Discussion regarding the triplet states of nickel

The triplet states of Ni(0) and Ni(II) reported in this study are ^{3}d-d types, which is in agreement with the recent report.[22] Although a very low singlet-triplet energy gap of 4-5 kcal/mol is found for both Ni(0) and Ni(II) bipyridine complexes (A^S and D^S), the ^{3}d-d type of triplet is thermally inaccessible from the ground state. Photoexcitation of ground state singlet leads to the ^{1}MLCT type excited state, which eventually gives ^{3}MLCT type triplet. The ^{3}MLCT is a higher energy state, which eventually undergoes IC (internal conversion) to the most stable ^{3}d-d state.[22] However, in the current alkylation reaction the ^{3}d-d state is unproductive.

In TDDFT calculations we desired to have electronic information of higher energy triplet states. The low lying triplets (T1-T4) in the TDDFT calculations are ^{3}MLCT types (vertical excitation), whereas the D^T is ^{3}d-d type (optimized geometry). Therefore the energy of D^T is not comparable with the TDDFT calculated triplet state.

Moreover, ^{3}MLCT T1−T4 states are not proficient for C(sp^3)–H activation via Ni−Br homolysis. On the other hand T_5 state correspond to electron population at Ni−Br σ* bond, which is mostly contributed by Ni-$d_{x^2-y^2}$ orbital. Therefore T_5 state is characterized as a ^{3}d-d type state which readily undergoes Ni−Br homolysis, and there will be minor chance of IC (internal conversion) from T_5 to lower energy T1−T4 states. Of course, the Dexter EnT transfer can occur from *PS1 to any of the first 5 triplet excited states of D^S, Tn, n=1-5, with only that leading to T5 being effective in catalysis. And we cannot consider that the population of T1−T4 states are predicted as deactivation pathway (since the catalyst is not becoming inactive), they will convert to D^S via ISC (inter system crossing).

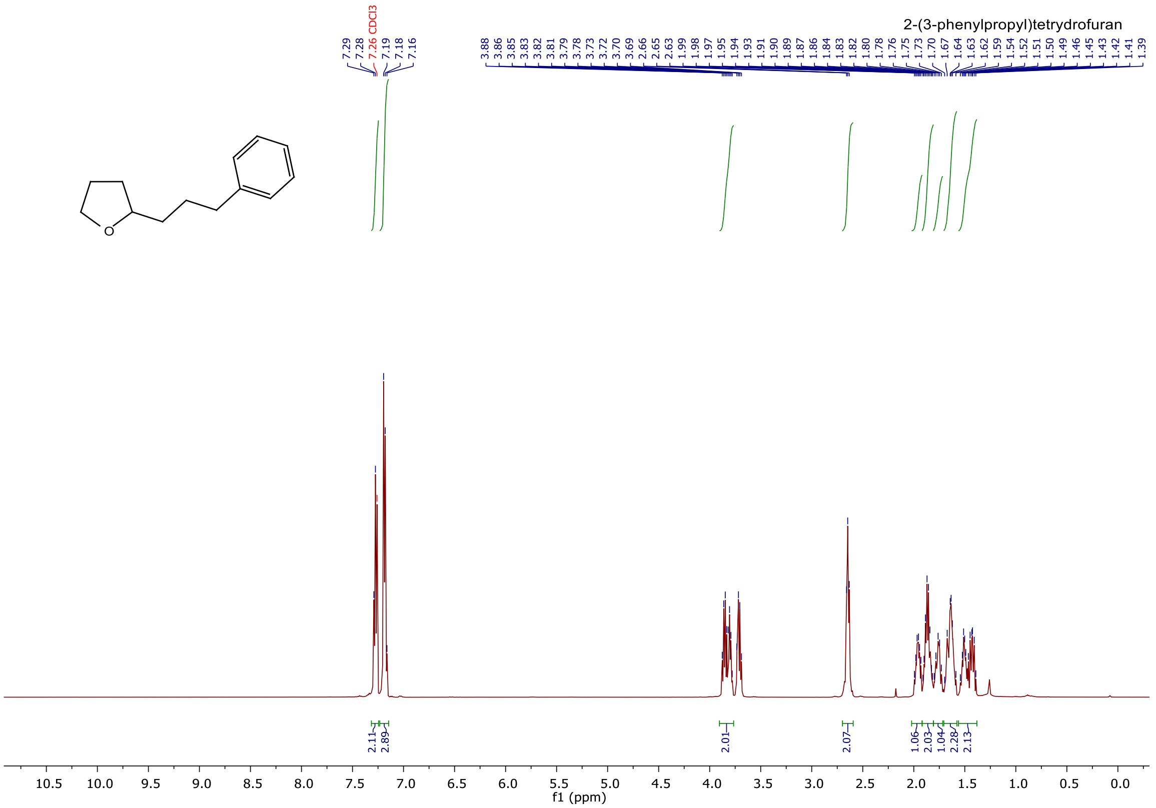

Supplementary Figure 21. ^{1}H NMR (500 MHz, $CDCl_3$) of compound **1**

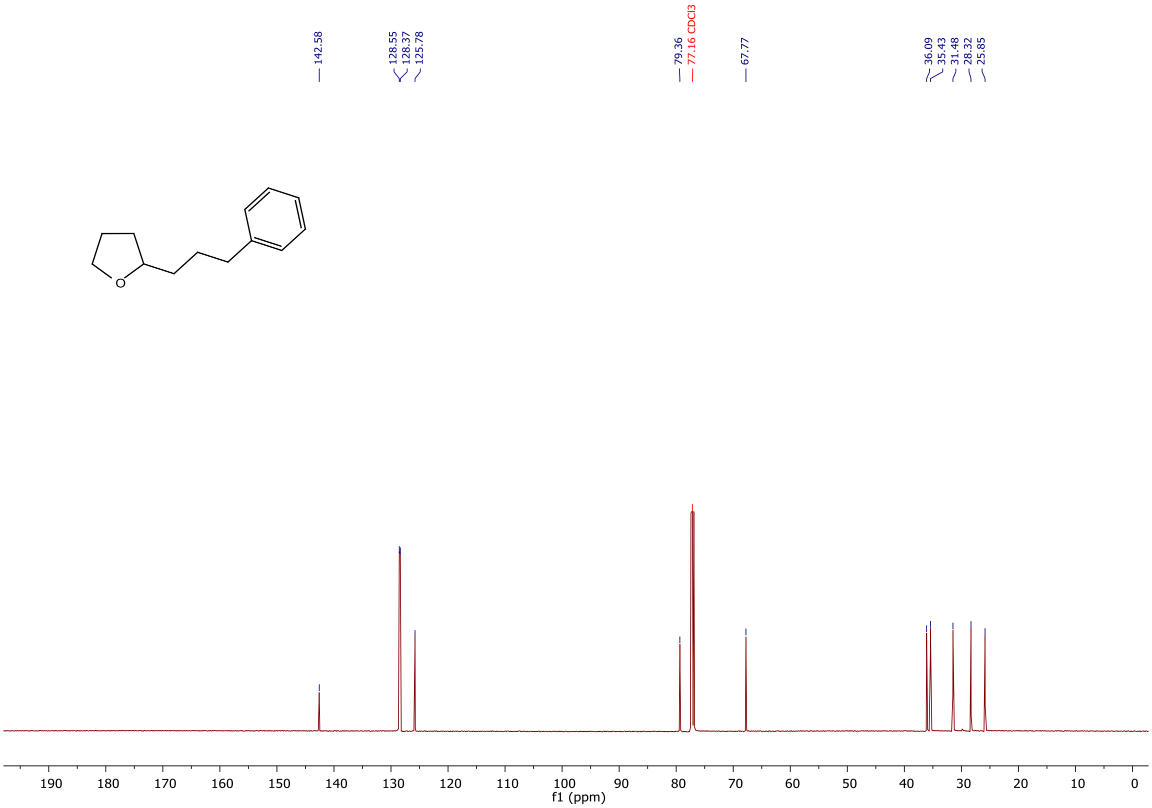

Supplementary Figure 22. ^{13}C NMR (126 MHz, $CDCl_3$) of compound **1**

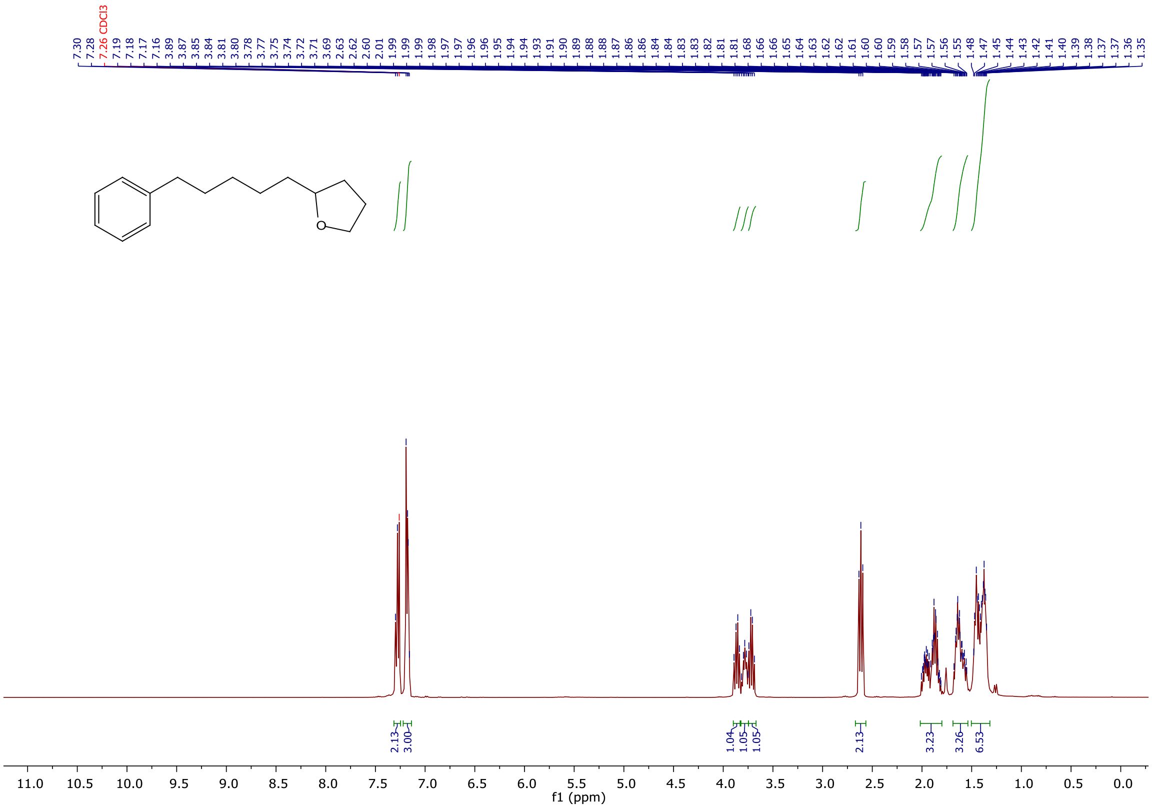

Supplementary Figure 23. ^{1}H NMR (400 MHz, $CDCl_3$) of compound **2**

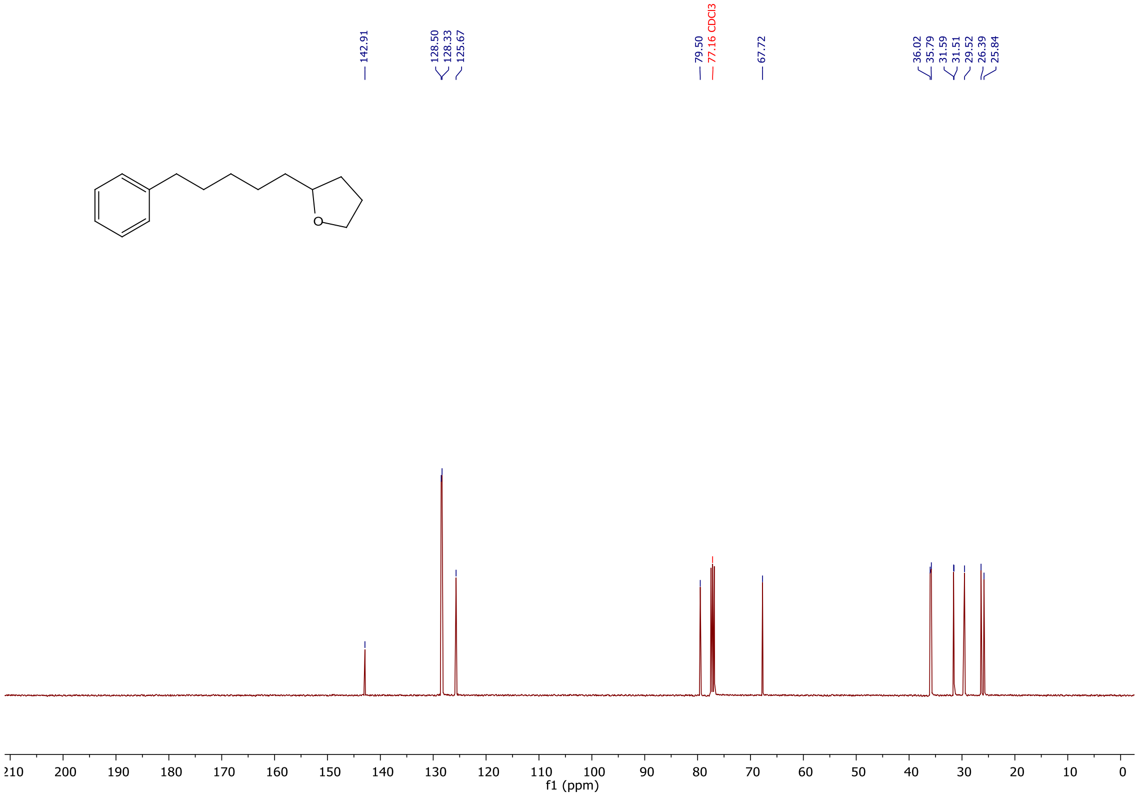

Supplementary Figure 24. ^{13}C NMR (101 MHz, $CDCl_3$) of compound **2**

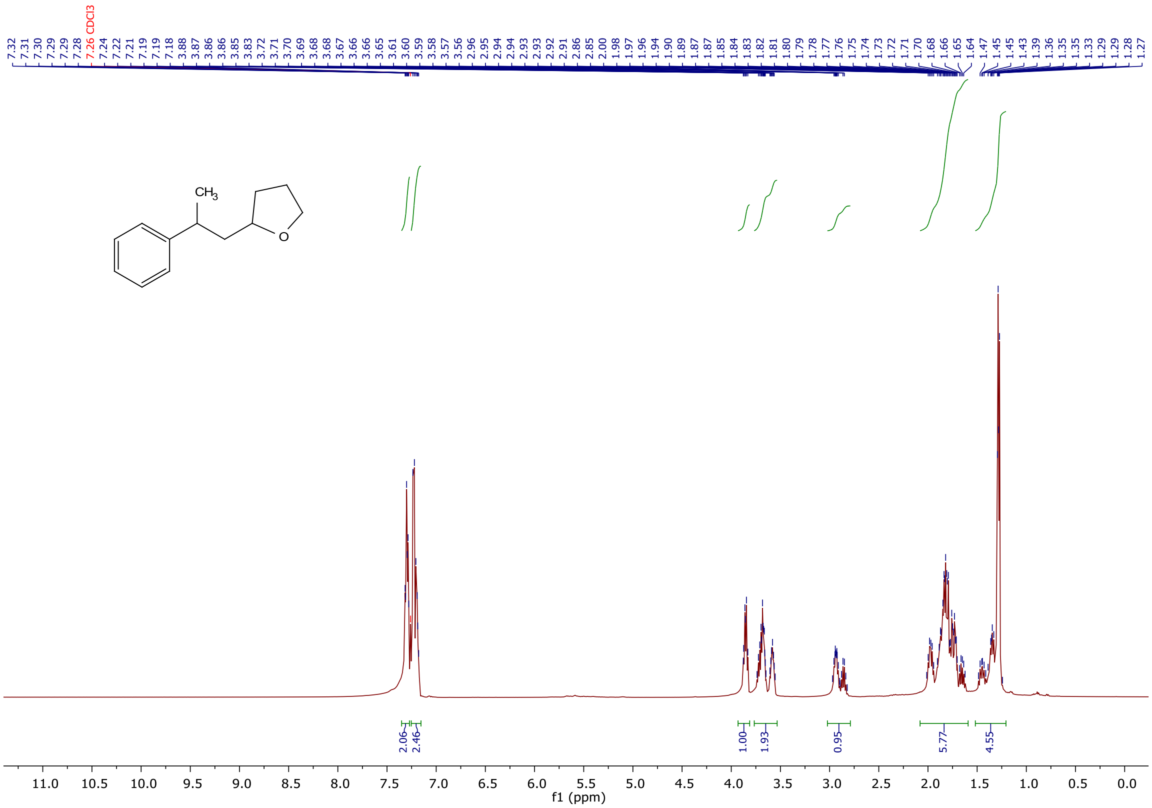

Supplementary Figure 25. ^{1}H NMR (500 MHz, $CDCl_3$) of compound **3**

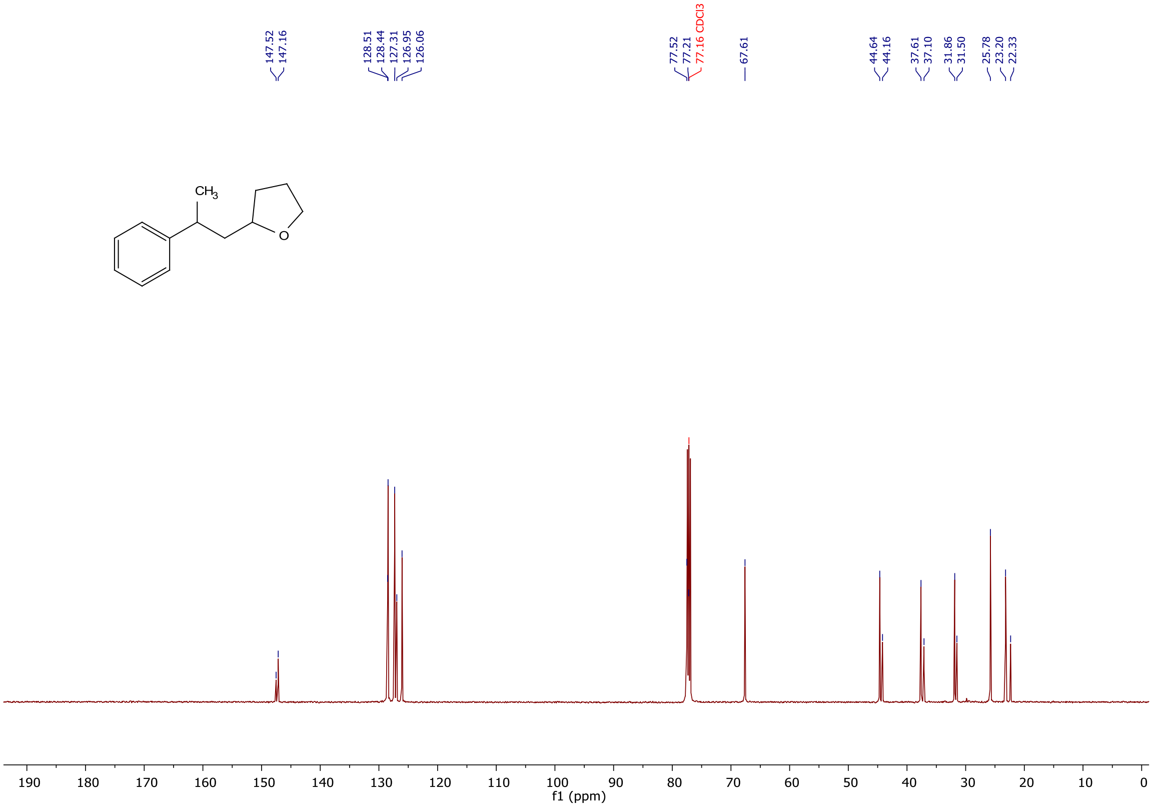

Supplementary Figure 26. ^{13}C NMR (126 MHz, $CDCl_3$) of compound **3**

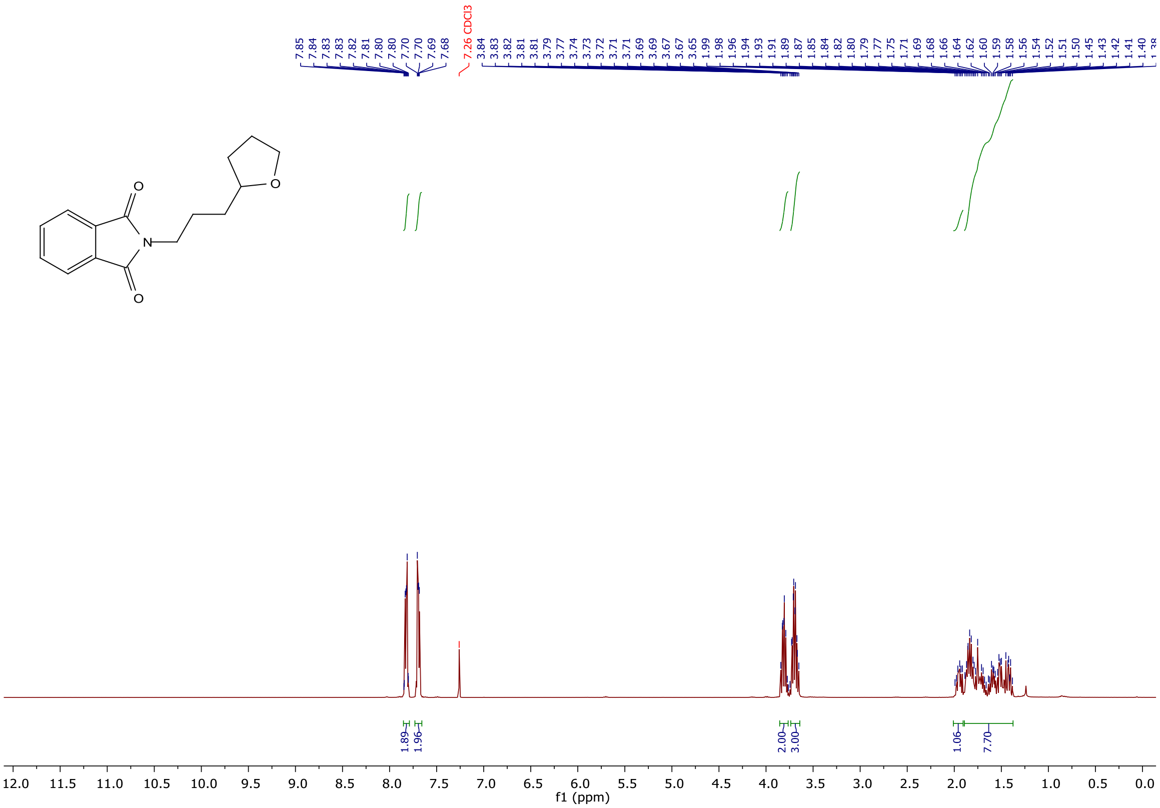

Supplementary Figure 27. ^{1}H NMR (400 MHz, $CDCl_3$) of compound **4**

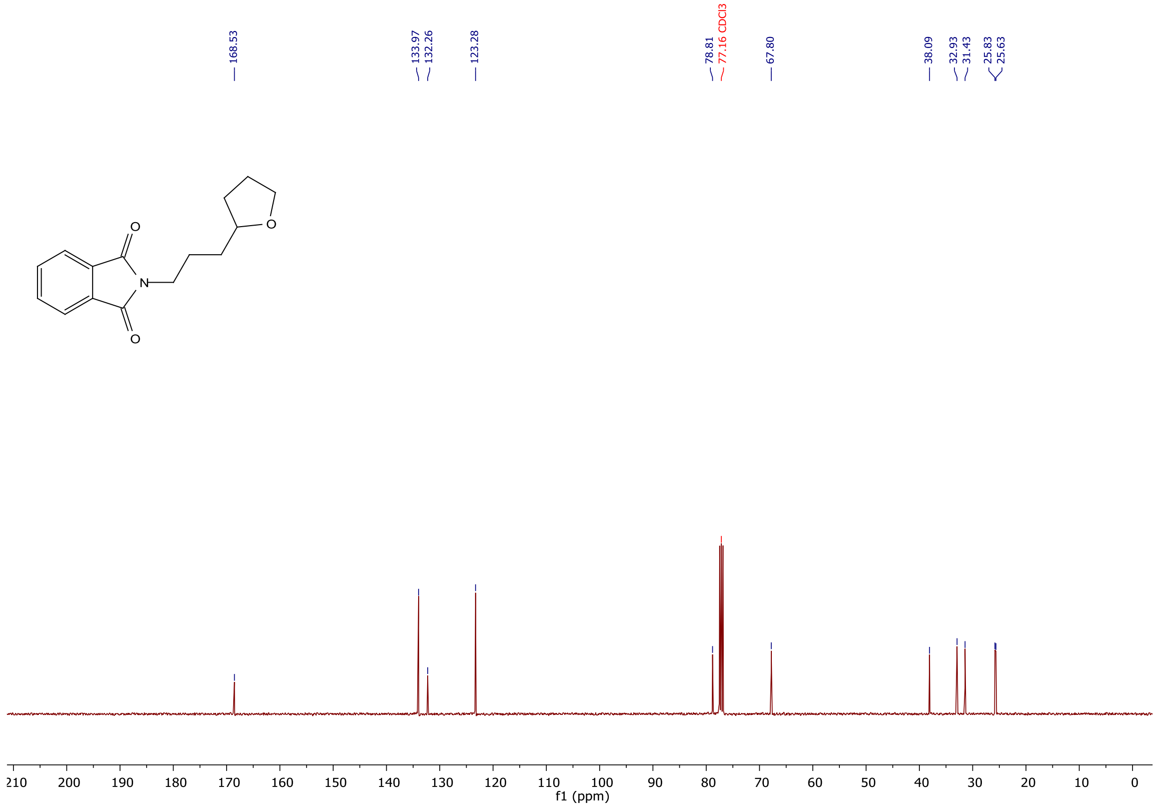

Supplementary Figure 28. ^{13}C NMR (101 MHz, $CDCl_3$) of compound **4**

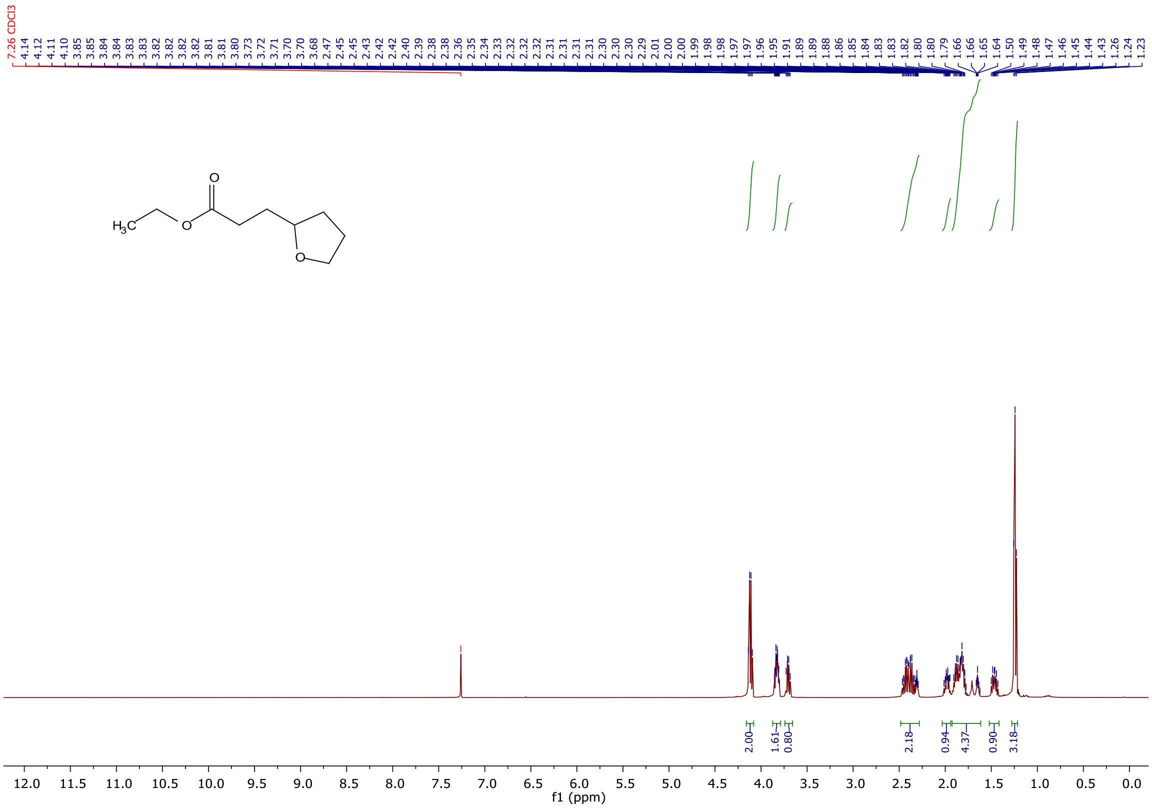

Supplementary Figure 29. ^{1}H NMR (500 MHz, $CDCl_3$) of compound **5**

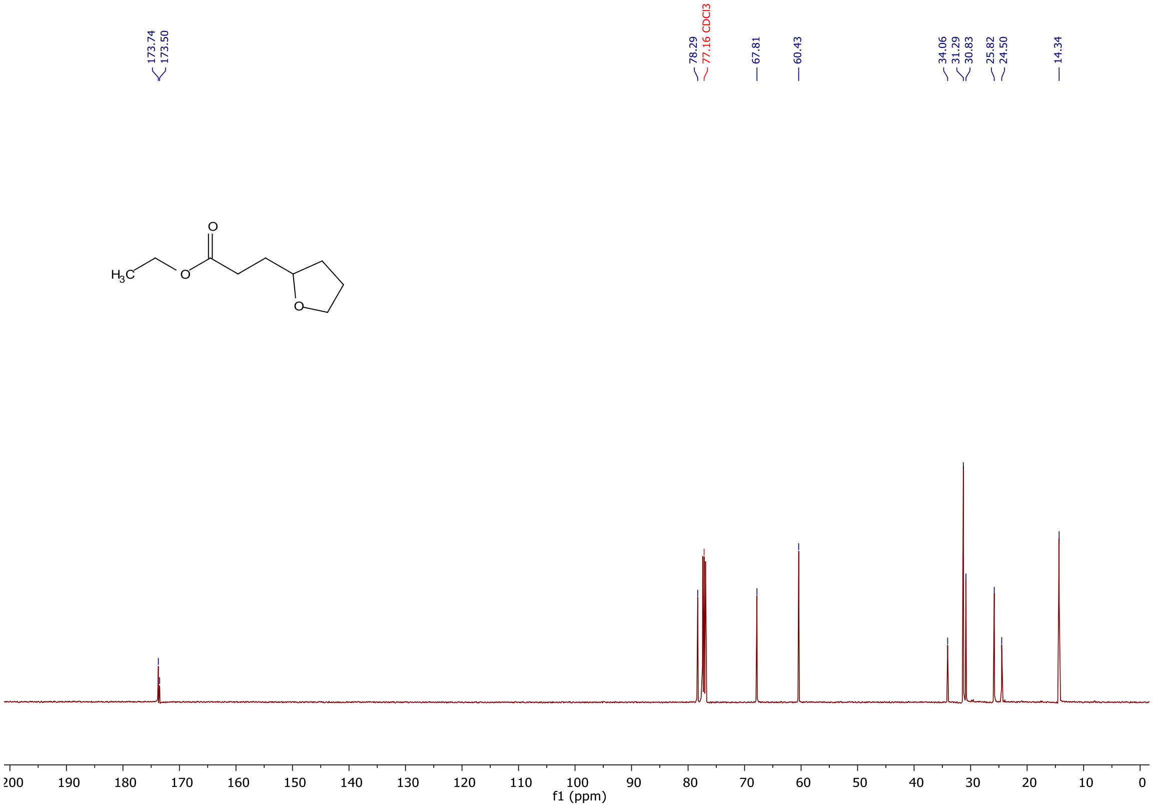

Supplementary Figure 30. ^{13}C NMR (126 MHz, $CDCl_3$) of compound **5**

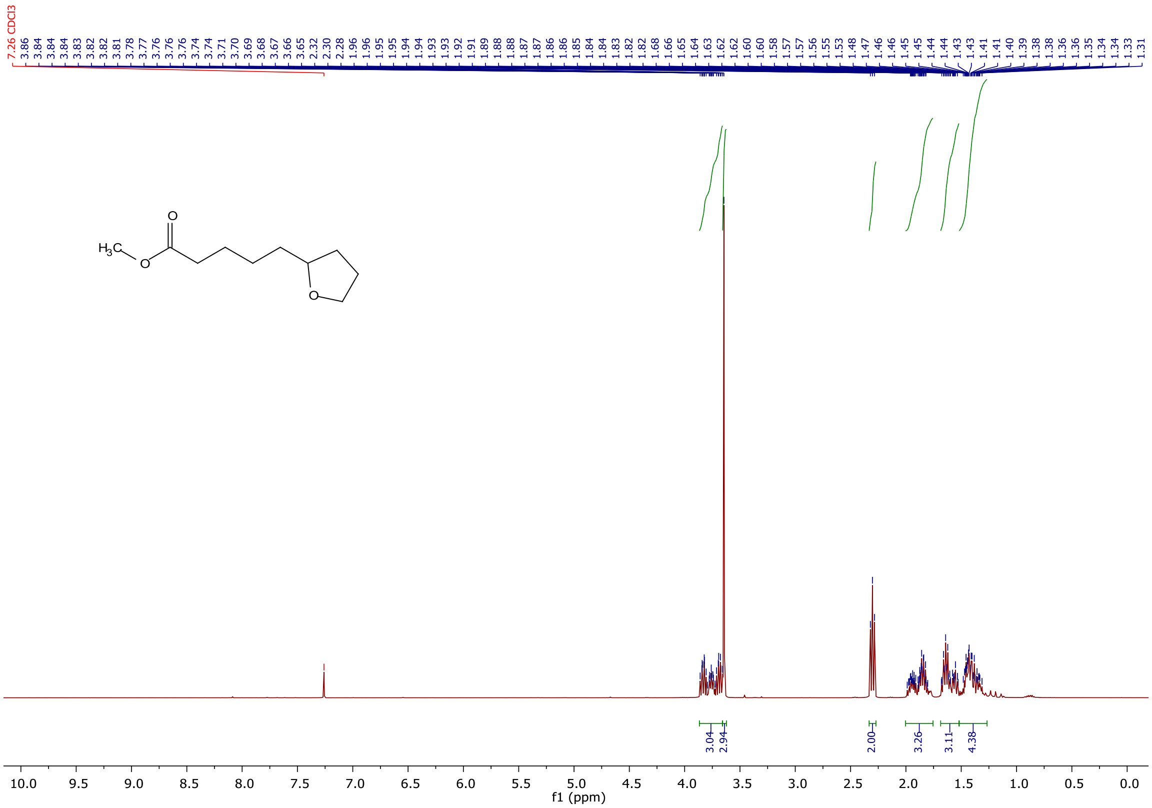

Supplementary Figure 31. ^{1}H NMR (400 MHz, $CDCl_3$) of compound **6**

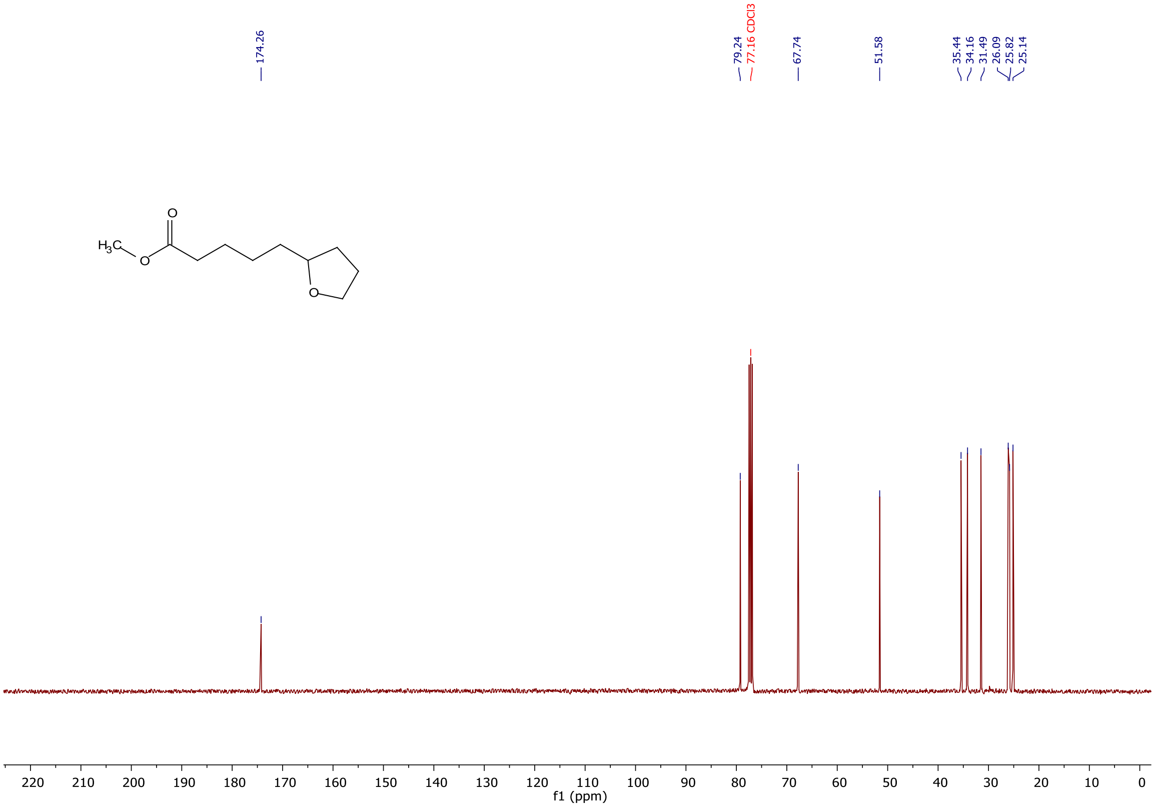

Supplementary Figure 32. ^{13}C NMR (101 MHz, $CDCl_3$) of compound **6**

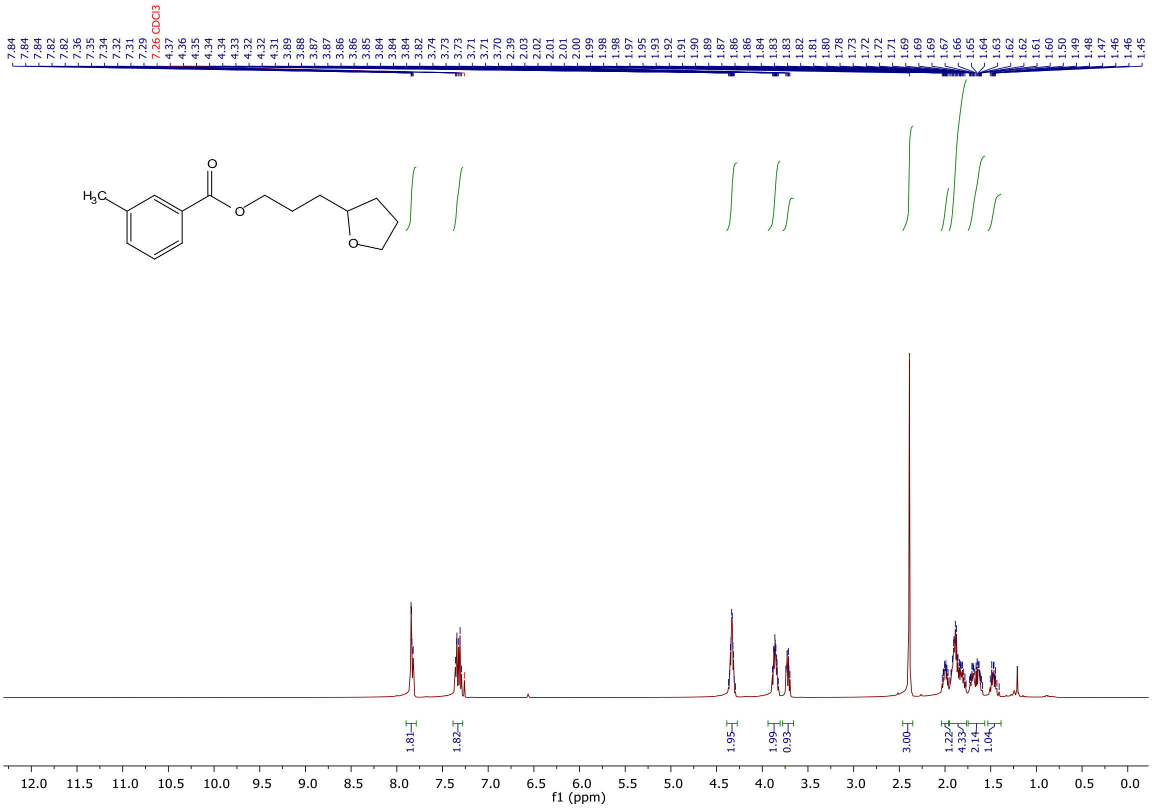

Supplementary Figure 33. ^{1}H NMR (500 MHz, $CDCl_3$) of compound **7**

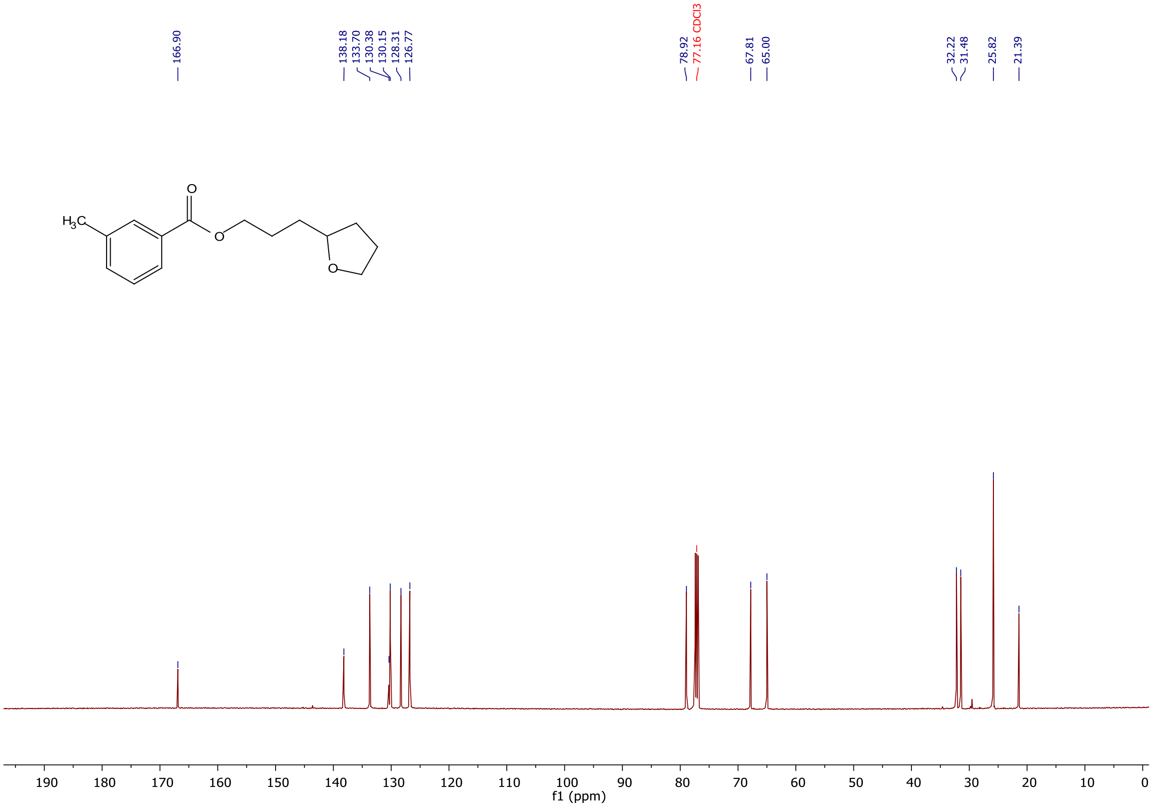

Supplementary Figure 34. ^{13}C NMR (126 MHz, $CDCl_3$) of compound **7**

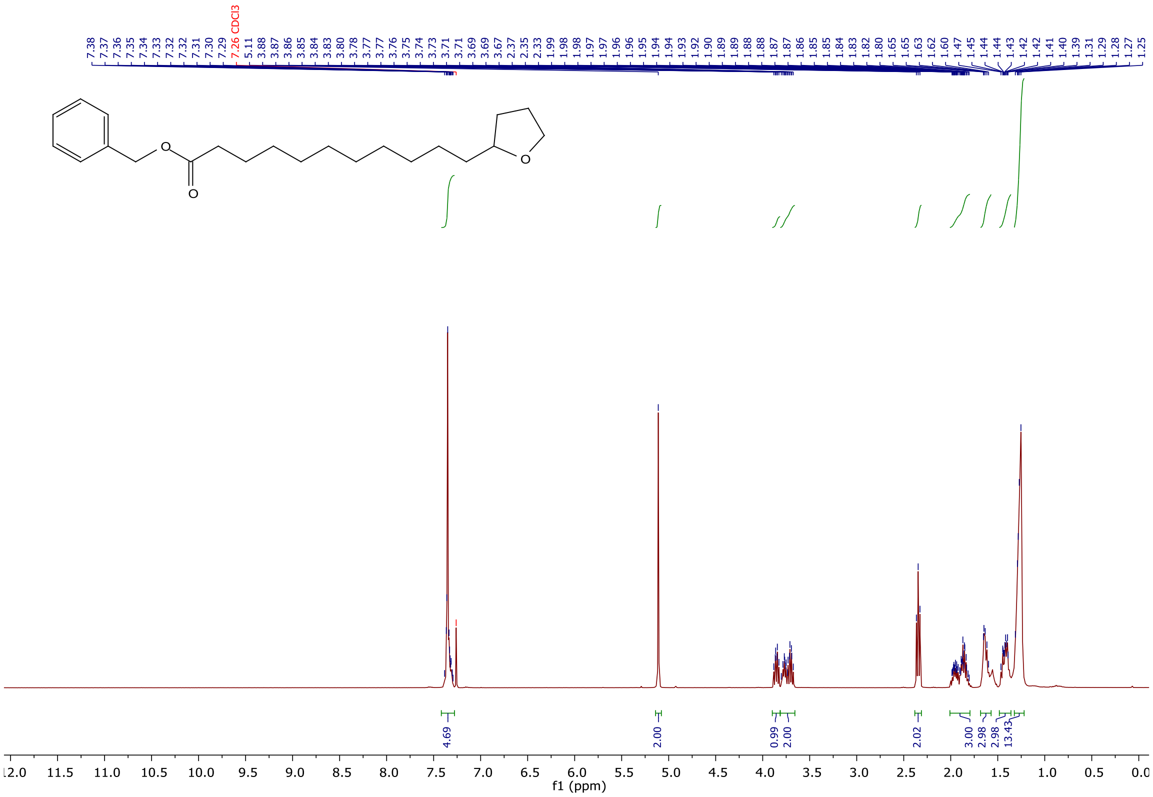

Supplementary Figure 35. ^{1}H NMR (400 MHz, $CDCl_3$) of compound **8**

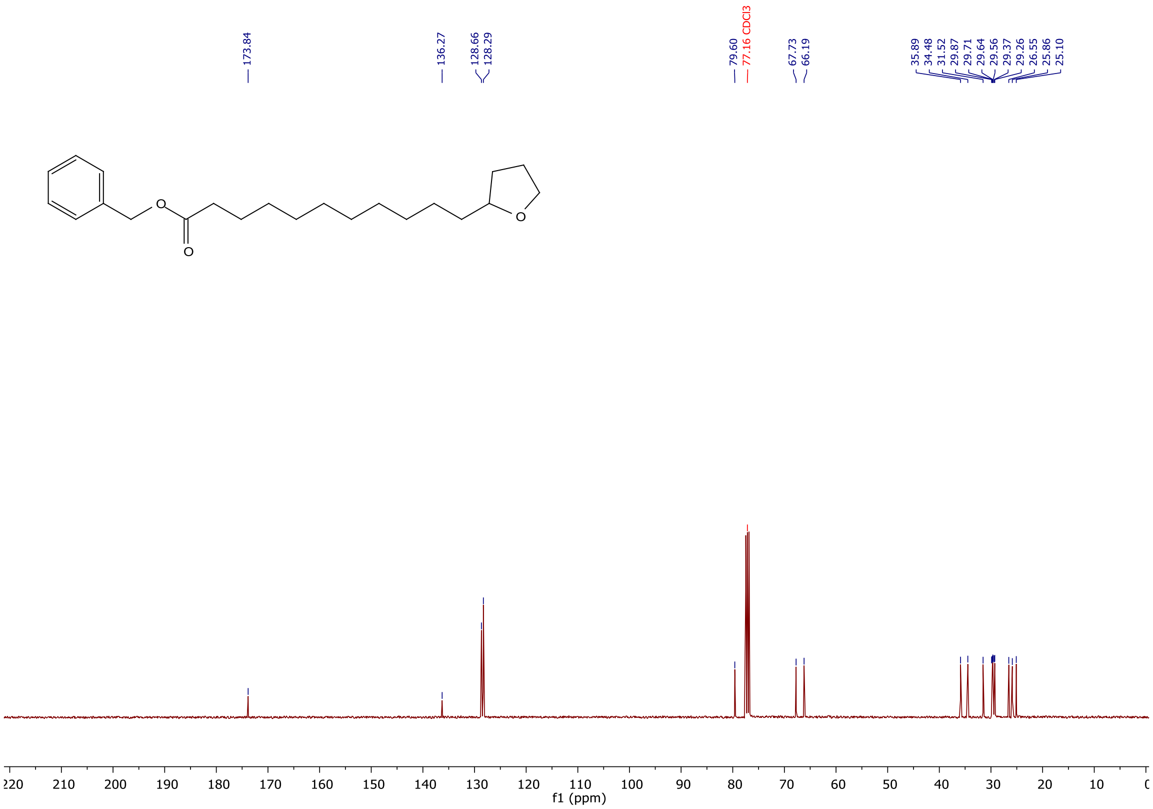

Supplementary Figure 36. ^{13}C NMR (101 MHz, $CDCl_3$) of compound **8**

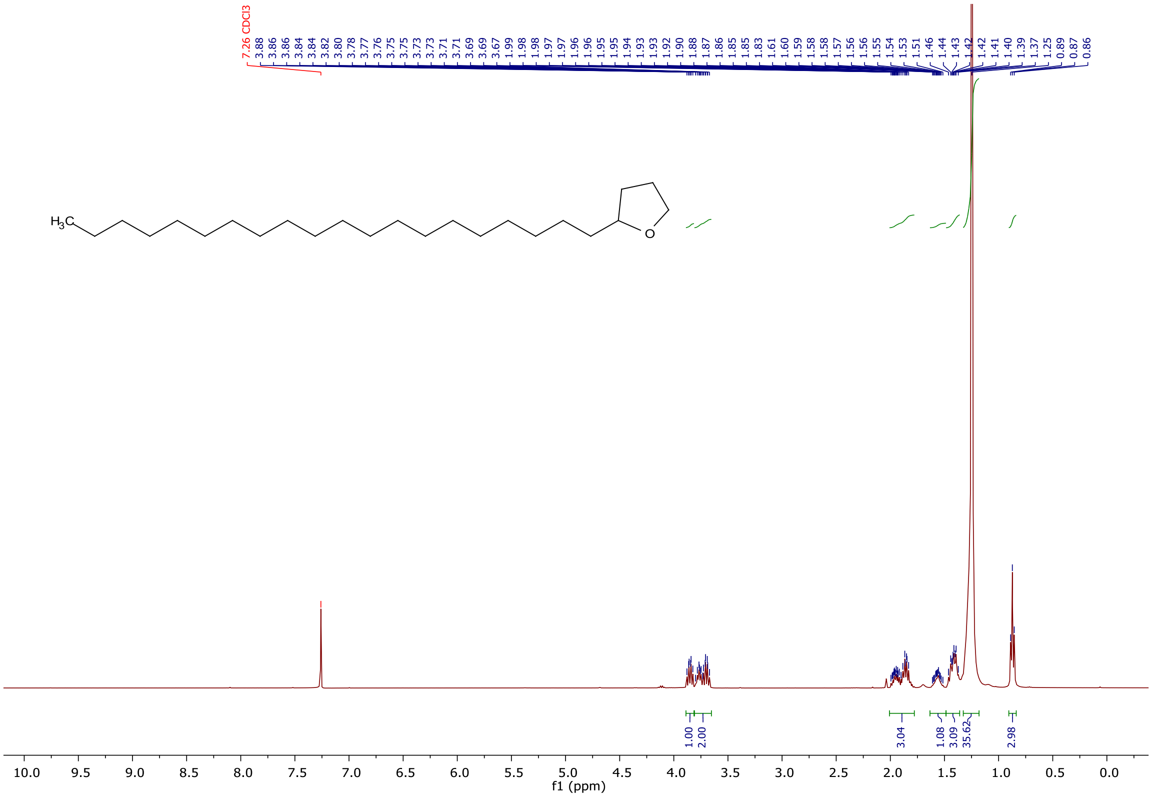

Supplementary Figure 37. ^{1}H NMR (400 MHz, $CDCl_3$) of compound **9**

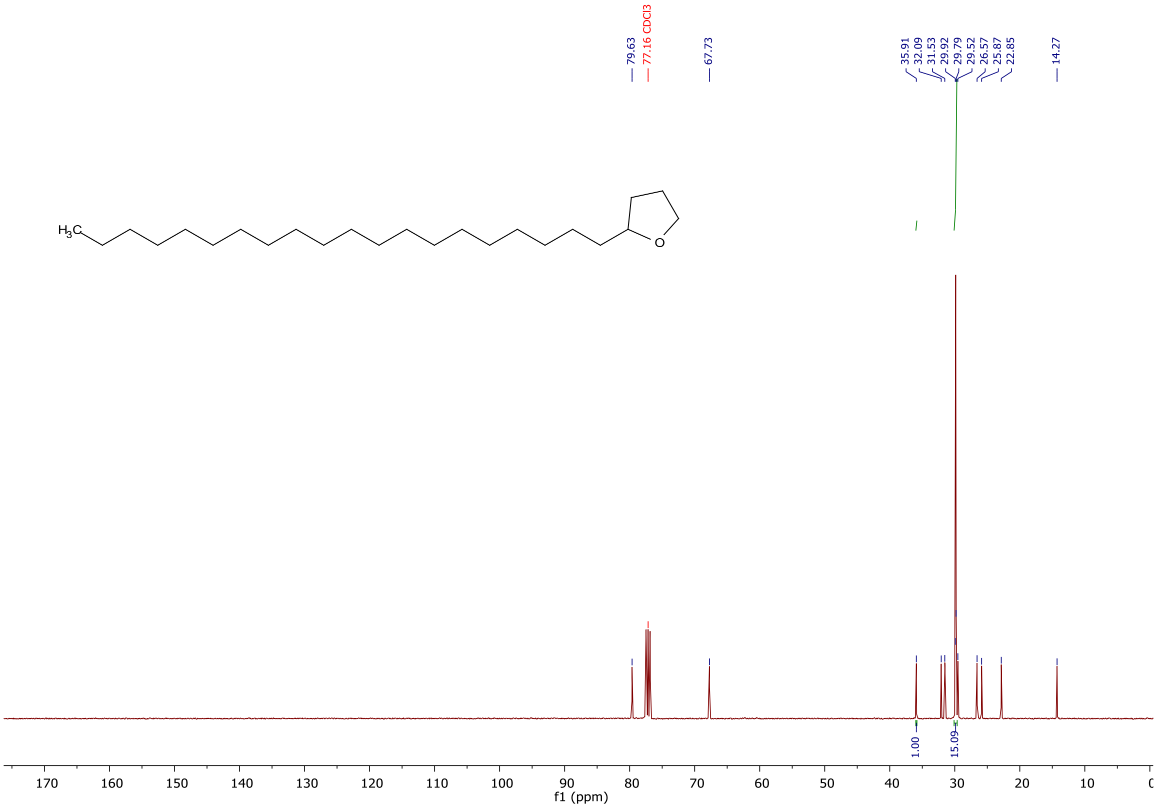

Supplementary Figure 38. ^{13}C NMR (101 MHz, $CDCl_3$) of compound **9**

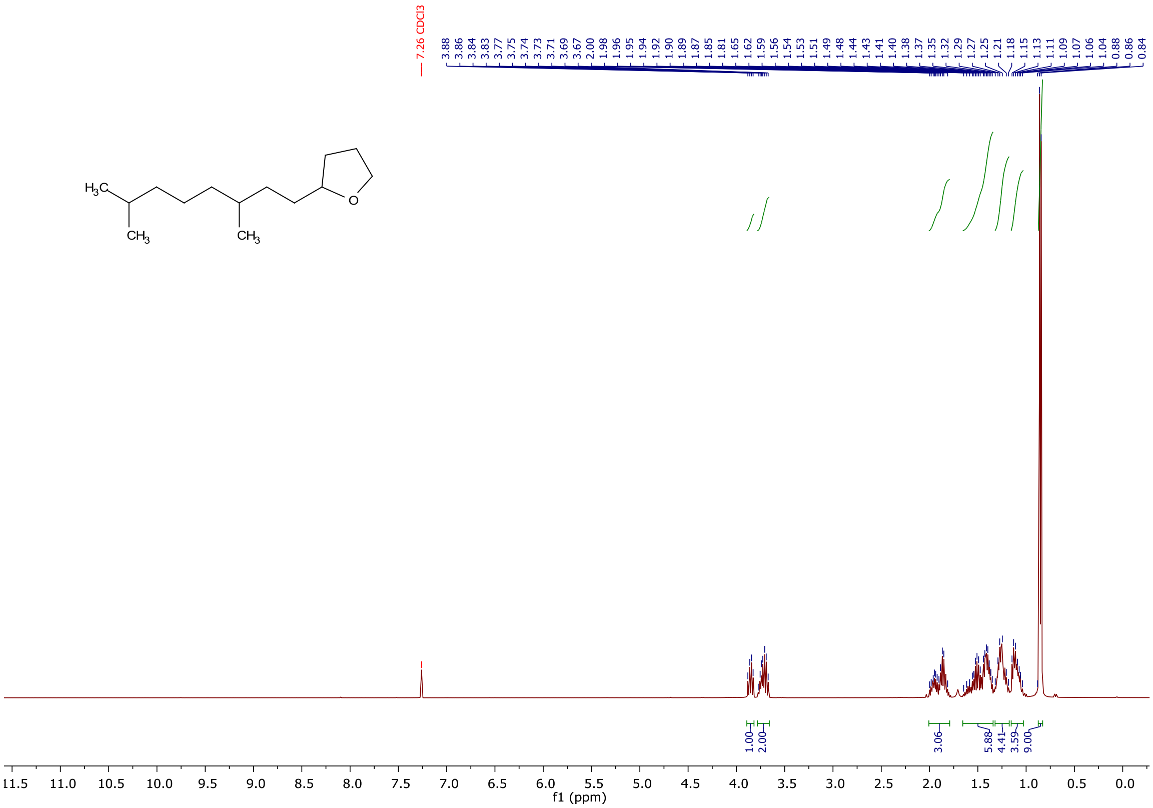

Supplementary Figure 39. ^{1}H NMR (400 MHz, $CDCl_3$) of compound **10**

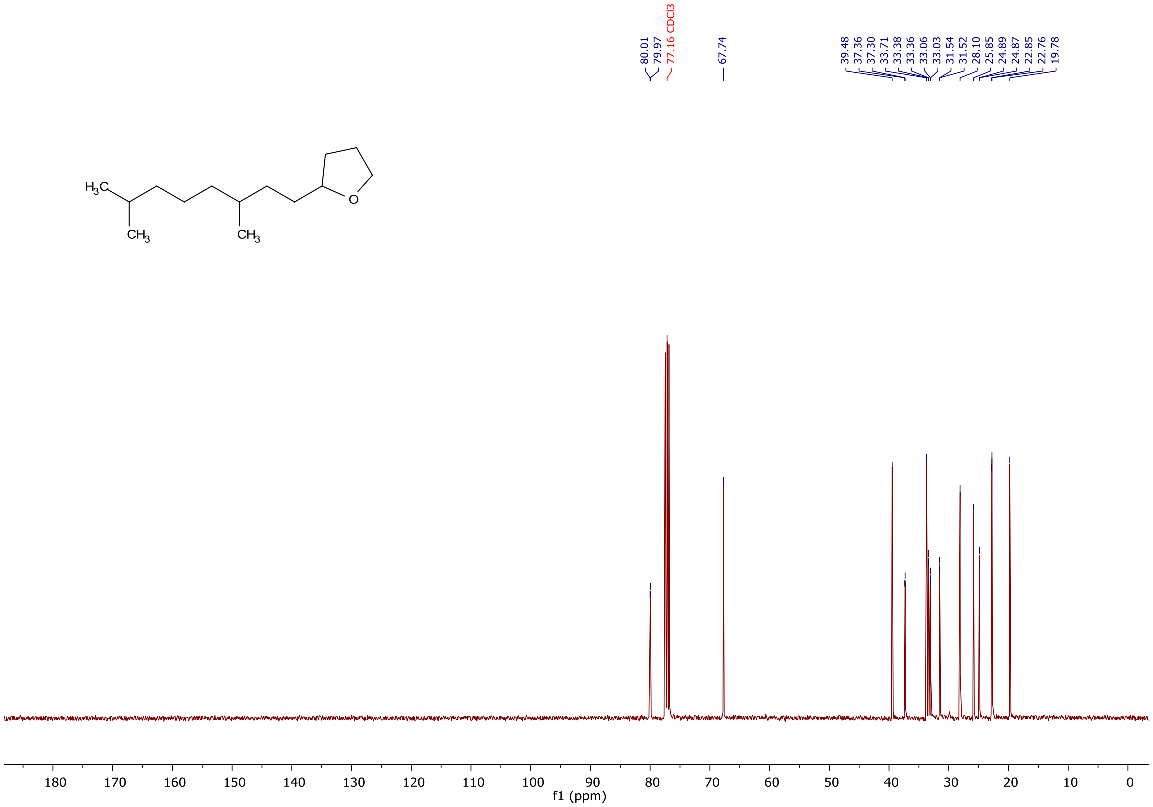

Supplementary Figure 40. ^{13}C NMR (101 MHz, $CDCl_3$) of compound **10**

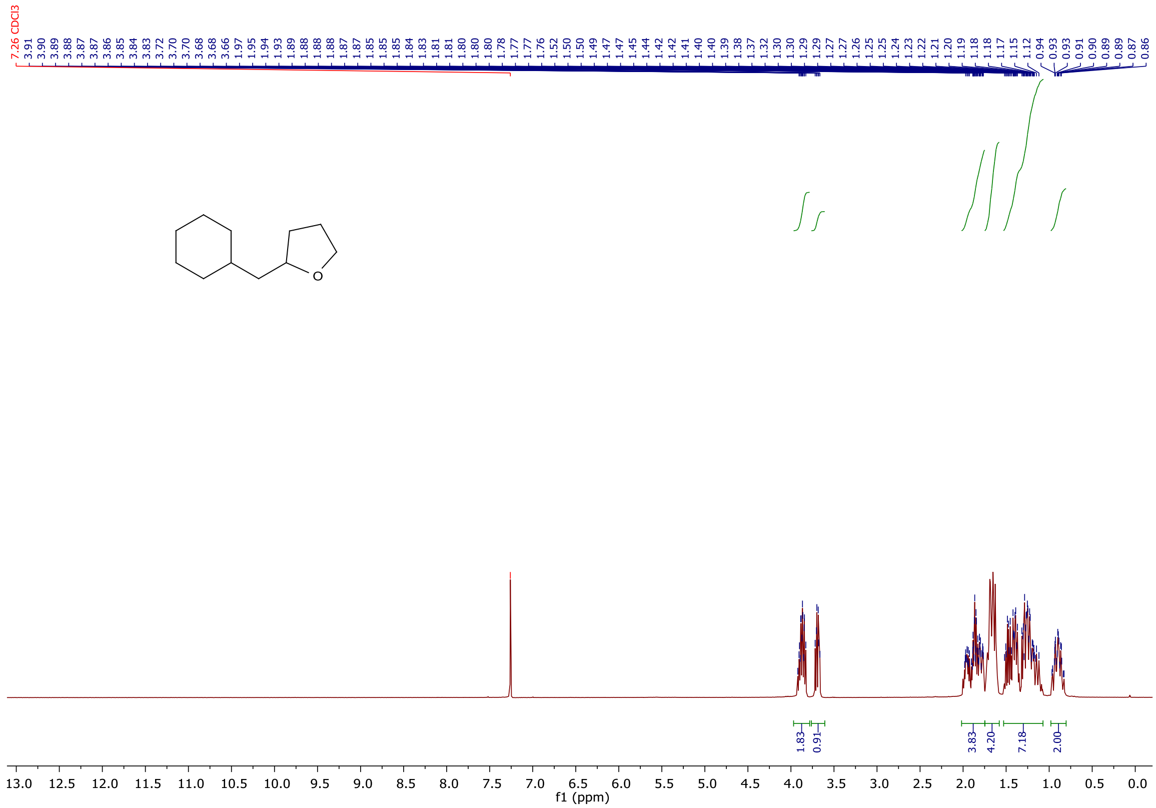

Supplementary Figure 41. ^{1}H NMR (400 MHz, $CDCl_3$) of compound **11**

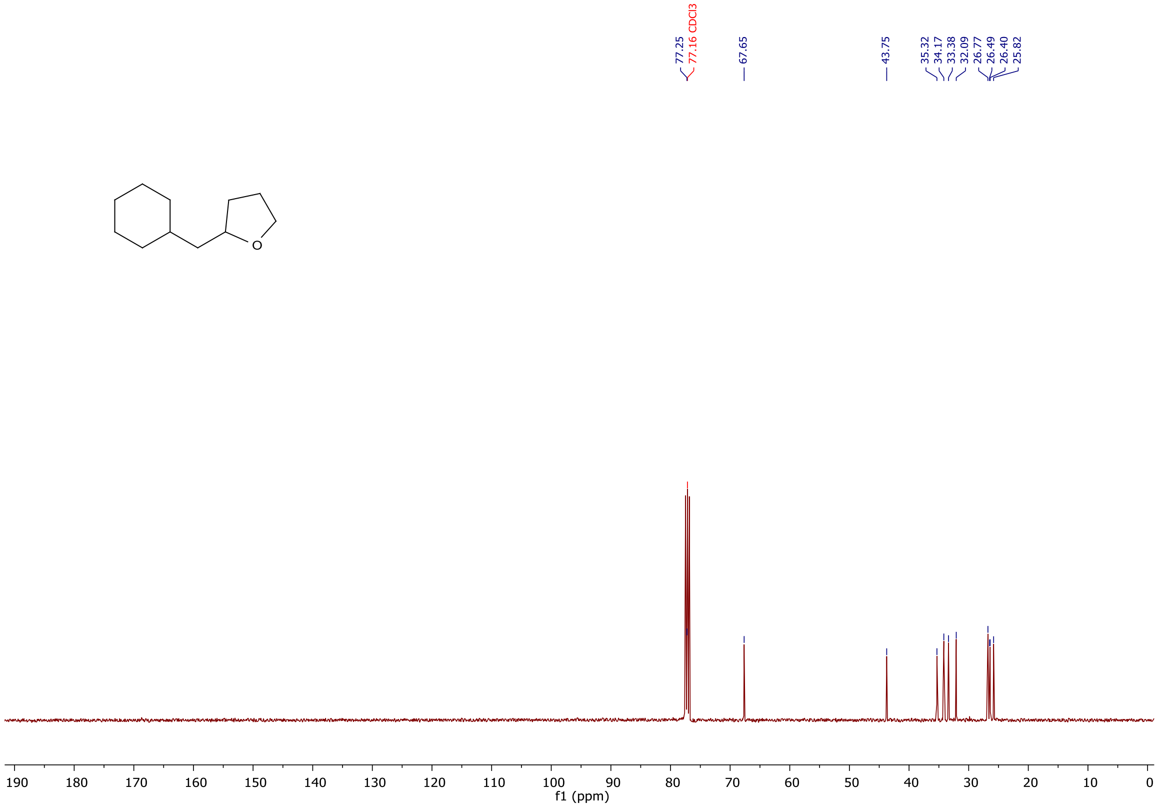

Supplementary Figure 42. ^{13}C NMR (101 MHz, $CDCl_3$) of compound **11**

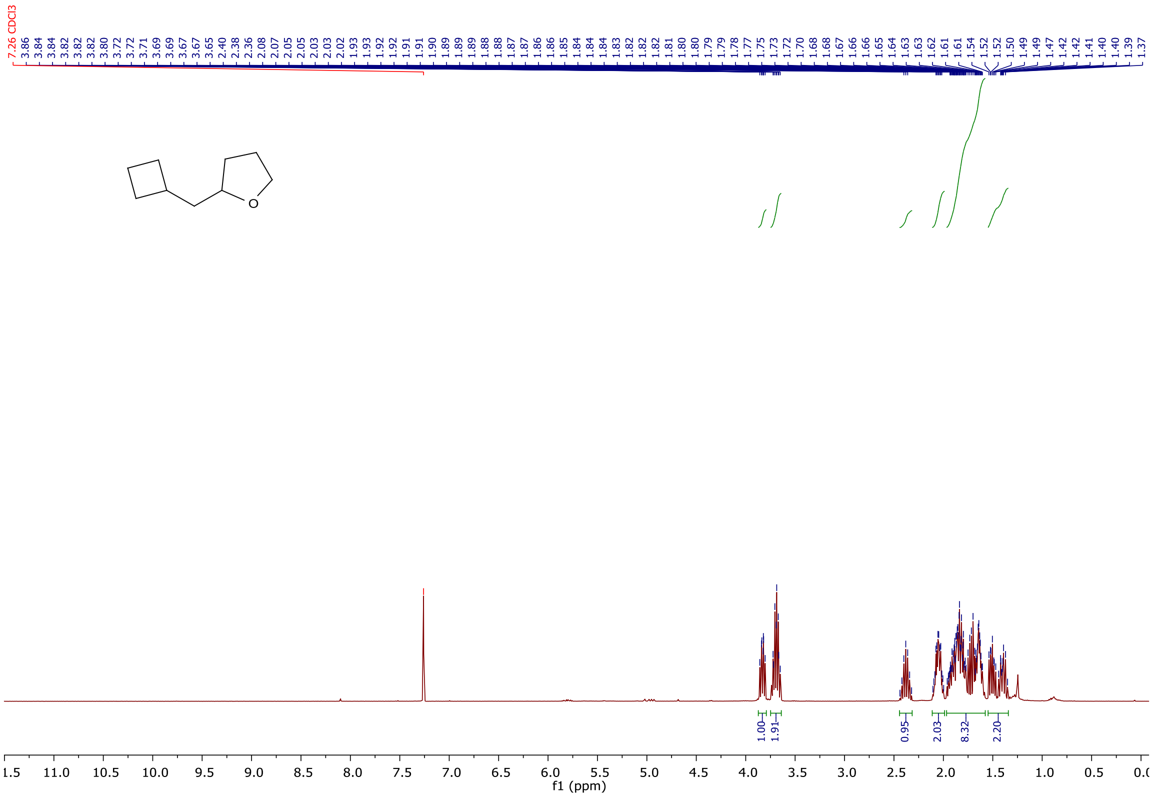

Supplementary Figure 43. ^{1}H NMR (400 MHz, $CDCl_3$) of compound **12**

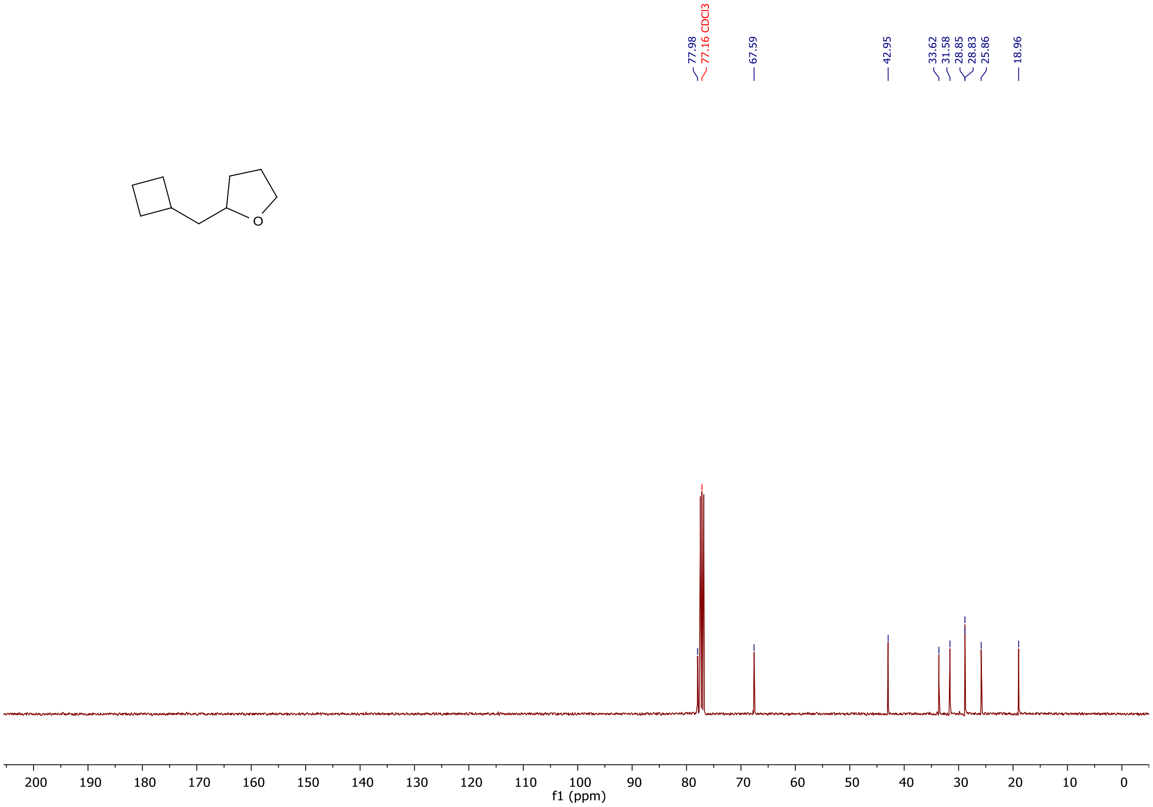

Supplementary Figure 44. ^{13}C NMR (101 MHz, $CDCl_3$) of compound **12**

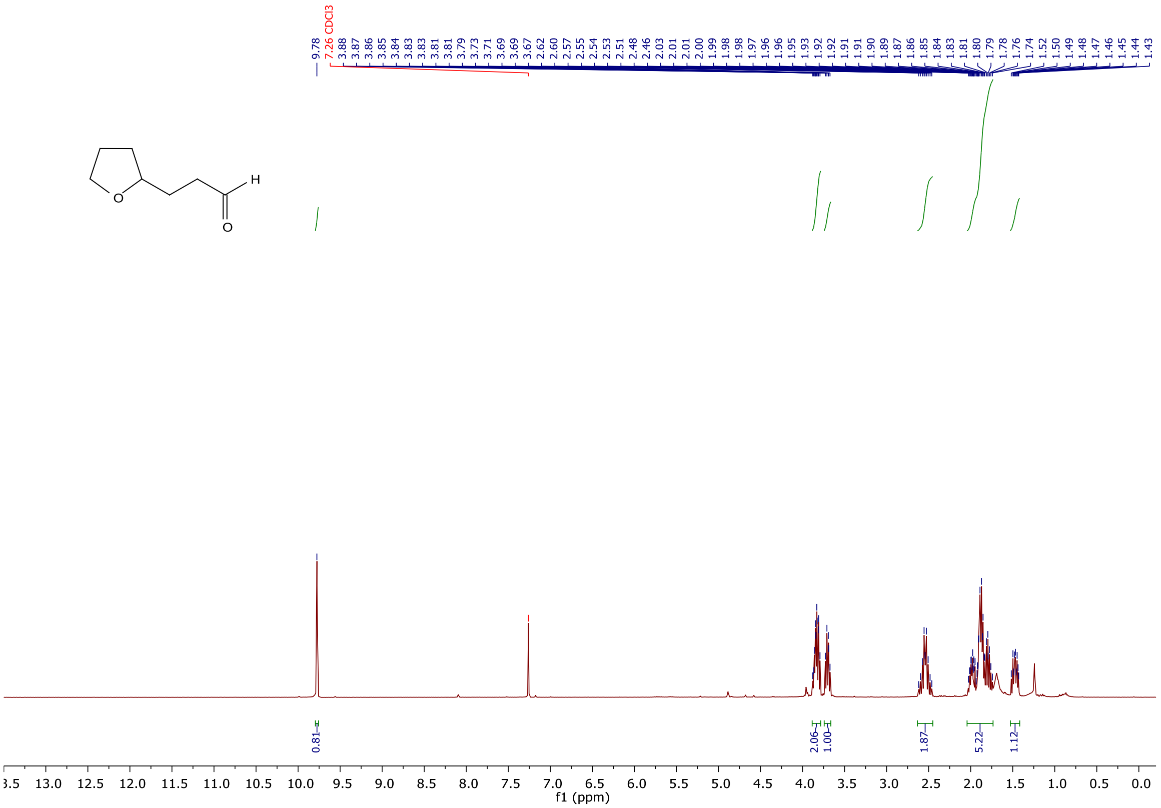

Supplementary Figure 45. ^{1}H NMR (400 MHz, $CDCl_3$) of compound **13**

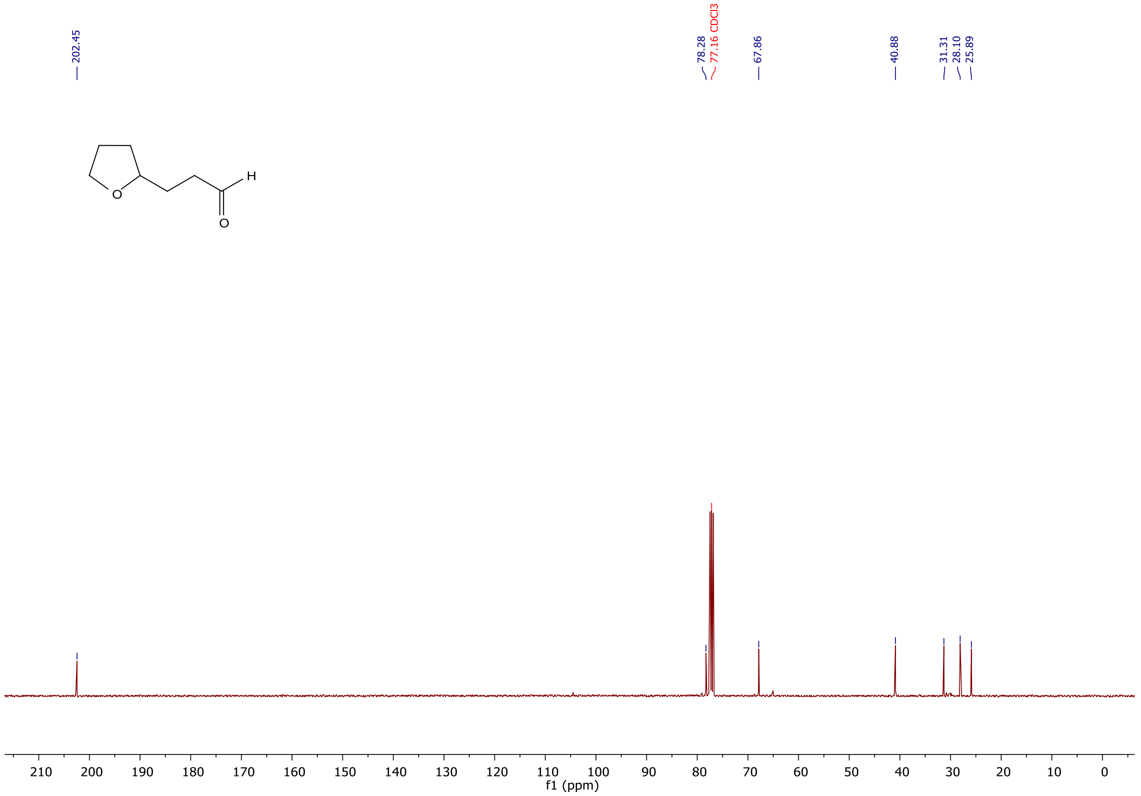

Supplementary Figure 46. ^{13}C NMR (101 MHz, $CDCl_3$) of compound **13**

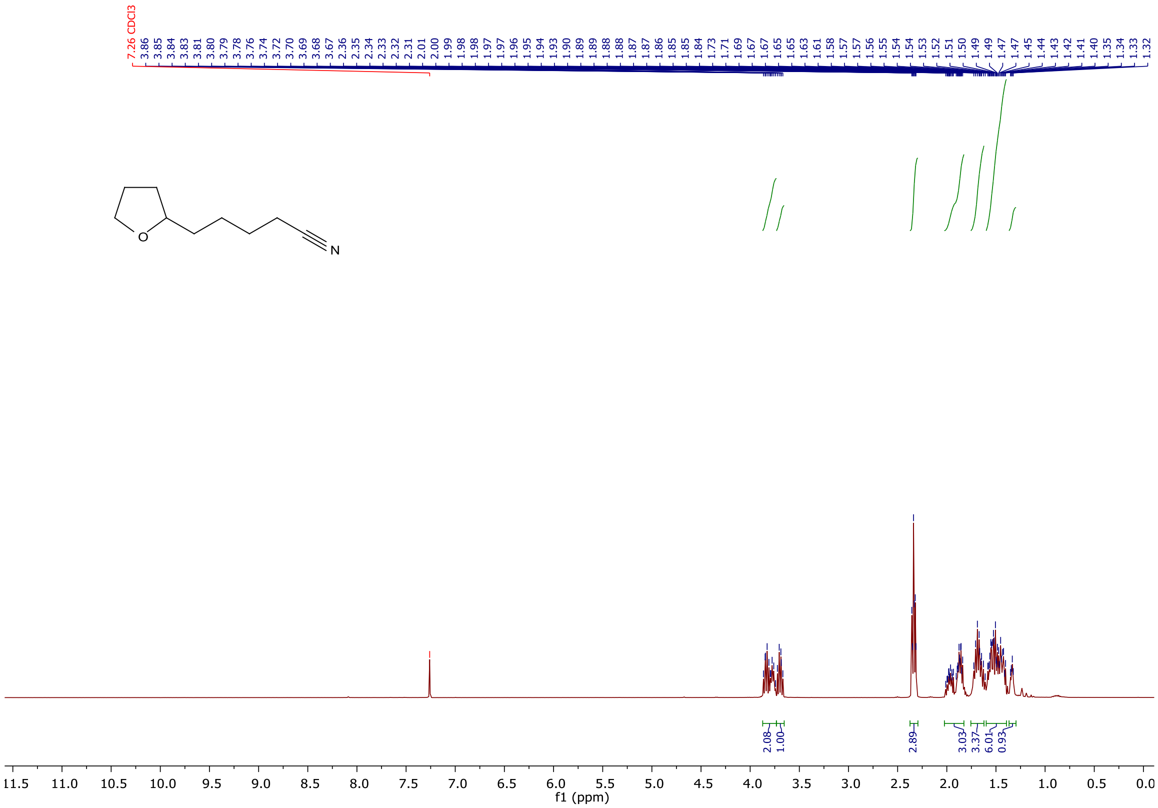

Supplementary Figure 47. ^{1}H NMR (400 MHz, $CDCl_3$) of compound **14**

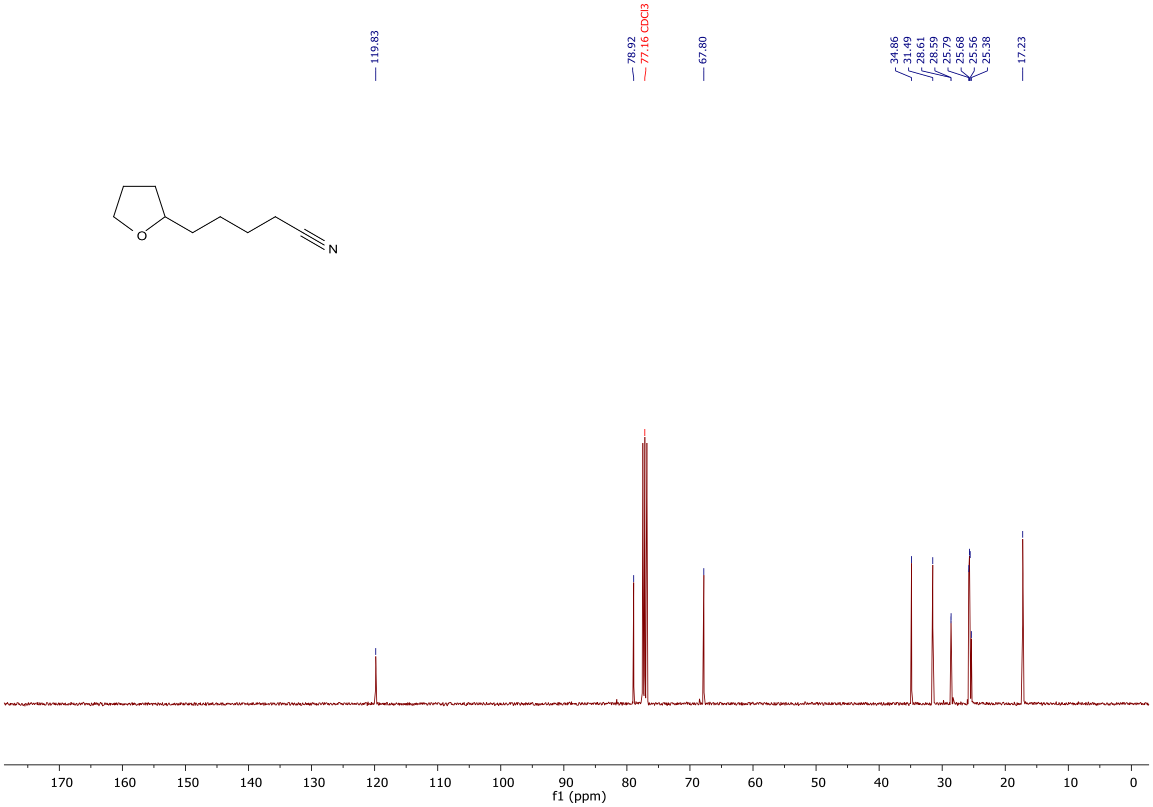

Supplementary Figure 48. ^{13}C NMR (101 MHz, $CDCl_3$) of compound **14**

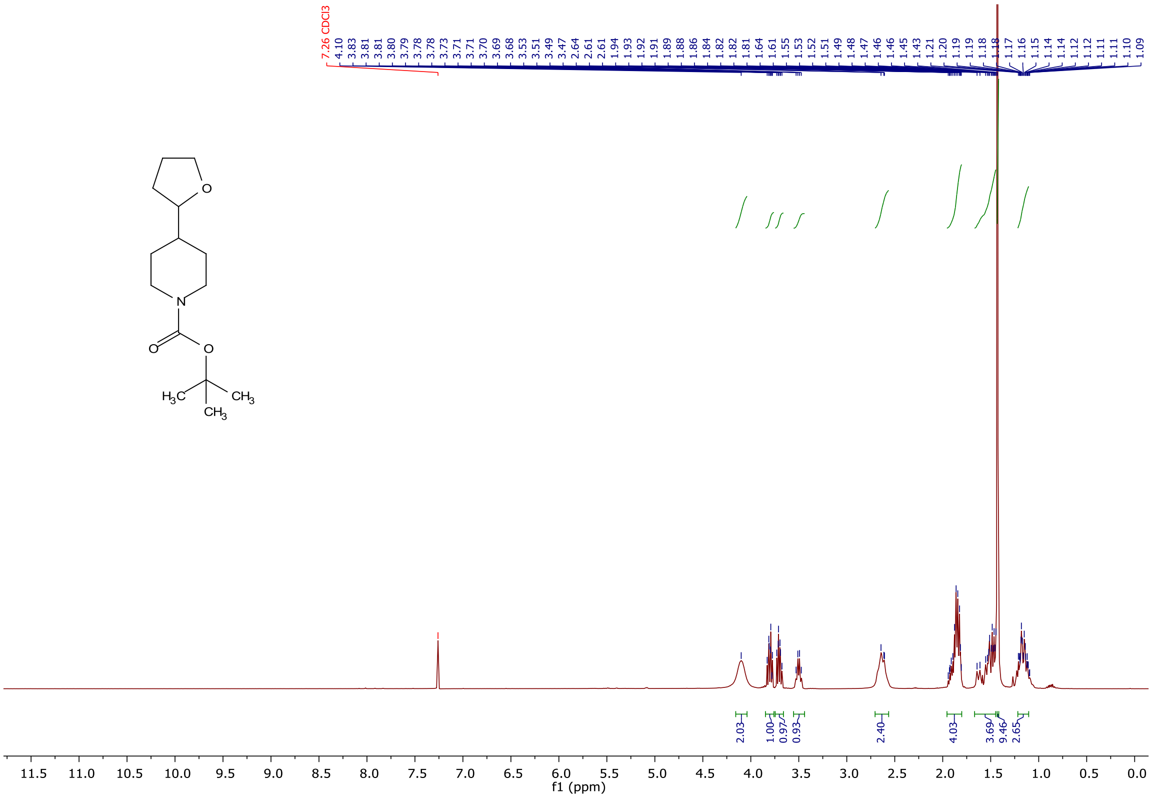

Supplementary Figure 49. ^{1}H NMR (400 MHz, $CDCl_3$) of compound **15**

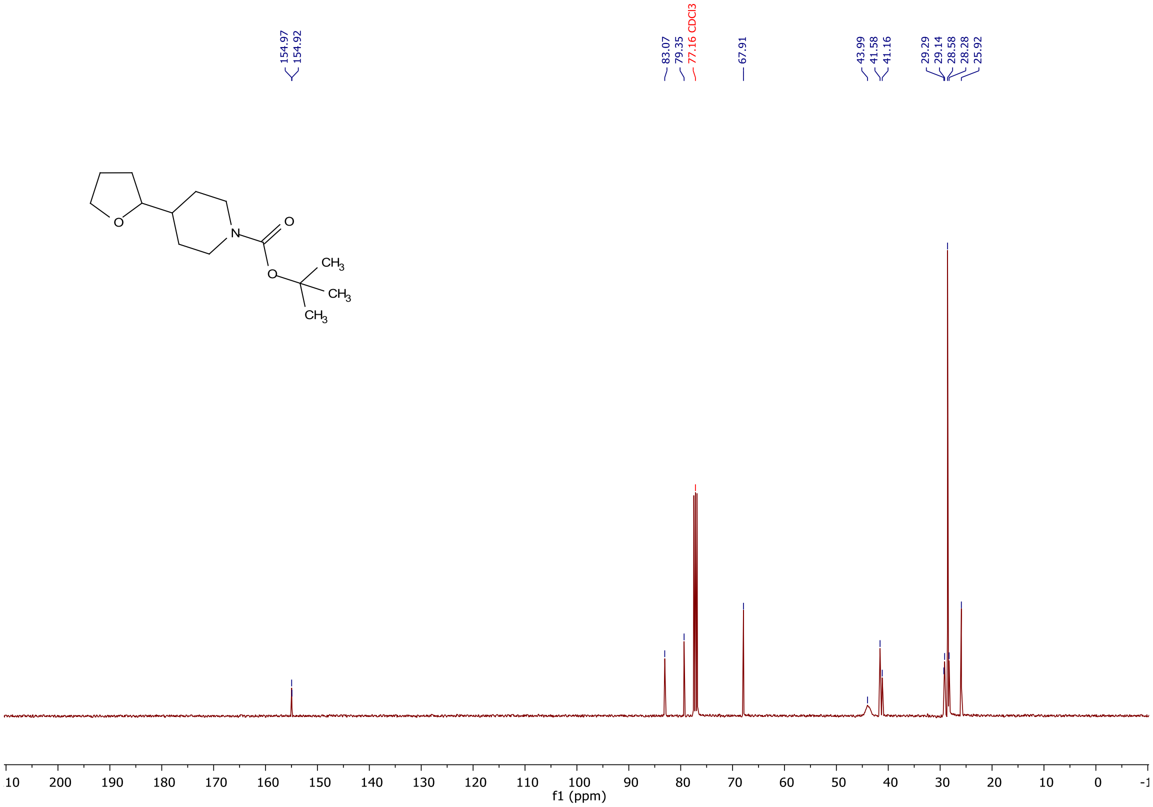

Supplementary Figure 50. ^{13}C NMR (101 MHz, $CDCl_3$) of compound **15**

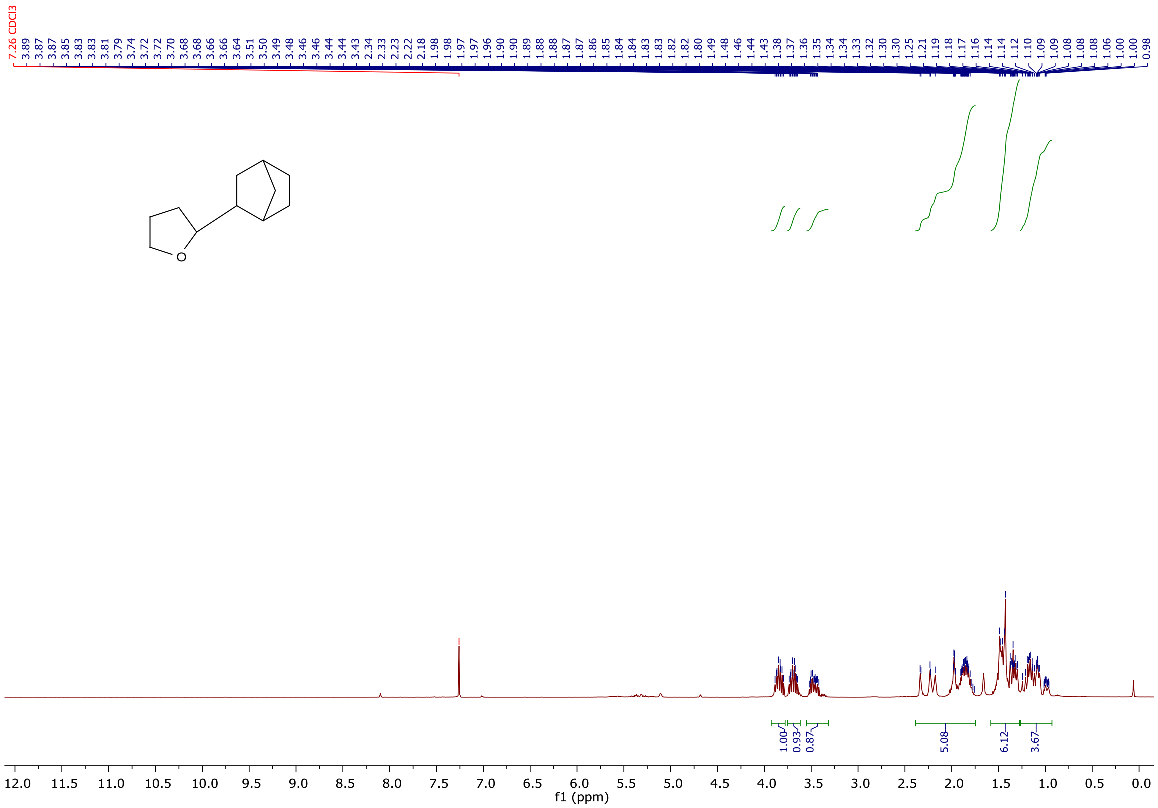

Supplementary Figure 51. ^{1}H NMR (400 MHz, $CDCl_3$) of compound **16**

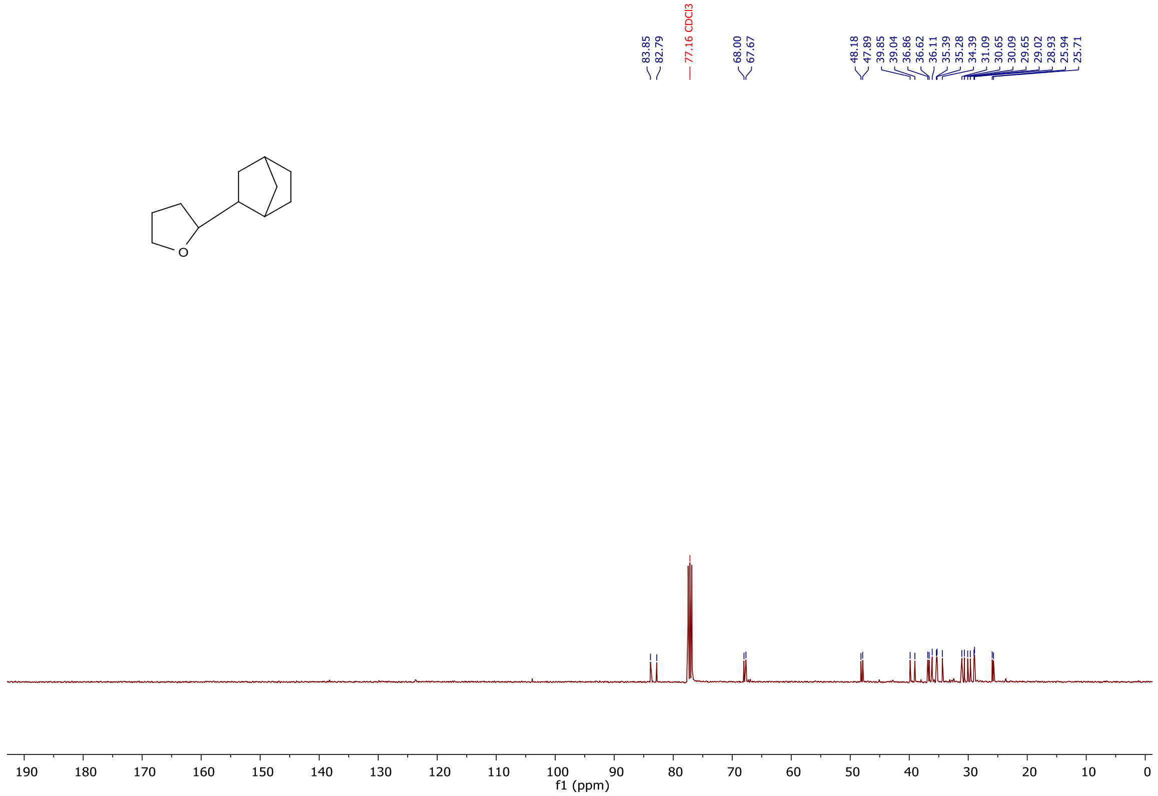

Supplementary Figure 52. ^{13}C NMR (101 MHz, $CDCl_3$) of compound **16**

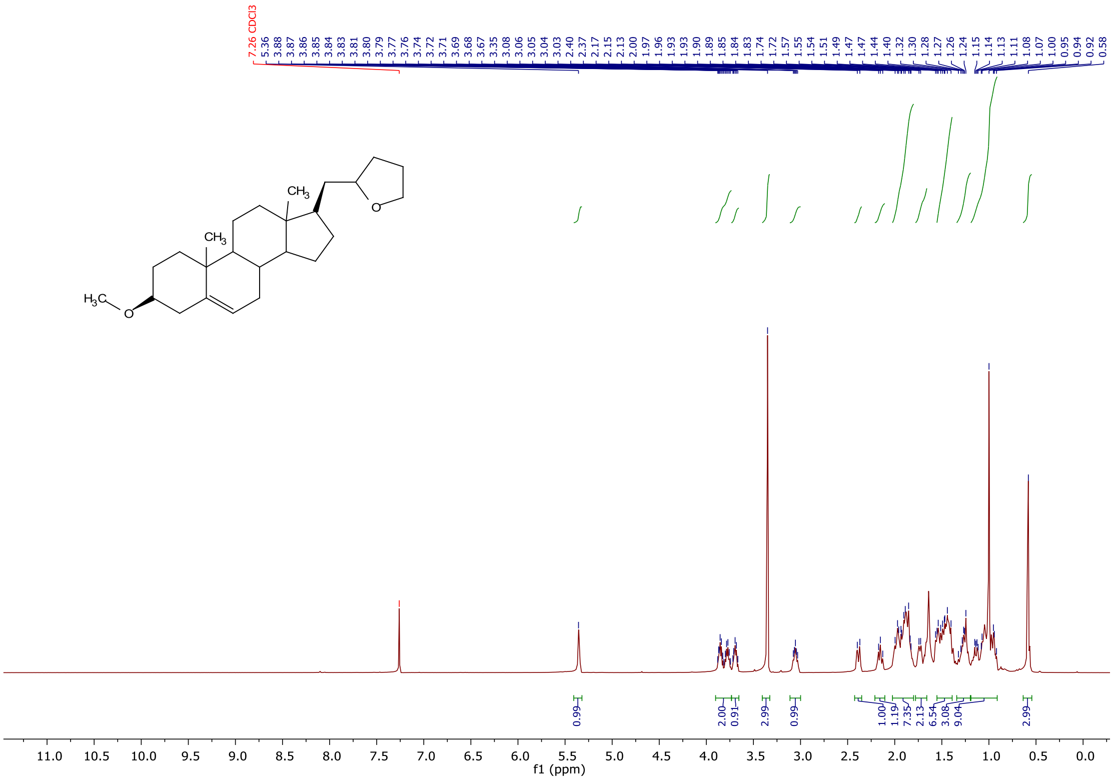

Supplementary Figure 53. ^{1}H NMR (500 MHz, $CDCl_3$) of compound **17**

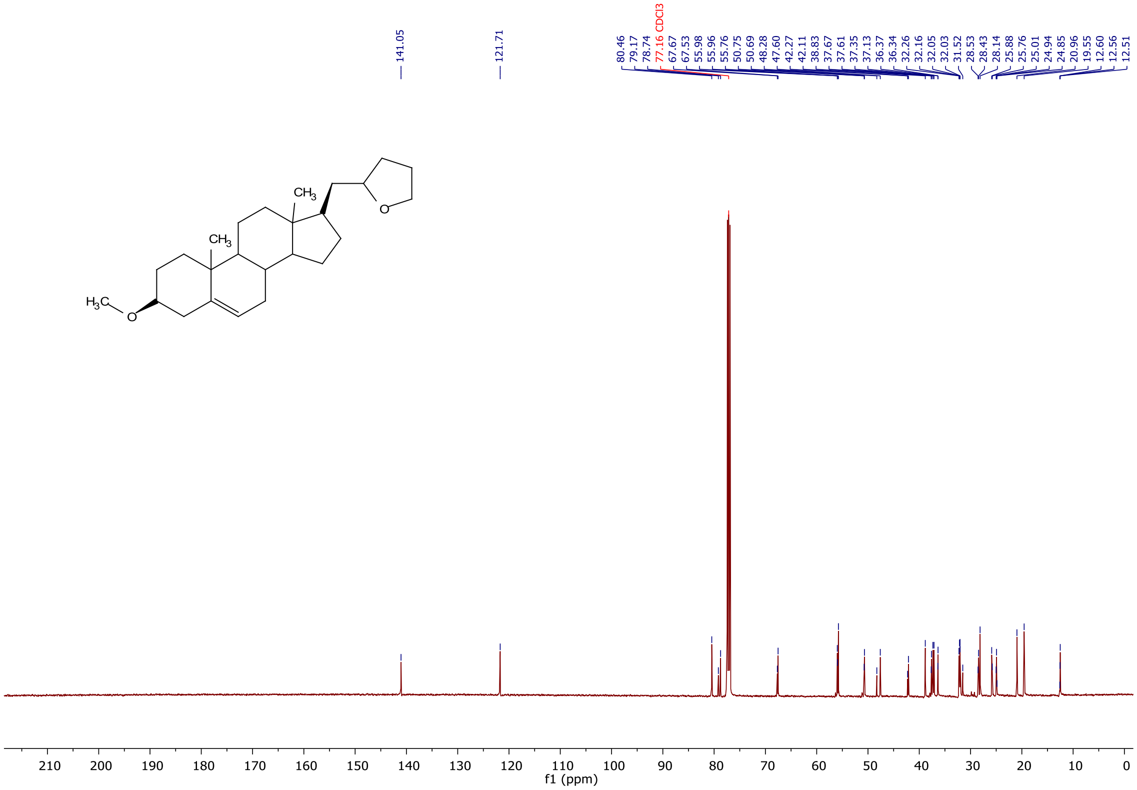

Supplementary Figure 54. ^{13}C NMR (126 MHz, $CDCl_3$) of compound **17**

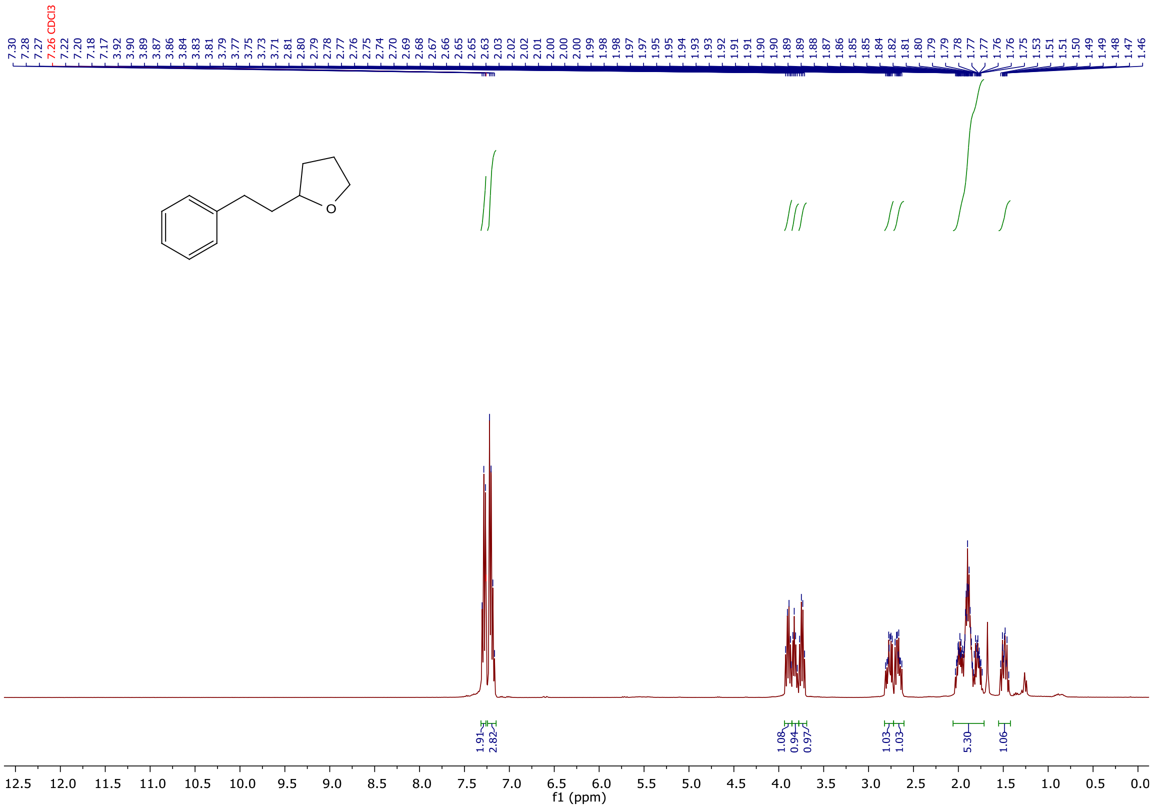

Supplementary Figure 55. ^{1}H NMR (400 MHz, $CDCl_3$) of compound **18**

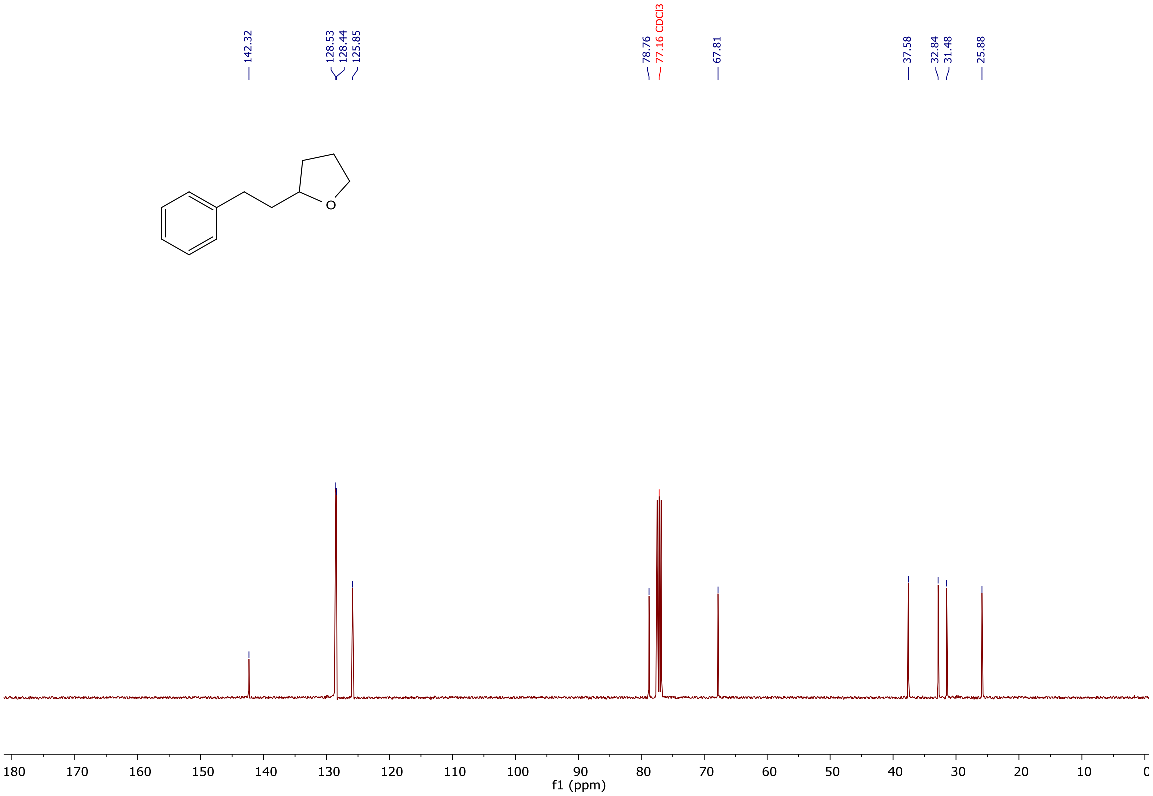

Supplementary Figure 56. ^{13}C NMR (101 MHz, $CDCl_3$) of compound **18**

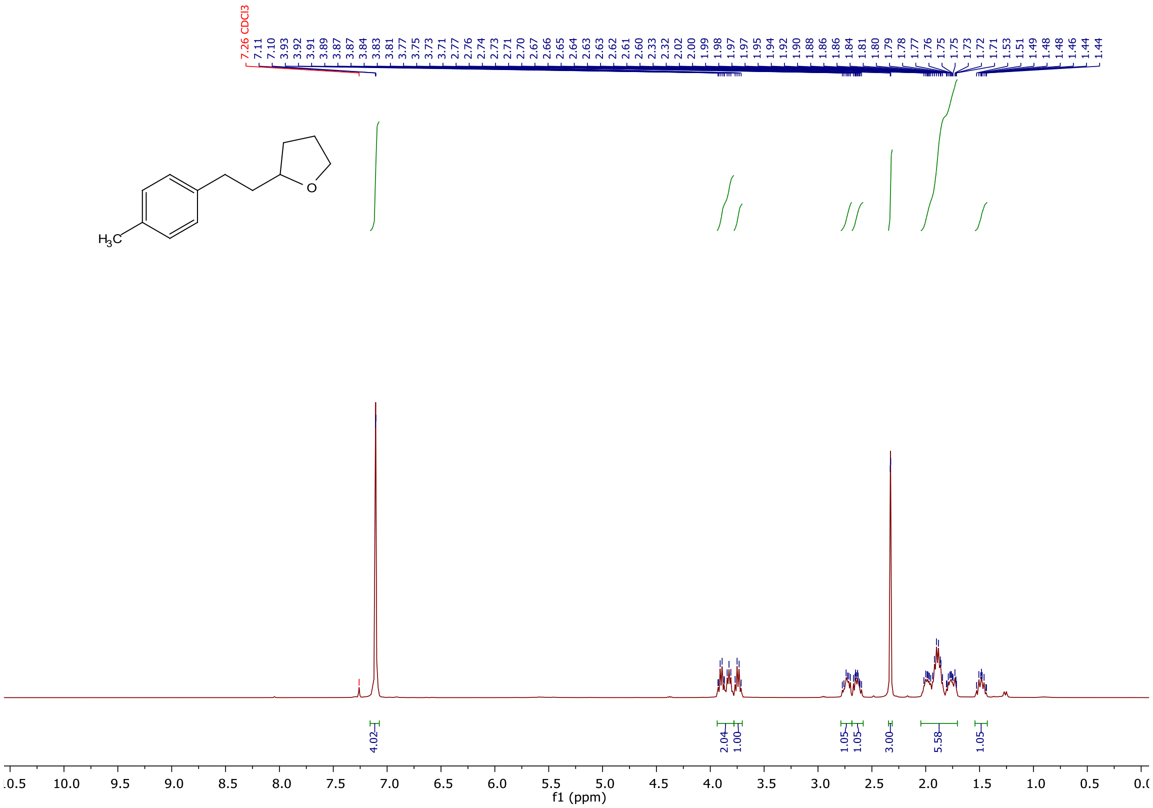

Supplementary Figure 57. ^{1}H NMR (400 MHz, $CDCl_3$) of compound **19**

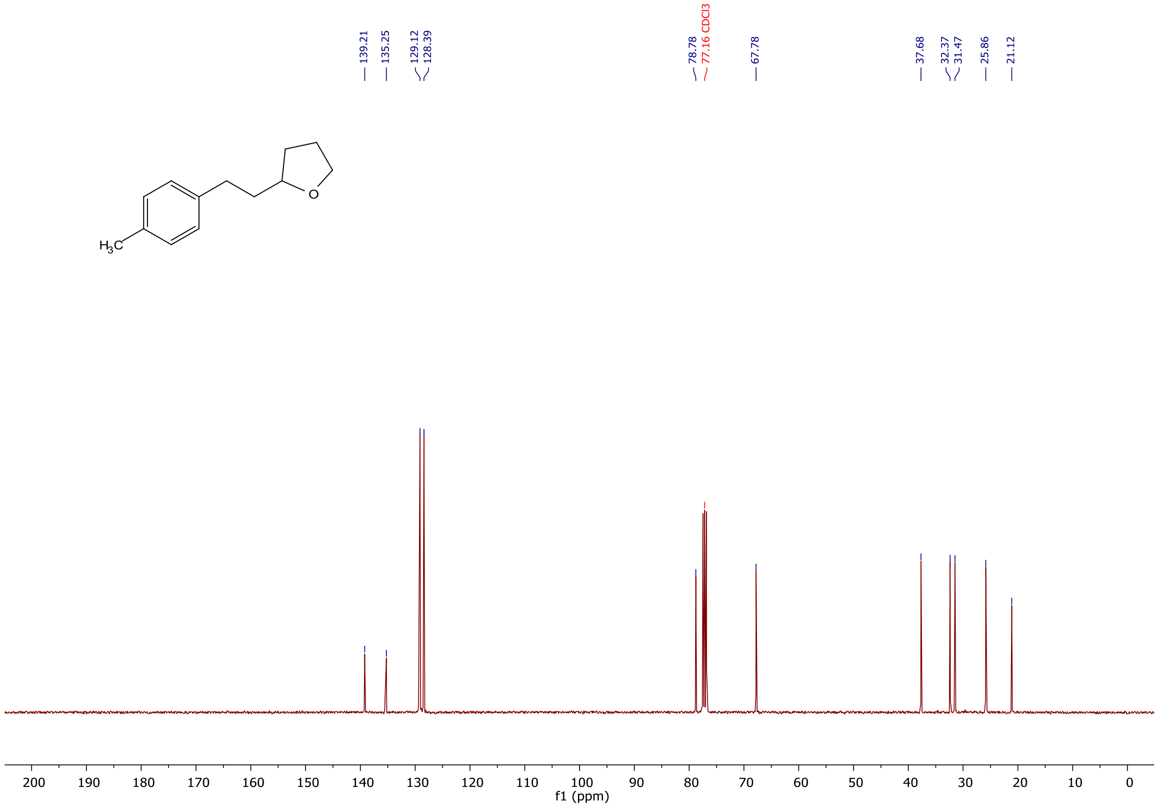

Supplementary Figure 58. ^{13}C NMR (101 MHz, $CDCl_3$) of compound **19**

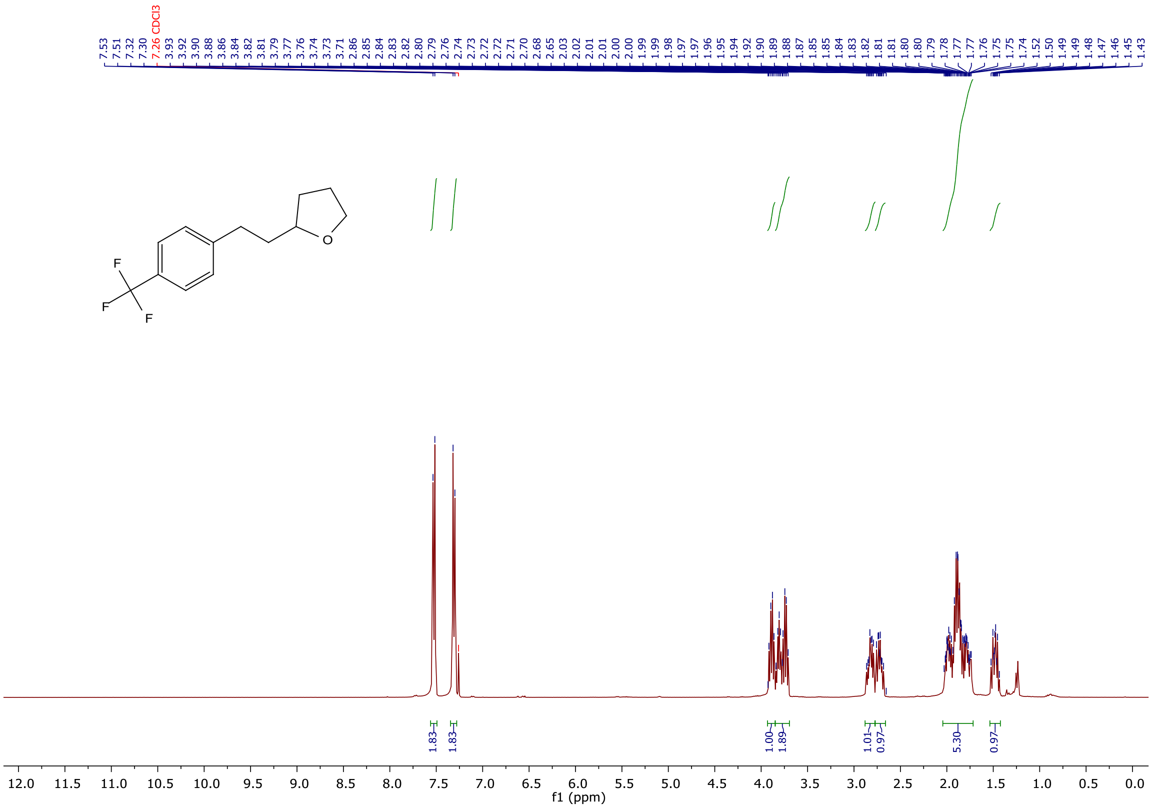

Supplementary Figure 59. ^{1}H NMR (400 MHz, $CDCl_3$) of compound **20**

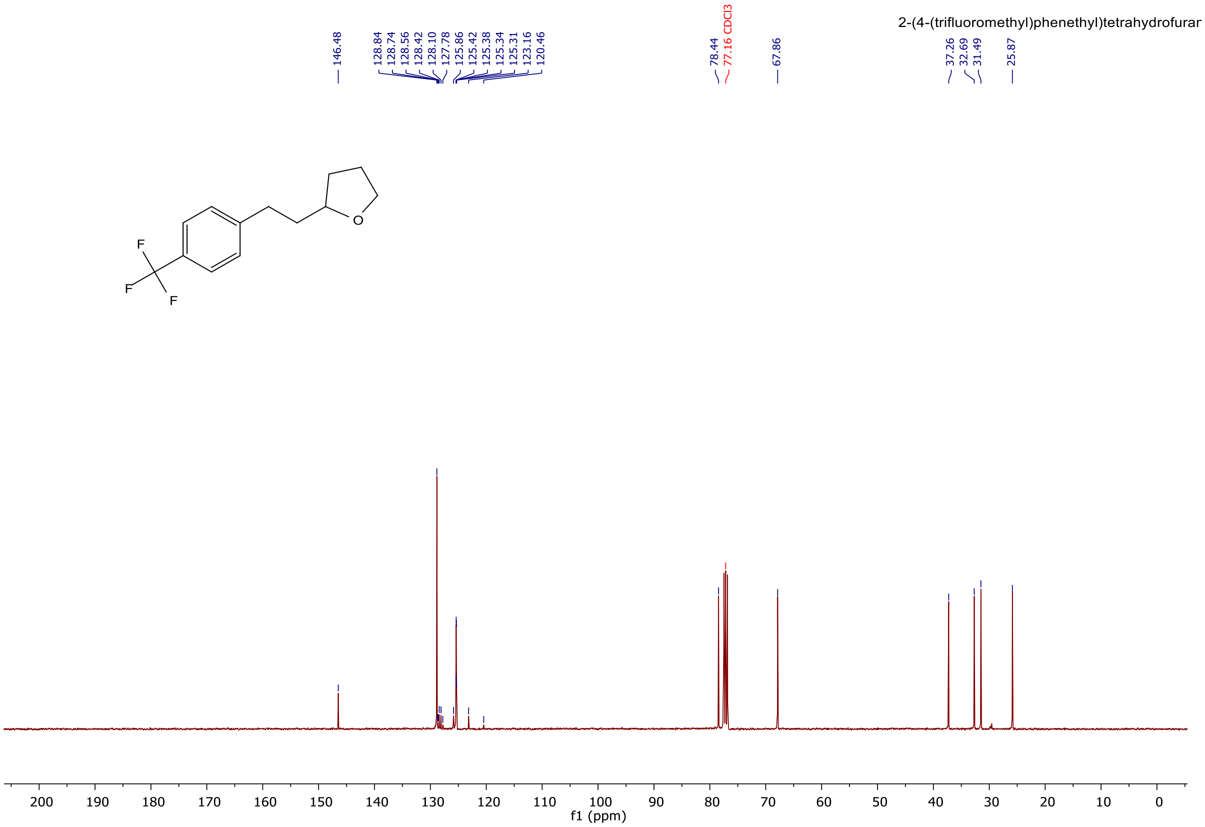

Supplementary Figure 60. ^{13}C NMR (101 MHz, $CDCl_3$) of compound **20**

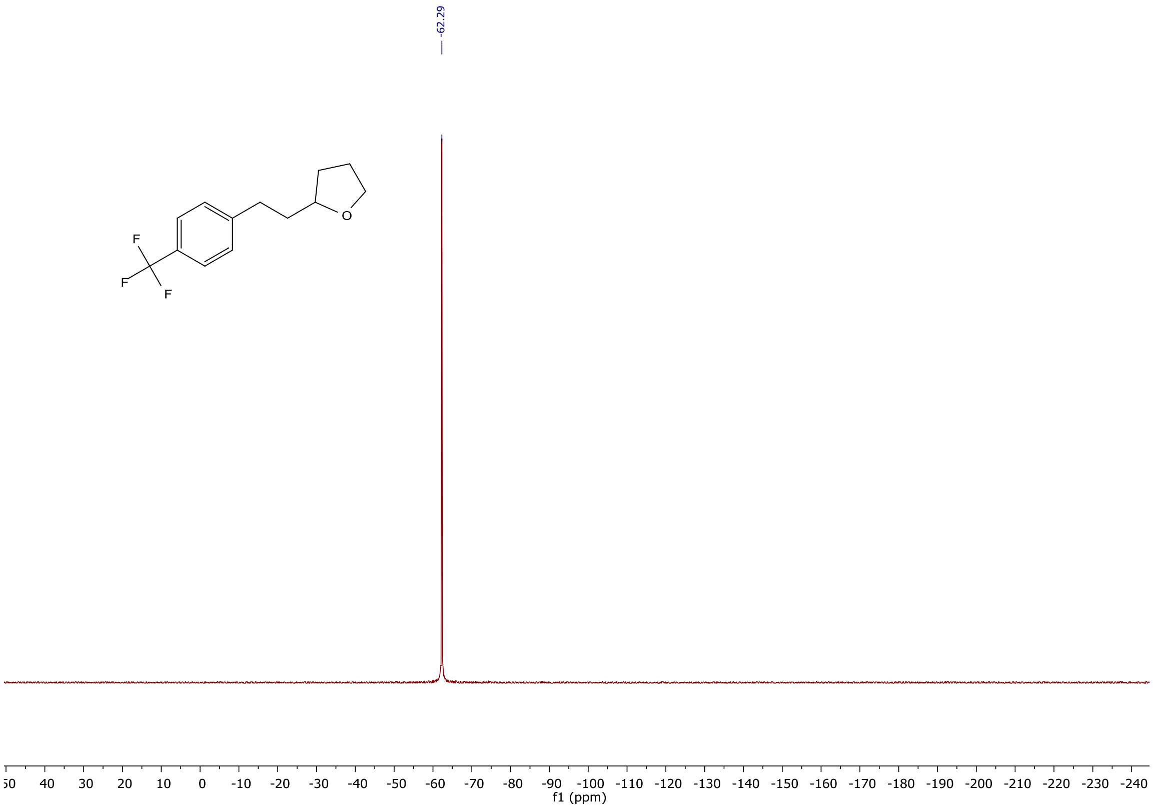

Supplementary Figure 61. ^{19}F NMR (377 MHz, $CDCl_3$) of compound **20**

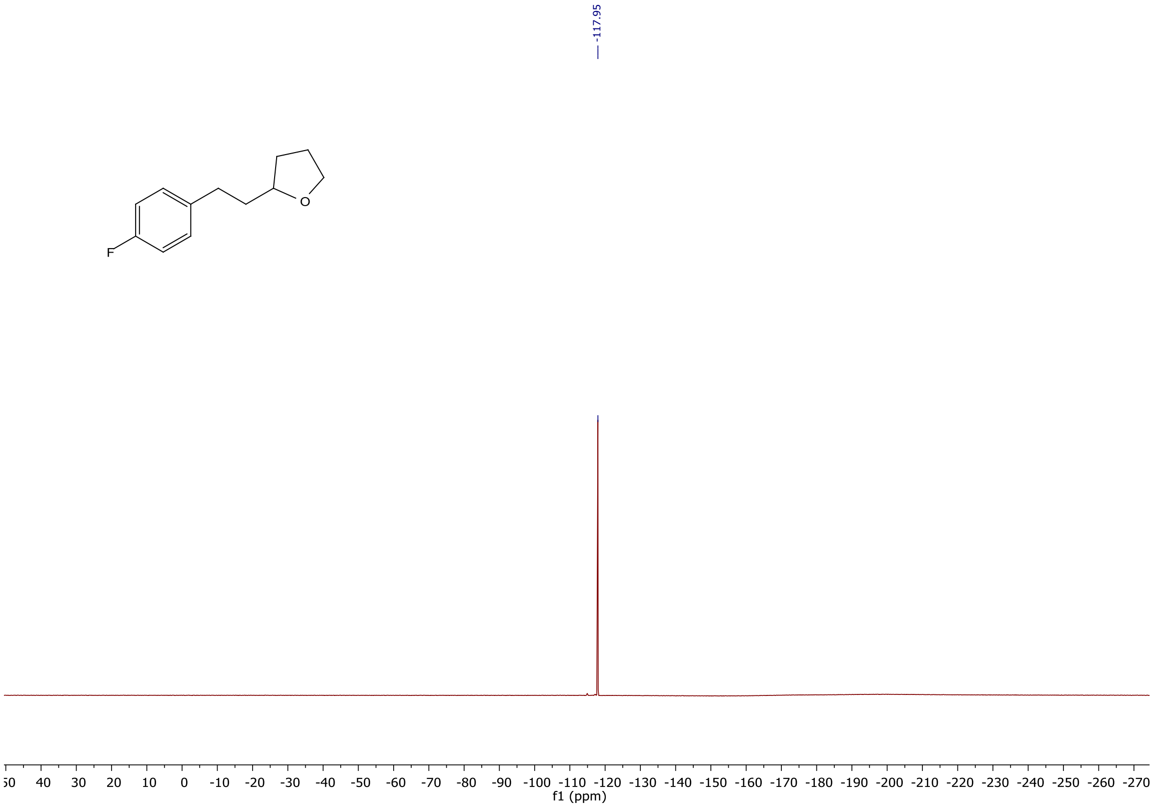

Supplementary Figure 62. ^{19}F NMR (377 MHz, $CDCl_3$) of compound **21**

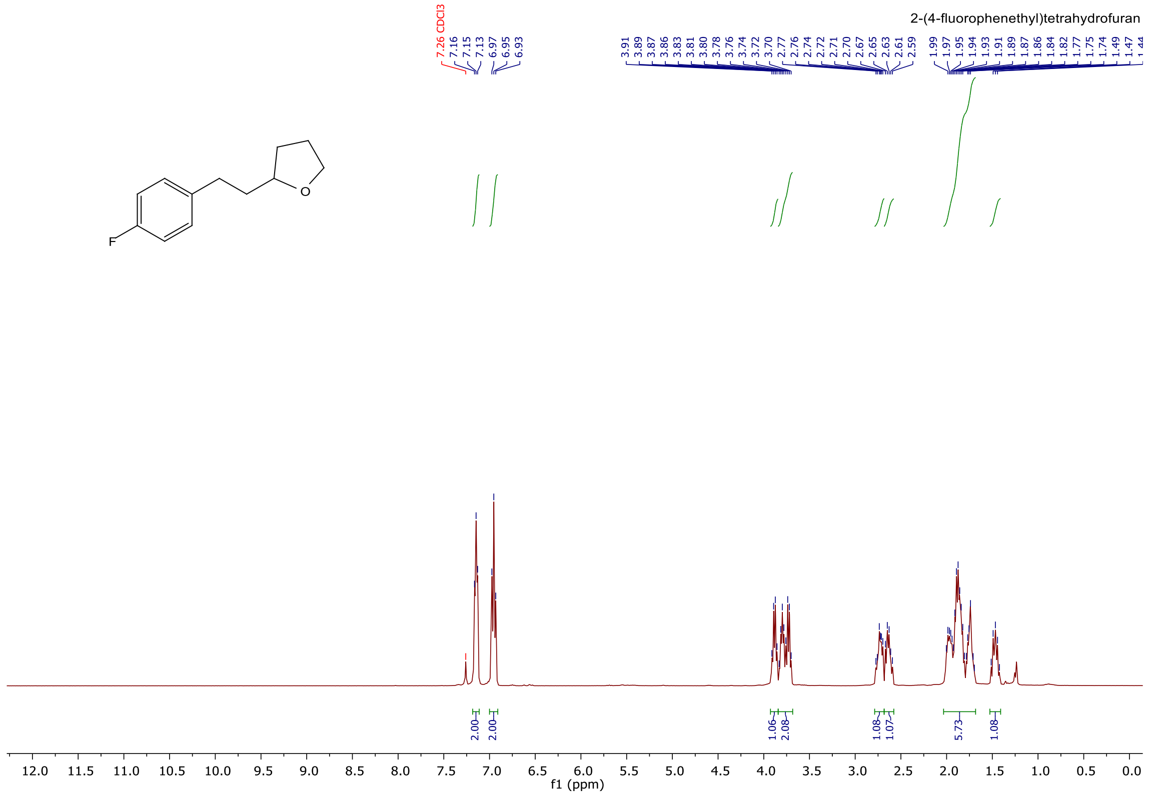

Supplementary Figure 63. ^{1}H NMR (400 MHz, $CDCl_3$) of compound **21**

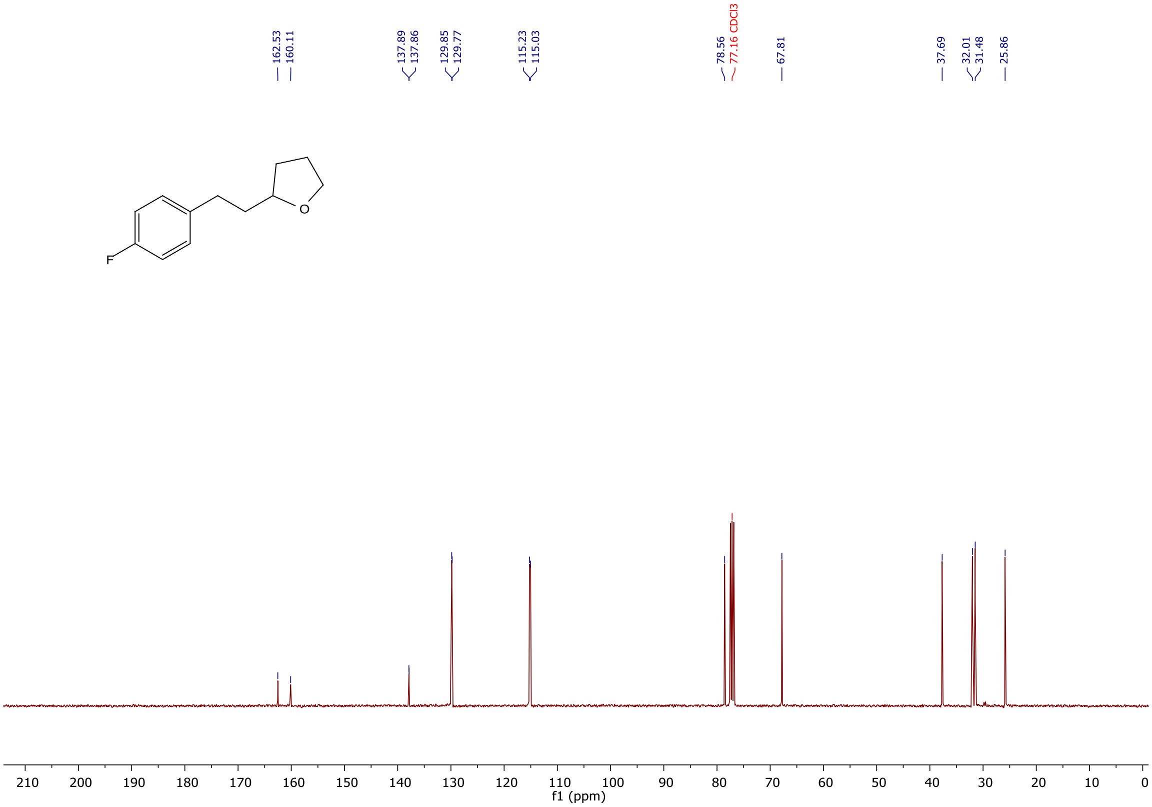

Supplementary Figure 64. ^{13}C NMR (101 MHz, $CDCl_3$) of compound **21**

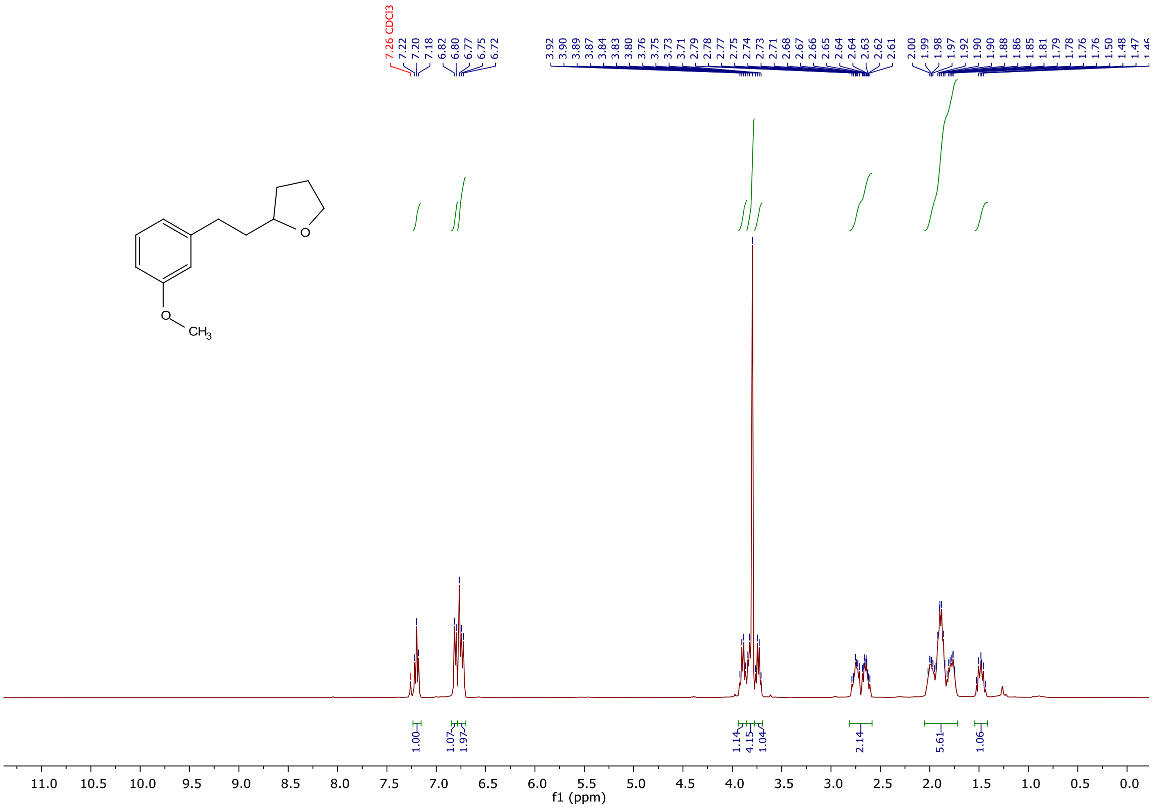

Supplementary Figure 65. ^{1}H NMR (400 MHz, $CDCl_3$) of compound **22**

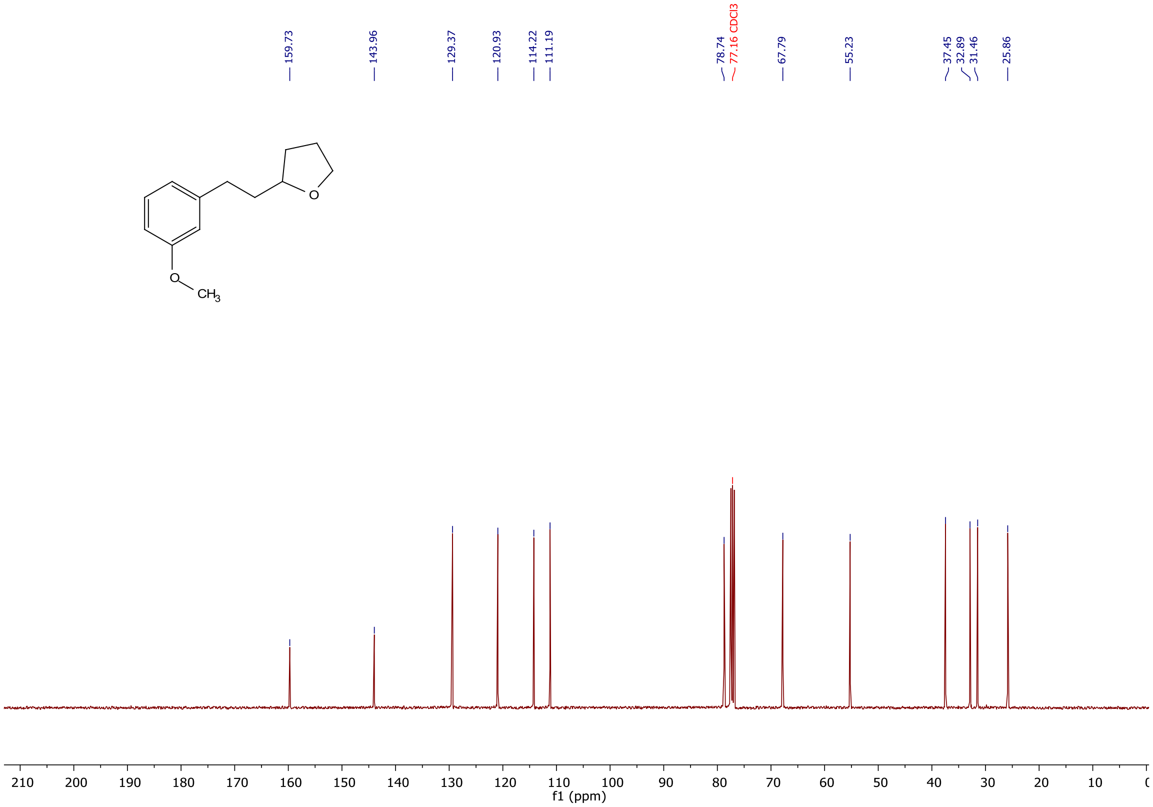

Supplementary Figure 66. ^{13}C NMR (101 MHz, $CDCl_3$) of compound **22**

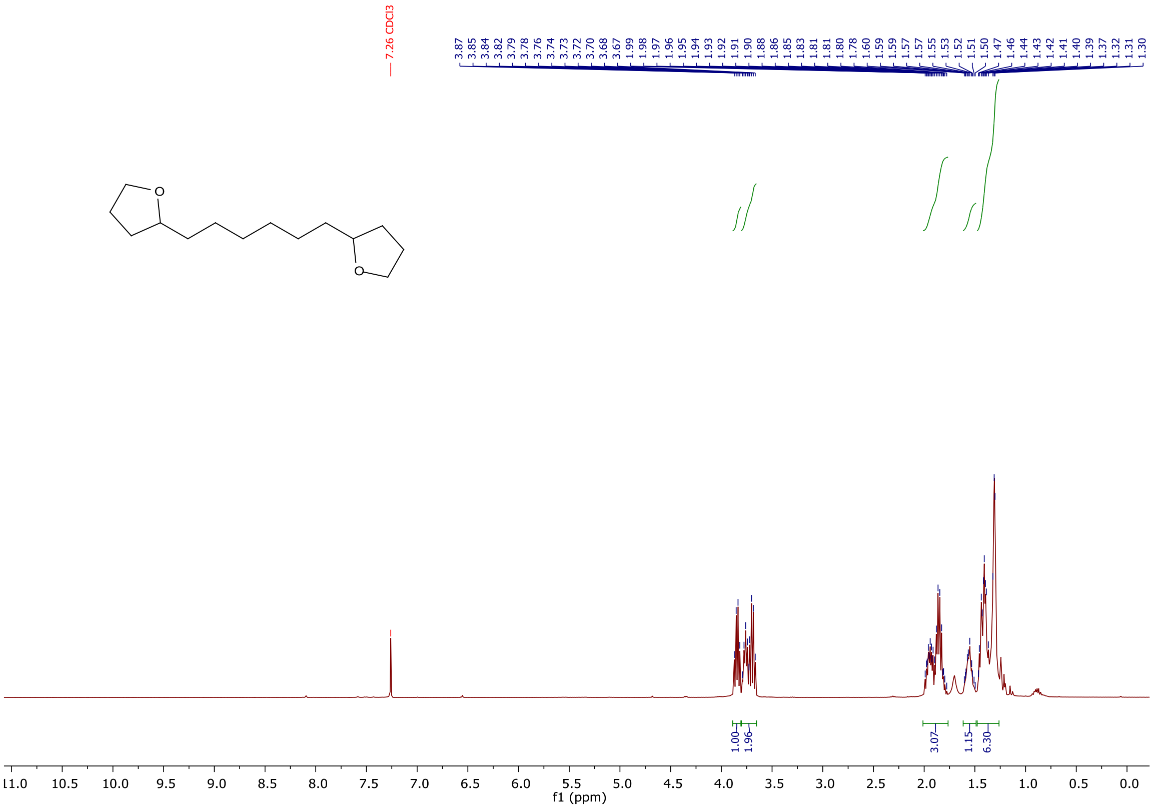

Supplementary Figure 67. ^{1}H NMR (400 MHz, $CDCl_3$) of compound **23**

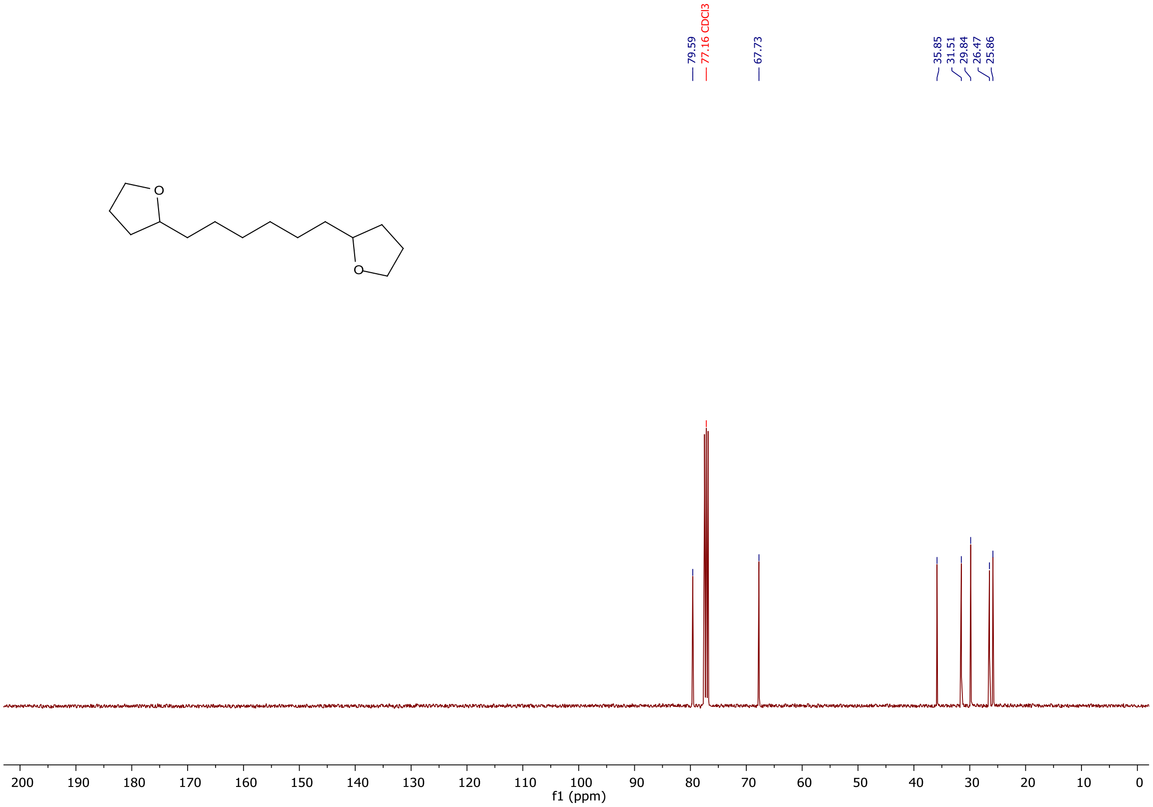

Supplementary Figure 68. ^{13}C NMR (101 MHz, $CDCl_3$) of compound **23**

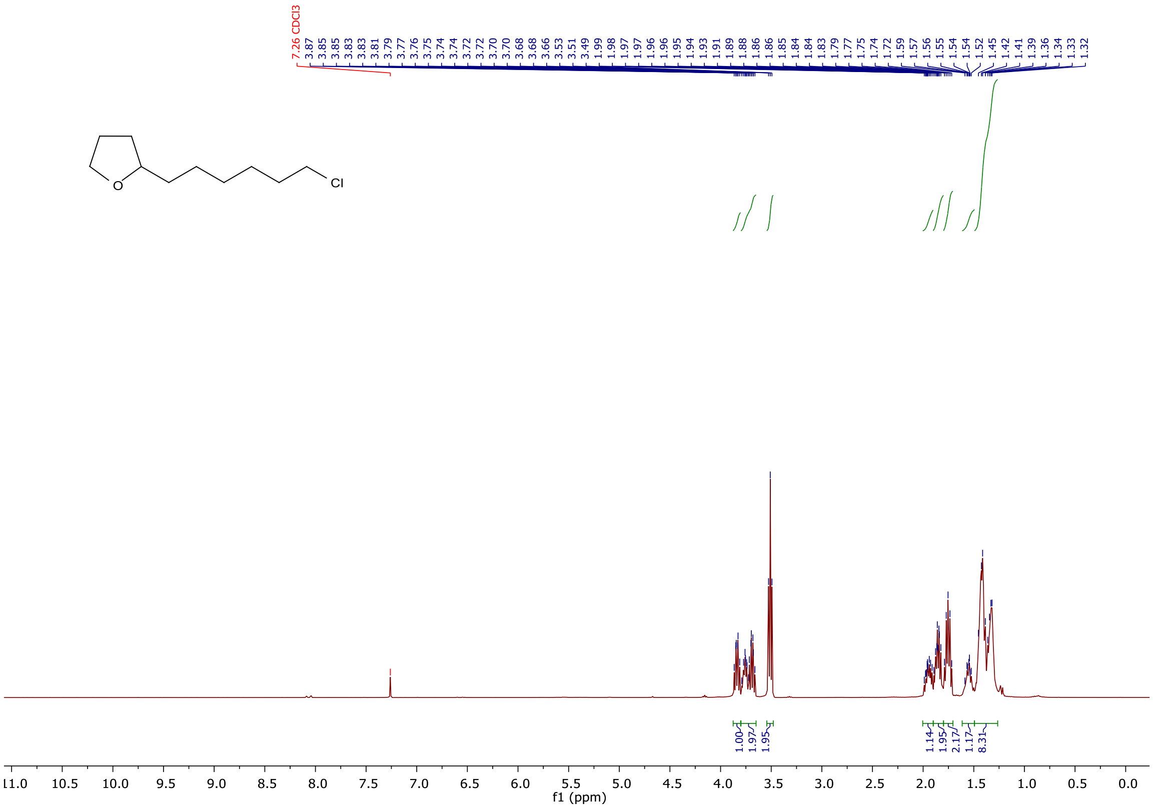

Supplementary Figure 69. ^{1}H NMR (400 MHz, $CDCl_3$) of compound **24**

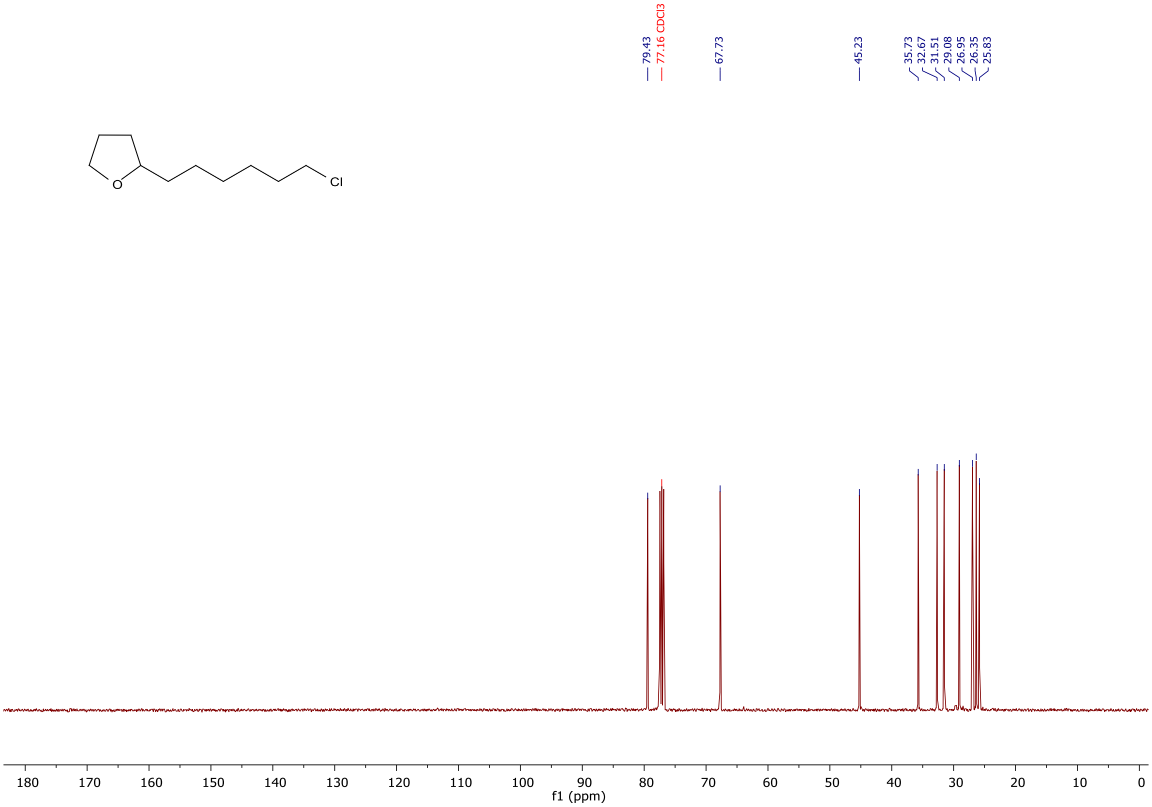

Supplementary Figure 70. ^{13}C NMR (101 MHz, $CDCl_3$) of compound **24**

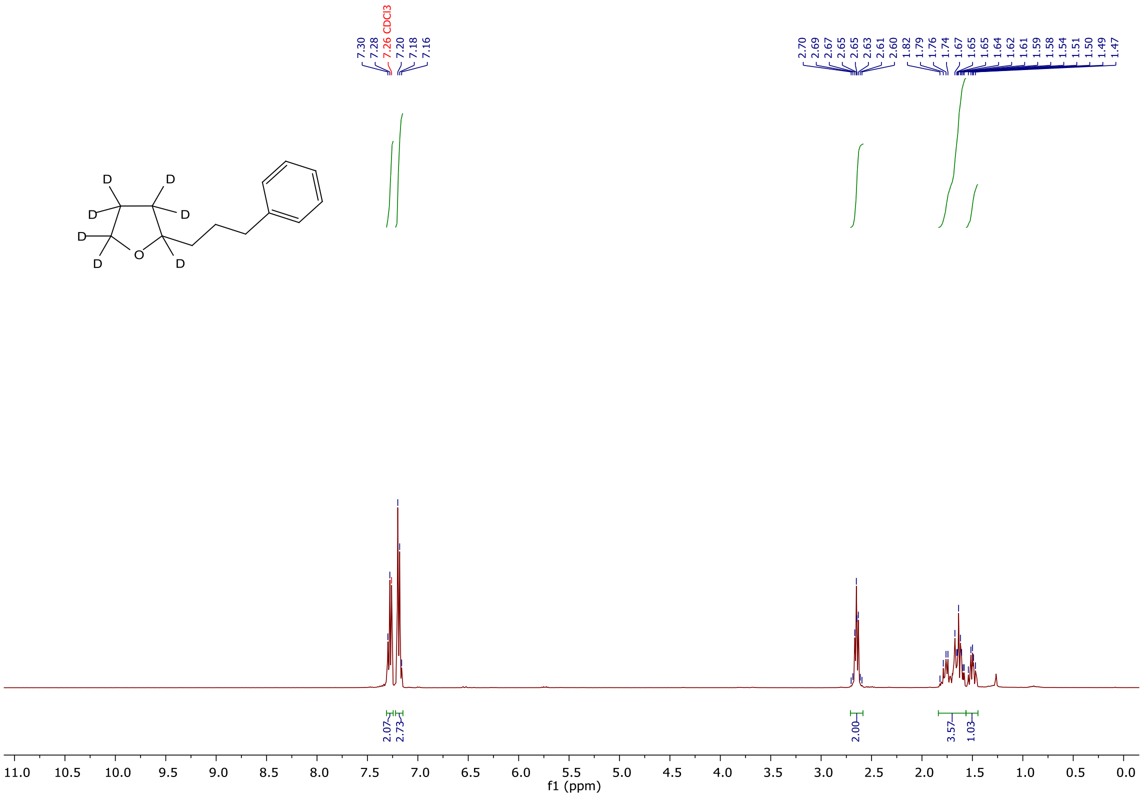

Supplementary Figure 71. ^{1}H NMR (400 MHz, $CDCl_3$) of compound **25**

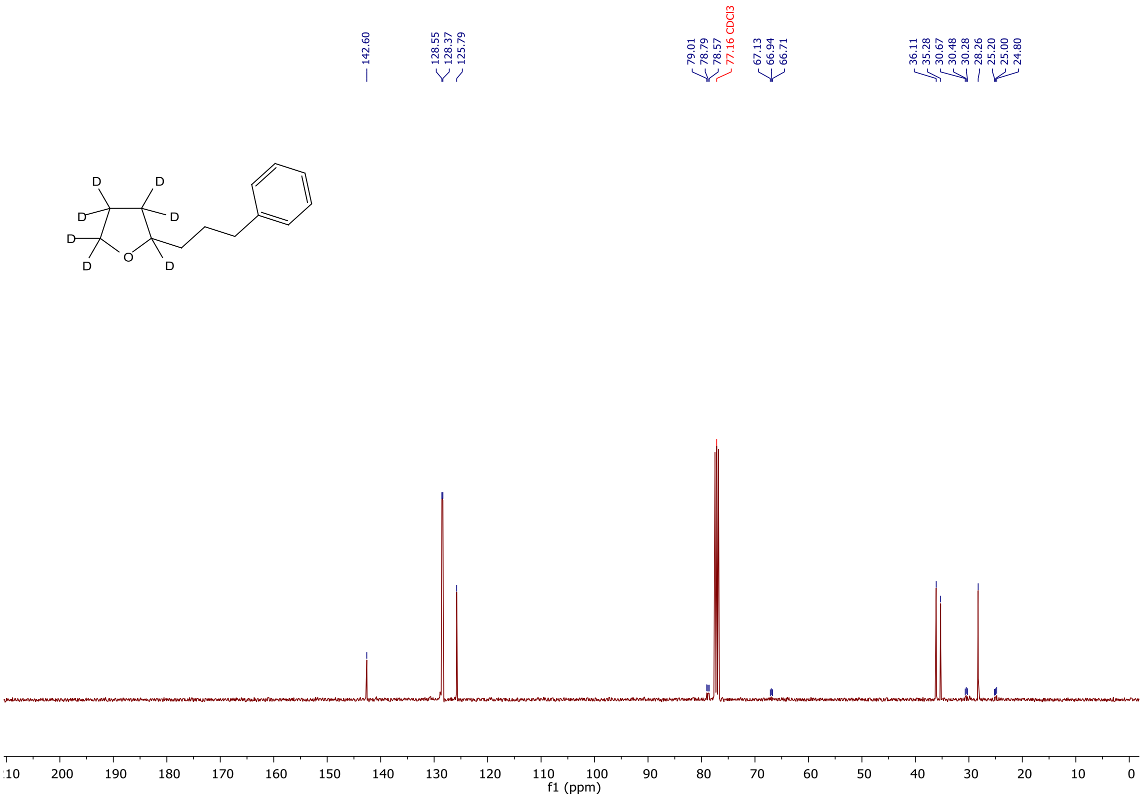

Supplementary Figure 72. ^{13}C NMR (101 MHz, $CDCl_3$) of compound **25**

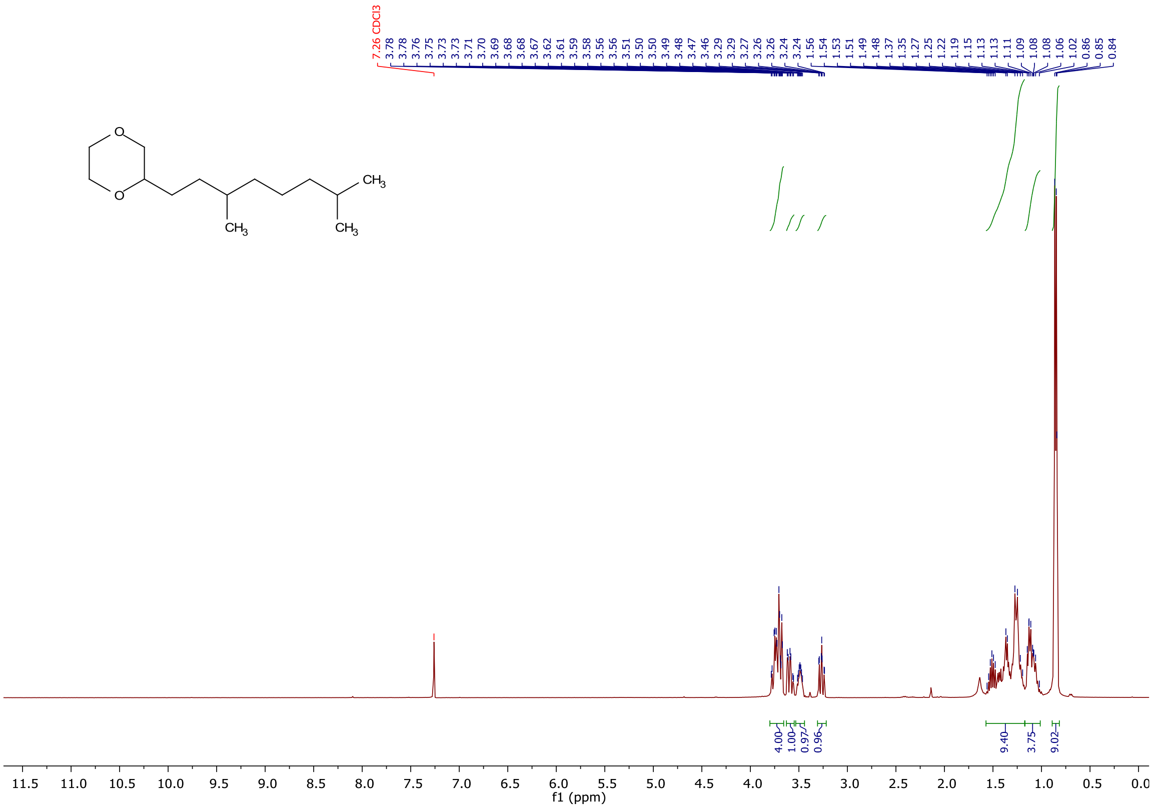

Supplementary Figure 73. ^{1}H NMR (400 MHz, $CDCl_3$) of compound **26**

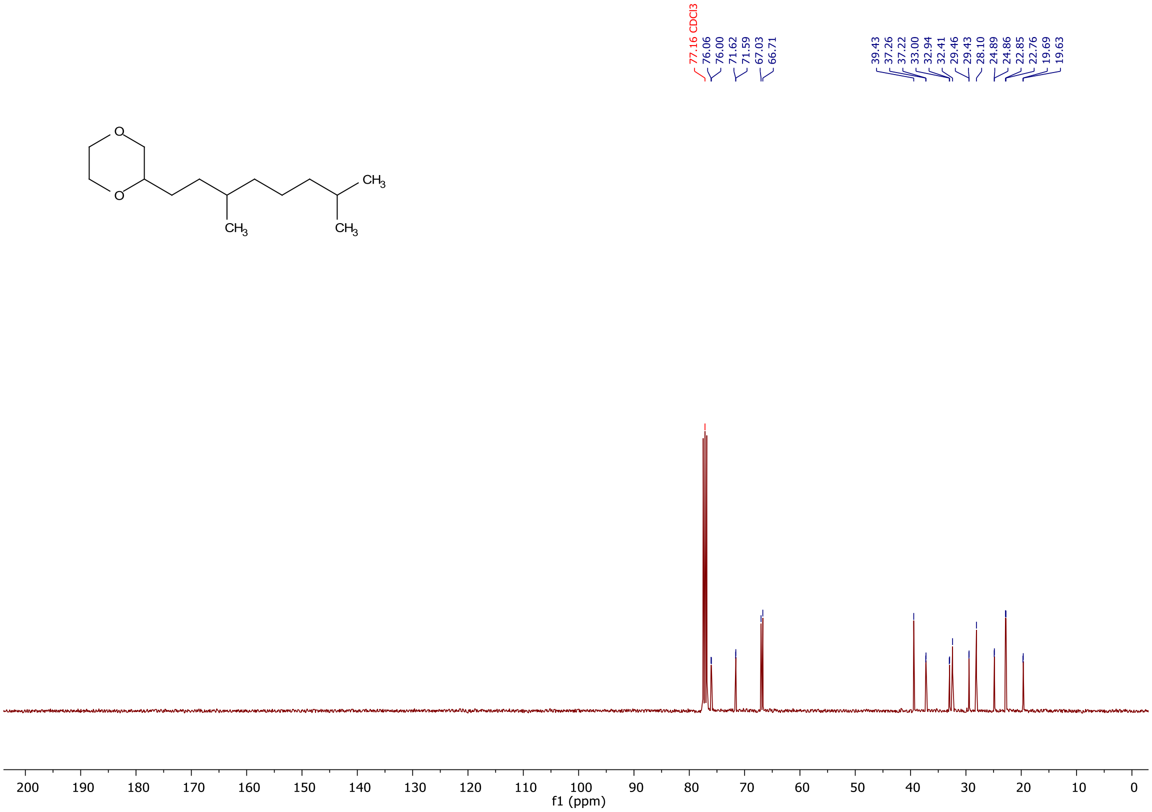

Supplementary Figure 74. ^{13}C NMR (101 MHz, $CDCl_3$) of compound **26**

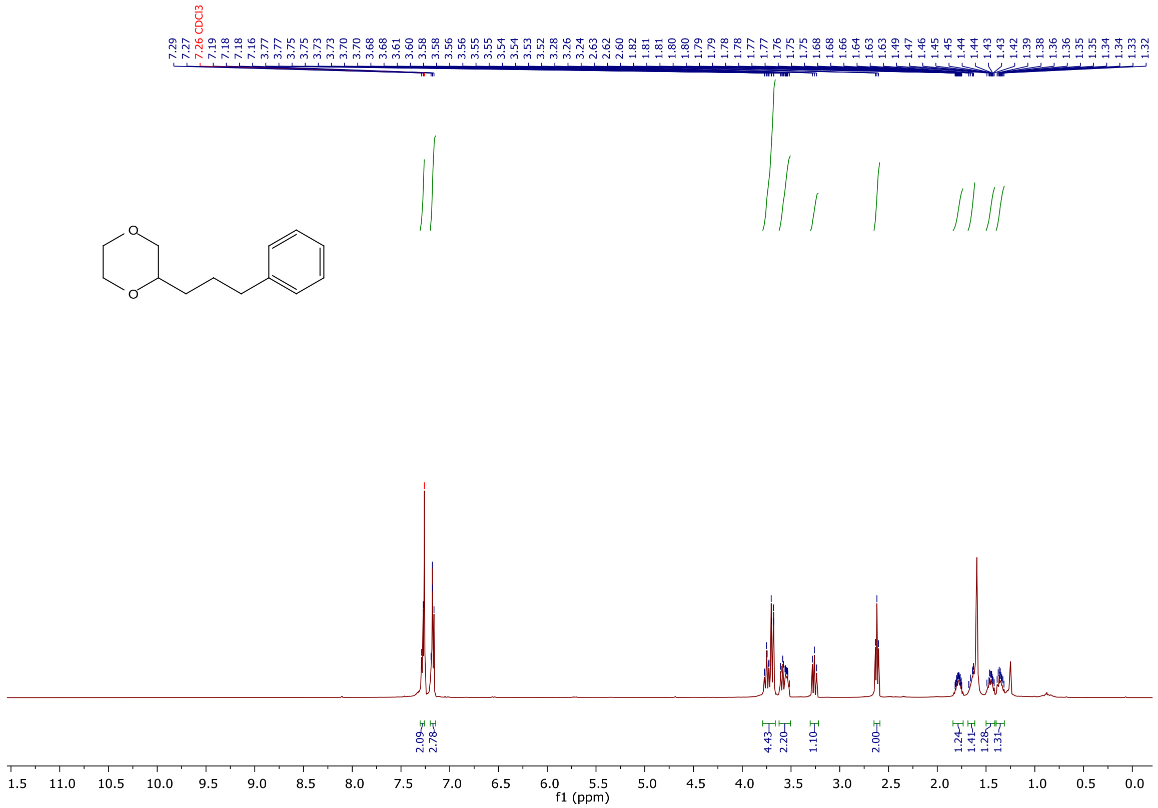

Supplementary Figure 75. ^{1}H NMR (500 MHz, $CDCl_3$) of compound **27**

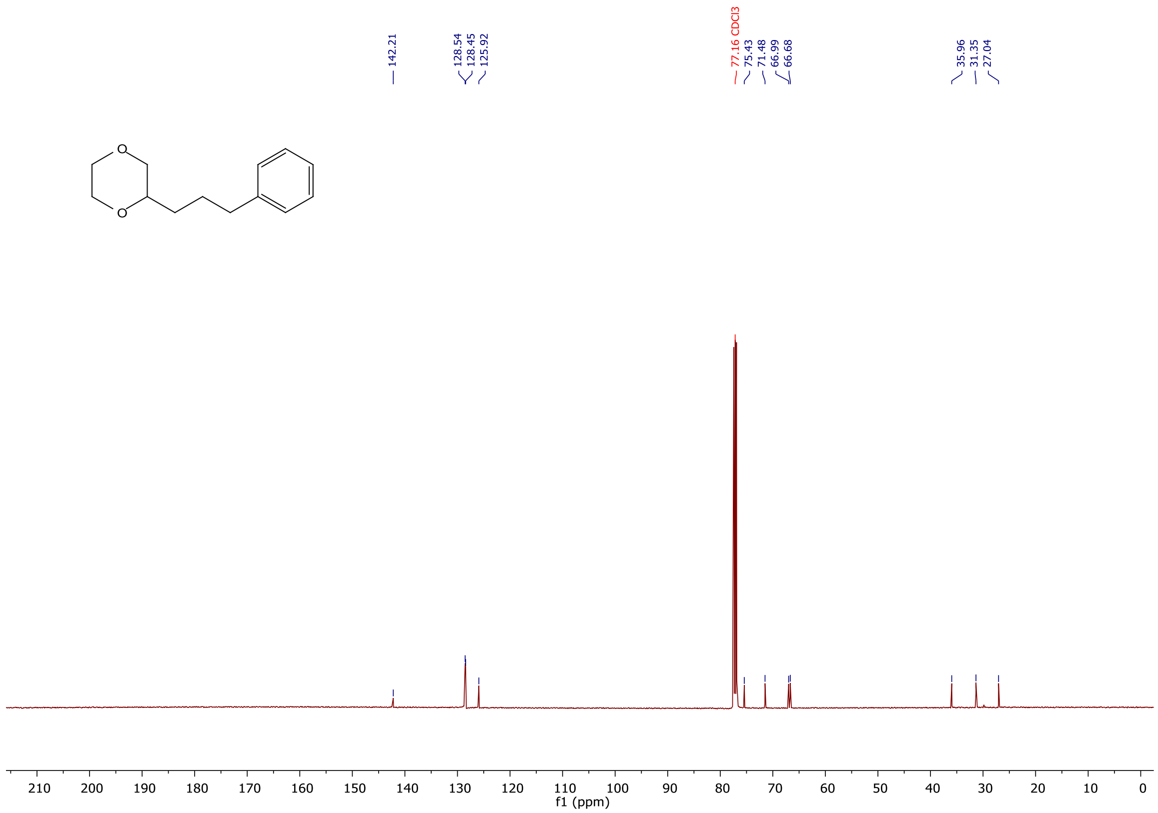

Supplementary Figure 76. ^{13}C NMR (126 MHz, $CDCl_3$) of compound **27**

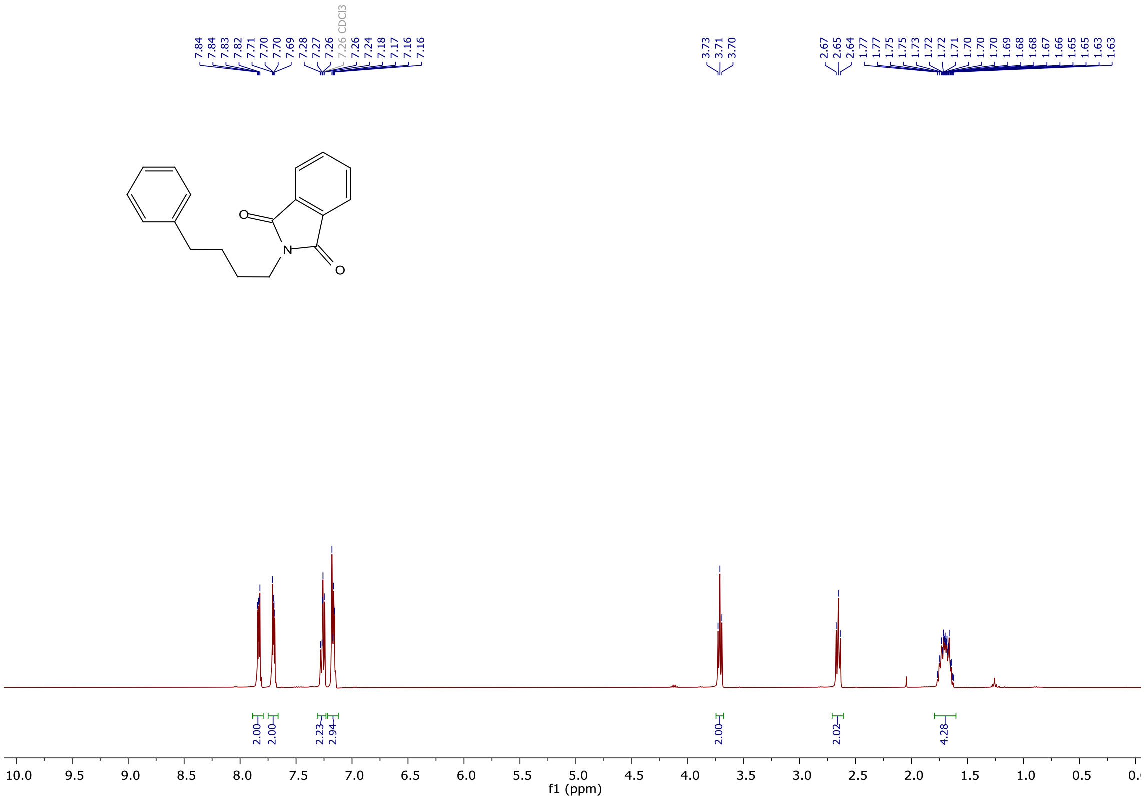

Supplementary Figure 77. ^{1}H NMR (400 MHz, $CDCl_3$) of compound **28**

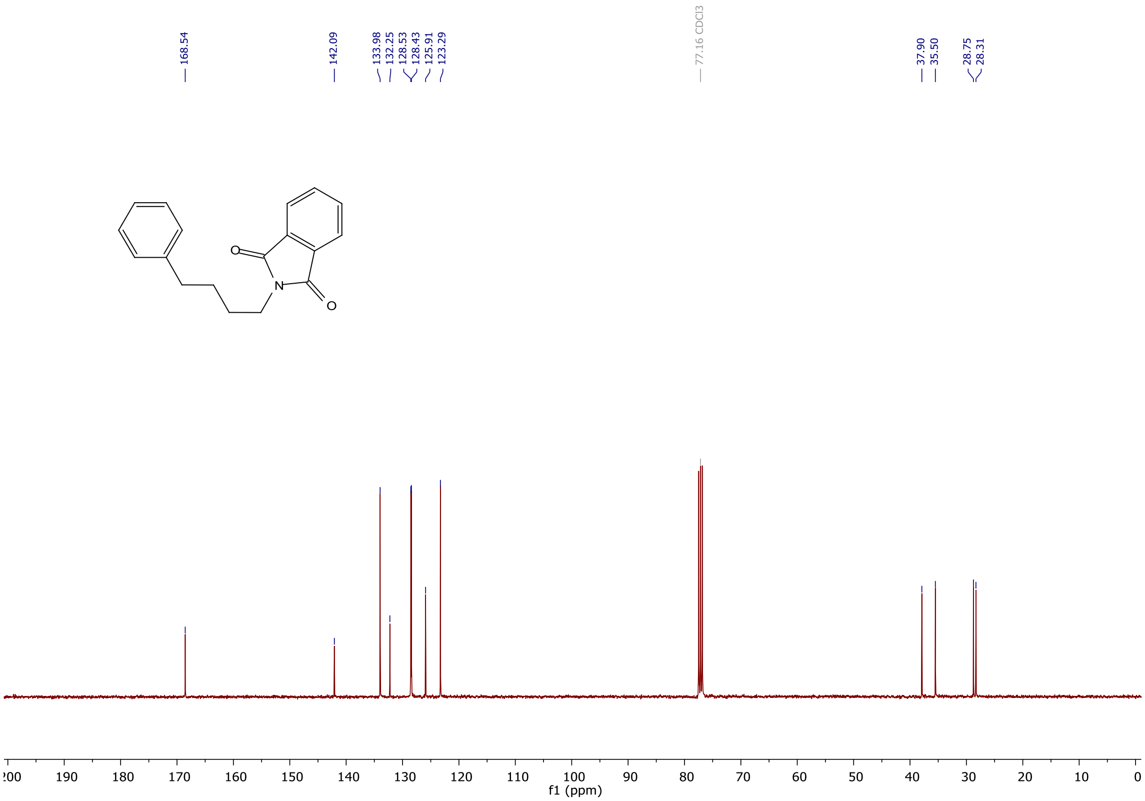

Supplementary Figure 78. ^{13}C NMR (101 MHz, $CDCl_3$) of compound **28**

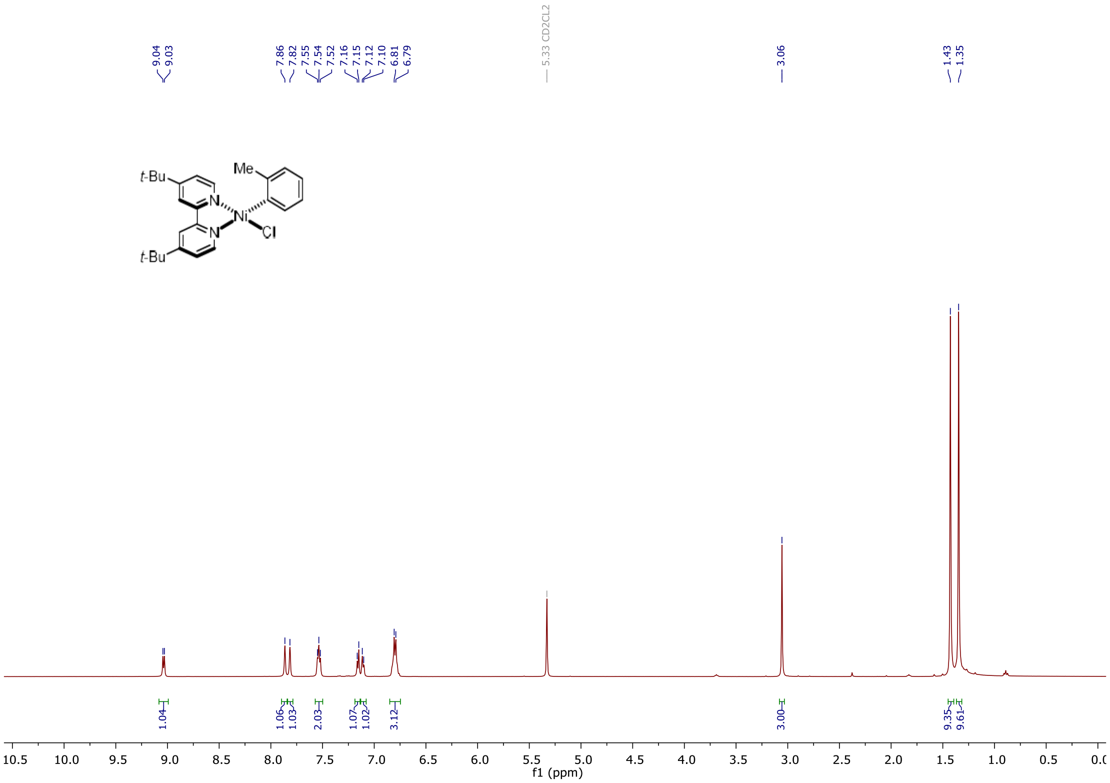

Supplementary Figure 79. ^{1}H NMR (400 MHz, CD_2Cl_2) of Ni(dtbbpy)(*o*-tolyl)chloride

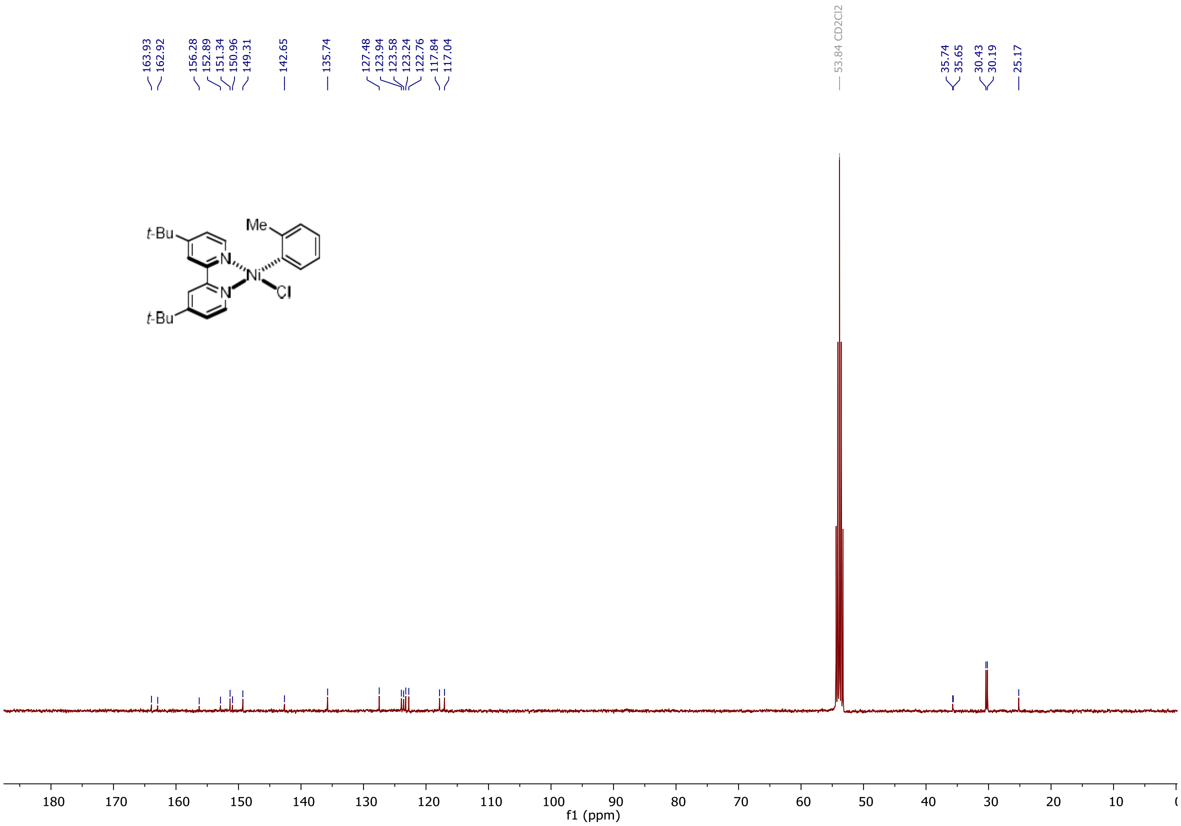

Supplementary Figure 80. ^{13}C NMR (101 MHz, CD_2Cl_2) of Ni(dtbbpy)(*o*-tolyl)chloride

Supplementary References

1. Lowry, M. S., et al. Single-layer electroluminescent devices and photoinduced hydrogen production from an ionic iridium(III) complex. *Chem. Mater.* **17**, 5712-5719 (2005).
2. Johnston, C. P., Smith, R. T., Allmendinger, S. & MacMillan, D. W. C. Metallaphotoredox-catalysed *sp*^{3}–*sp*^{3} cross-coupling of carboxylic acids with alkyl halides. *Nature* **536**, 322-325 (2016).
3. Zhang, L. et al. The combination of benzaldehyde and nickel-catalyzed photoredox C(sp^3)−H alkylation/arylation. *Angew. Chem. Int. Ed*. **58**, 1823 –1827 (2019).
4. Duffy, M. G. & Grayson. D. H. A novel competition between C–Se and C–Si cleavages in cyclization of β-seleno-β-silyl-substituted divinyl ketones. *J. Chem. Soc., Perkin Trans.* **1**, 1555-1563 (2002).
5. Le, C. et al. Selective *sp*^{3} C–H alkylation via polarity-match-based cross-coupling. *Nature* **547**, 79-83 (2017).
6. Santos, M. S., Corrêa, A. G., Paixão, M. W. & König. B. C(*sp*^{3})−C(*sp*^{3}) cross-coupling of alkyl bromides and ethers mediated by metal and visible light photoredox catalysis. *Adv. Synth. Catal*. **362**, 2367-2372 (2020).
7. Chow, Y. L. & Cheng. X. –E. The reaction pattern of norbornene with excited state carbonyl compounds: Photochemical preparations of norbornene derivatives. *Res. Chem. Intermed*. **19**, 211-234 (1993).
8. Cheng, H. et al. Bisphosphonium salt: an effective photocatalyst for the intramolecular hydroalkoxylation of olefins. *Science Bulletin* **64**, 1896–1901 (2019).
9. Shen, Y., Gu, Y. & Martin. R. *sp*^{3} C–H arylation and alkylation enabled by the synergy of triplet excited ketones and nickel catalysts. *J. Am. Chem. Soc.* **140**, 12200–12209 (2018).
10. Guo, S., AbuSalim, D. I. & Cook. S. P. Aqueous benzylic C–H trifluoromethylation for late-stage functionalization. *J. Am. Chem. Soc.* **140**, 12378–12382 (2018).
11. Shields, B. J., Kudisch, B., Scholes, G. D. & Doyle, A. G. Long-lived charge-transfer states of nickel(II) aryl halide complexes facilitate bimolecular photoinduced electron transfer. *J. Am. Chem. Soc*. **140**, 3035−3039 (2018).
12. Ting, S. I. *et al.* 3d-d Excited states of Ni(II) complexes relevant to photoredox catalysis: spectroscopic identification and mechanistic implications. *J. Am. Chem. Soc.* **142**, 5800-5810 (2020).
13. M. J. Frisch, et al. Gaussian 09, Revision D.01. Gaussian, Inc., Wallingford CT, 2013.
14. Zhao, Y. & Truhlar, D. G. The M06 suite of density functionals for main group thermochemistry, thermochemical kinetics, noncovalent interactions, excited states, and transition elements: two new functionals and systematic testing of four M06-class functionals and 12 other functionals. *Theor. Chem. Acc*. **120**, 215-241 (2008).
15. (a) Weigend, F. & Ahlrichs, R. Balanced basis sets of split valence, triple zeta valence and quadruple zeta valence quality for H to Rn: Design and assessment of accuracy. *Phys. Chem. Chem. Phys.* 7, 3297-3305 (2005). (b) Weigend, F. Accurate Coulomb-fitting basis sets for H to Rn. *Phys. Chem. Chem. Phys.* **8**, 1057-1065 (2006).
16. Marenich, A. V., Cramer, C. J. & Truhlar, D. G. Universal solvation model based on solute electron density and on a continuum model of the solvent defined by the bulk dielectric constant and atomic surface tensions. *J. Phys. Chem. B*, **113**, 6378-6396 (2009).
17. Reed, A. E., Curtiss, L. A. & Weinhold, F. Intermolecular interactions from a natural bond orbital, donor-acceptor viewpoint. *Chem. Rev*., **88**, 899–926 (1998).

18. Legault, C. Y. CYLview, 1.0b. Université de Sherbrooke, Sherbrooke, Canada, 2009. http://www.cylview.org.
19. Förster, T. Zwischenmolekulare energiewanderung und fluoreszenz. *Ann. Phys*. **437**, 55-75 (1948).
20. (a) Dexter, D. L. A theory of sensitized luminescence in solids. *J. Chem. Phys*. **21**, 836-850 (1953). (b) Turro, N. J. Modern molecular photochemistry, University science books, Sausalito, 1991.
21. Wigner, E. *Nachr. Akad. Wiss. Goettingen, Math.–Phys. Kl.* 375–381 (1927).
22. Ting, S. I. *et al.* 3d-d Excited states of Ni(II) complexes relevant to photoredox catalysis: spectroscopic identification and mechanistic implications. *J. Am. Chem. Soc.* **142**, 5800-5810 (2020).
