# Supplementary material for: Mechanistic insights into photochemical nickel-catalyzed cross-couplings enabled by energy transfer
Source: Nat Commun. 2022 May 18;13:2737. doi: 10.1038/s41467-022-30278-8 (PMC9117274; doi:10.1038/s41467-022-30278-8)
Supplement: Supplementary file 3 — Description of Additional Supplementary Files [file 41467_2022_30278_MOESM3_ESM.pdf]

Description of Additional Supplementary Files

File name: Supplementary Data 1

Description: Energies and Cartesian coordinates
