# Supplementary material for: Mechanistic insights into photochemical nickel-catalyzed cross-couplings enabled by energy transfer
Source: Nat Commun. 2022 May 18;13:2737. doi: 10.1038/s41467-022-30278-8 (PMC9117274; doi:10.1038/s41467-022-30278-8)
Supplement: Supplementary file 4 — Supplementary Data 1 [file 41467_2022_30278_MOESM4_ESM.docx]

**Energies and Cartesian coordinates**

Cartesian coordinates (Å) of the optimized structures of all intermediates and transition states at M06/Def2-TZVP(Ni,Ir)/Def2-SVP(non-metals) level of theory. *E*_e_ represents the absolute electronic energy in Hartree at M06(SMD)/Def2-TZVPP level of theory in THF solvent.

| **1_R_**  *E*_e_ : -2769.91564998  C -2.197904 -0.048393 0.029321  H -1.122479 -0.268277 0.107146  H -2.763010 -0.914595 0.410501  C -2.616002 0.304716 -1.368968  C -2.603575 -0.820086 -2.425999  C -4.110891 0.503150 -1.670243  H -2.034882 1.174494 -1.726957  C -3.893026 -0.220648 -3.008554  H -2.792227 -1.800875 -1.953602  H -1.719652 -0.914653 -3.076526  H -4.725332 -0.098399 -0.975854  H -4.507614 1.530445 -1.676665  H -4.674029 -0.912380 -3.360242  H -3.673873 0.491320 -3.821379  Br -2.552285 1.415472 1.268775  **THF**  *E*_e_ : -232.383775299  C 0.575305 -3.966673 2.161666  C -0.970754 -2.922319 0.785589  C -1.730776 -3.619847 1.901772  C -0.666353 -3.673369 2.987617  H 0.700586 -5.060013 2.014318  H 1.505704 -3.591750 2.622950  H -1.337944 -3.183152 -0.222441  H -2.651014 -3.094056 2.198775  H -2.012192 -4.643035 1.595074  H -0.577072 -2.689013 3.480798  H -0.858068 -4.423755 3.769680  O 0.374526 -3.329139 0.918173  H -1.047991 -1.819316 0.886771  **1**  *E*_e_ : -427.635453324  C -3.080605 -2.533763 0.121067  H -3.336925 -2.893503 -0.895847  H -3.809121 -2.992166 0.817489  C -3.191048 -1.027717 0.164939  C -2.400058 -0.192244 -0.860322  C -4.471621 -0.347122 -0.349277  H -2.957016 -0.677131 1.186470  C -3.592349 0.775466 -0.922403  H -2.287180 -0.749933 -1.809113  H -1.411775 0.200808 -0.570647  H -4.931002 -0.947211 -1.156016  H -5.258817 -0.085420 0.376428  H -3.864946 1.208680 -1.897851  H -3.475298 1.600992 -0.200644  C -0.653475 -3.641931 2.467836  C -1.700182 -3.036487 0.474822  C -1.528211 -4.546948 0.478737  C -0.353543 -4.713921 1.428867  H 0.258722 -3.219664 2.926415  H -1.272230 -4.050792 3.292444  H -0.965055 -2.589083 -0.235863  H -2.437131 -5.019848 0.896291  H -1.360171 -4.965381 -0.525833  H -0.261565 -5.722122 1.860722  H 0.591091 -4.488912 0.903143  O -1.368290 -2.627788 1.794702  **A^S^**  *E*_e_ : -2544.47713790  Ni -1.378223 2.577503 0.010561  C -2.896310 2.628806 -1.369569  C -3.393273 2.273836 -0.112147  C -1.496831 4.616469 0.042383  C -4.133880 3.220141 0.812199  C -1.805153 4.109145 1.307797  H -2.718468 1.817744 -2.089442  H -4.663335 3.985036 0.217553  H -3.547725 1.199967 0.069219  H -0.469323 4.981023 -0.103365  H -4.932042 2.674150 1.345119  H -1.011516 4.130487 2.067624  C -2.938802 4.018336 -1.955967  H -2.242180 4.038818 -2.815314  H -3.934109 4.250157 -2.390670  C -2.527749 5.108413 -0.954050  H -3.413051 5.492145 -0.417404  H -2.131016 5.980298 -1.503136  C -3.201300 3.887360 1.835163  H -3.121130 3.236887 2.726686  H -3.652896 4.829796 2.210834  C 0.455988 0.530861 0.709180  C 1.047004 -0.542214 1.374328  C 0.432451 -1.081125 2.501545  H 1.987576 -0.983088 1.036637  C -1.298885 0.547364 2.209018  C -0.778709 -0.514842 2.930871  H -2.244927 1.007834 2.517935  H -1.317466 -0.882137 3.806067  C 1.029477 1.161984 -0.479903  C 2.235759 0.766580 -1.055510  C 0.756052 2.803368 -2.079978  C 2.709094 1.421398 -2.189437  H 2.832777 -0.049076 -0.641703  C 1.941433 2.472589 -2.716834  H 0.136655 3.621720 -2.466256  H 2.246626 3.031463 -3.603287  N -0.717445 1.076141 1.125711  N 0.288584 2.180765 -0.991450  O 1.050168 -2.114553 3.098499  O 3.877651 0.996011 -2.698811  C 4.389637 1.630732 -3.837651  H 4.584609 2.703911 -3.658436  H 5.339681 1.137917 -4.081597  H 3.709648 1.534412 -4.703741  C 0.463633 -2.690046 4.232772  H -0.537831 -3.103323 4.013507  H 1.119012 -3.510285 4.553220  H 0.373730 -1.965581 5.062719  **A^T^**  *E*_e_ : -2544.46657343  Ni -1.372188 2.569396 0.010113  C -3.176983 2.455247 -1.101712  C -3.542844 2.353117 0.218738  C -1.582704 4.727288 -0.299538  C -4.058028 3.476144 1.082128  C -1.676317 4.403218 1.032640  H -3.010215 1.512471 -1.640527  H -4.642021 4.184609 0.472513  H -3.626504 1.338134 0.626267  H -0.585554 4.997231 -0.668467  H -4.771416 3.071127 1.818969  H -0.747698 4.452097 1.617634  C -3.192952 3.693459 -1.956685  H -2.518006 3.511080 -2.811695  H -4.197222 3.842902 -2.401310  C -2.740819 4.970588 -1.233305  H -3.580894 5.426080 -0.684239  H -2.448751 5.719541 -1.988060  C -2.934611 4.205593 1.833919  H -2.665204 3.618205 2.729550  H -3.303641 5.176998 2.219612  C 0.453473 0.491163 0.660396  C 1.049893 -0.593829 1.356355  C 0.393501 -1.191953 2.411030  H 2.041826 -0.963802 1.088093  C -1.405785 0.346084 2.068410  C -0.886175 -0.712533 2.797668  H -2.395360 0.740425 2.339715  H -1.459997 -1.141023 3.619450  C 1.064754 1.175697 -0.425904  C 2.286835 0.778460 -1.030274  C 0.917927 2.952894 -1.936251  C 2.803019 1.488951 -2.092944  H 2.831219 -0.103028 -0.685060  C 2.101094 2.623759 -2.579785  H 0.349418 3.826341 -2.286050  H 2.454801 3.230508 -3.413547  N -0.803229 0.936991 1.041253  N 0.399262 2.294430 -0.904079  O 1.023297 -2.212471 3.029248  O 3.963440 1.050287 -2.623411  C 4.514848 1.733238 -3.710816  H 4.764434 2.781265 -3.461857  H 5.441894 1.211128 -3.983979  H 3.841509 1.732577 -4.587922  C 0.399878 -2.843616 4.108955  H -0.559449 -3.311819 3.820751  H 1.079922 -3.632267 4.458578  H 0.211988 -2.147081 4.946921  **cod**  *E*_e_ : -311.898164175  C -3.158036 2.533834 -1.306422  C -3.751192 2.331461 -0.123718  C -1.607560 4.948095 0.035624  C -4.101164 3.324074 0.950362  C -1.750754 4.376030 1.237218  H -2.995786 1.637054 -1.920446  H -4.548399 4.238277 0.531149  H -3.992633 1.286921 0.116873  H -0.618322 5.378083 -0.173603  H -4.890431 2.886510 1.584694  H -0.871329 4.416826 1.894883  C -2.673912 3.793365 -1.953981  H -1.670167 3.588970 -2.375335  H -3.305272 3.996837 -2.841851  C -2.593942 5.053856 -1.094593  H -3.596804 5.332018 -0.736248  H -2.284996 5.886243 -1.749153  C -2.924232 3.682907 1.855893  H -2.551786 2.756307 2.334916  H -3.289558 4.298498 2.702010  **B^T^**  *E*_e_ : -5002.46065657  Ni -0.490646 -0.817339 0.825210  C 1.201448 1.290965 0.142613  C -0.895162 2.151626 0.727628  C -0.522392 3.464612 0.483123  C 0.807676 3.683832 0.034869  C 1.654979 2.608108 -0.130677  C 1.996892 0.117109 0.024270  C 2.065914 -2.212632 0.260767  C 3.392211 -2.299411 -0.128585  C 4.050796 -1.084521 -0.457868  C 3.356568 0.104306 -0.380302  H -1.912392 1.936656 1.085122  H -1.236677 4.272505 0.643227  H 1.523522 -3.129350 0.526119  H 3.882468 -3.272193 -0.169118  N -0.101992 1.096447 0.569528  N 1.370228 -1.079742 0.344855  Br -2.724579 -1.385375 1.317867  O 5.341246 -1.020950 -0.849054  O 1.305227 4.907666 -0.243997  C 0.488587 6.028589 -0.075008  H -0.404765 5.996048 -0.726086  H 0.154092 6.146739 0.972327  H 1.086383 6.908255 -0.349922  C 6.086447 -2.200362 -0.927649  H 6.162540 -2.711090 0.050144  H 5.665424 -2.911654 -1.662267  H 7.097843 -1.922359 -1.253982  H 3.881096 1.028325 -0.633487  H 2.674980 2.800160 -0.471013  C -3.638000 -1.416554 -0.446780  H -3.614929 -2.473453 -0.756267  H -4.673339 -1.128620 -0.209292  C -2.999384 -0.513687 -1.457648  C -1.674992 -0.920699 -2.129807  C -3.653538 -0.513889 -2.858670  H -2.931205 0.517615 -1.065825  C -2.246034 -0.412081 -3.463952  H -1.556211 -2.019862 -2.124569  H -0.744138 -0.472193 -1.745632  H -4.117287 -1.493199 -3.074779  H -4.390379 0.273811 -3.082161  H -2.033390 -0.992680 -4.374343  H -1.959739 0.635843 -3.648445  **[B-C]^T^**  *E*_e_ : -5002.44995593  Ni -0.298377 -0.292768 1.322542  C 1.383051 1.848177 0.576535  C -0.706019 2.671059 1.176172  C -0.346318 3.985921 0.915860  C 0.966215 4.224524 0.454063  C 1.827929 3.148776 0.288390  C 2.198559 0.659291 0.458335  C 2.265105 -1.652412 0.694679  C 3.590739 -1.730435 0.299055  C 4.250257 -0.524618 -0.024488  C 3.544085 0.666702 0.060435  H -1.713258 2.435830 1.543940  H -1.070007 4.786160 1.074430  H 1.723393 -2.567861 0.960614  H 4.081635 -2.702983 0.250519  N 0.107639 1.630623 1.017024  N 1.570394 -0.517236 0.781507  Br -2.453085 -0.915127 1.847468  O 5.534305 -0.448149 -0.413200  O 1.450538 5.442794 0.159065  C 0.623409 6.562136 0.316967  H -0.271710 6.508202 -0.328962  H 0.296656 6.689033 1.365005  H 1.213045 7.441030 0.025218  C 6.288367 -1.624794 -0.507004  H 6.367006 -2.142783 0.466089  H 5.866695 -2.327068 -1.248905  H 7.296262 -1.335736 -0.832248  H 4.062798 1.595577 -0.186576  H 2.841628 3.347124 -0.066489  C -3.632228 -0.817269 -0.168208  H -3.686618 -1.886899 -0.414441  H -4.576214 -0.409021 0.214463  C -2.857611 0.031389 -1.100225  C -1.552419 -0.500628 -1.722489  C -3.396773 0.209891 -2.548715  H -2.677811 1.029913 -0.655755  C -1.944030 0.178489 -3.043349  H -1.586285 -1.602691 -1.800640  H -0.601353 -0.217567 -1.238158  H -3.951482 -0.690375 -2.866746  H -4.018048 1.094808 -2.762208  H -1.728303 -0.348346 -3.986073  H -1.520071 1.194211 -3.111779  **C^T^**  *E*_e_ : -5002.48851874  Ni -0.544410 -1.228416 1.168101  C 0.905245 1.063432 0.279054  C -1.162037 1.686967 1.094219  C -0.968582 3.017643 0.734608  C 0.230699 3.363084 0.102794  C 1.184375 2.360495 -0.123792  C 1.831885 -0.075142 0.100030  C 2.111116 -2.346910 0.379281  C 3.401746 -2.316028 -0.135975  C 3.916580 -1.083595 -0.553915  C 3.106497 0.051919 -0.429255  H -2.085639 1.361041 1.586574  H -1.750163 3.749264 0.945355  H 1.671734 -3.293673 0.712046  H 3.974278 -3.242323 -0.202664  N -0.257619 0.736144 0.875006  N 1.337420 -1.269131 0.501041  Br -2.501726 -2.210408 1.798059  O 5.135257 -0.897768 -1.068599  O 0.552097 4.594173 -0.306836  C -0.366043 5.639697 -0.112746  H -1.315615 5.451076 -0.644123  H -0.580481 5.799517 0.958771  H 0.093606 6.548462 -0.520889  C 5.990554 -2.003064 -1.214431  H 6.214887 -2.474609 -0.241287  H 5.561139 -2.762933 -1.891066  H 6.925160 -1.628694 -1.650164  H 3.508724 1.014816 -0.751226  H 2.119704 2.634681 -0.616433  C -3.156468 0.574076 -0.882308  H -3.539677 -0.244710 -0.259962  H -3.629872 1.558956 -0.787541  C -2.261091 0.288121 -2.018050  C -1.513839 -1.058657 -2.077492  C -2.874139 -0.081037 -3.405316  H -1.544911 1.123277 -2.174437  C -1.725300 -1.079286 -3.598241  H -2.102064 -1.839478 -1.563418  H -0.485968 -1.102099 -1.678118  H -3.838450 -0.599080 -3.264678  H -3.013695 0.725734 -4.143510  H -1.950218 -2.043054 -4.081894  H -0.876855 -0.616554 -4.131263  **D^T^**  *E*_e_ : -5002.51626725  Ni -0.550349 -0.610149 1.081115  C 1.208913 1.582145 0.383661  C -0.919776 2.390647 0.746346  C -0.588946 3.657702 0.283107  C 0.724144 3.877072 -0.152541  C 1.631368 2.812480 -0.099122  C 2.082251 0.390567 0.440992  C 2.142476 -1.882041 0.861434  C 3.498911 -1.960133 0.570694  C 4.164603 -0.784742 0.199420  C 3.436314 0.410678 0.140974  H -1.941025 2.174186 1.081601  H -1.349236 4.439862 0.265632  H 1.568994 -2.763392 1.174340  H 4.008577 -2.921828 0.643654  N -0.057061 1.380727 0.804956  N 1.453760 -0.746975 0.791208  Br -1.097730 -2.262939 2.643026  O 5.462551 -0.707281 -0.103023  O 1.186543 5.035301 -0.628464  C 0.316506 6.135893 -0.716600  H -0.536596 5.926253 -1.385647  H -0.068160 6.430086 0.275828  H 0.896737 6.968752 -1.132552  C 6.245989 -1.874047 -0.064701  H 6.271380 -2.313225 0.948078  H 5.878271 -2.633350 -0.777020  H 7.264474 -1.583579 -0.350582  H 3.962373 1.326064 -0.137742  H 2.649527 2.980344 -0.456238  C -1.477331 -0.784195 -0.690007  H -1.719263 -1.860827 -0.781784  H -2.432889 -0.238132 -0.542489  C -0.750848 -0.296127 -1.910792  C 0.529158 -1.029115 -2.371456  C -1.327650 -0.577956 -3.324662  H -0.533307 0.791989 -1.842720  C 0.104768 -0.799440 -3.828791  H 0.465397 -2.097657 -2.092545  H 1.513481 -0.646811 -2.044982  H -1.900552 -1.522280 -3.311760  H -1.947192 0.196643 -3.807234  H 0.285599 -1.603445 -4.560835  H 0.539716 0.132148 -4.230394  **D^S^**  *E*_e_ : -5002.52899504  Ni -0.318366 -0.358259 0.232937  C 1.494524 1.813369 0.316865  C -0.664197 2.593650 0.189776  C -0.253127 3.919827 0.185285  C 1.115757 4.191821 0.252927  C 1.994498 3.105940 0.320287  C 2.365291 0.626332 0.382690  C 2.413201 -1.674092 0.409403  C 3.800873 -1.702103 0.492524  C 4.490728 -0.486765 0.521864  C 3.747638 0.698797 0.464934  H -1.731337 2.361891 0.143768  H -1.006075 4.707391 0.130346  H 1.817606 -2.596109 0.380613  H 4.311399 -2.665601 0.532522  N 0.165137 1.549405 0.252121  N 1.705226 -0.547635 0.355709  Br -0.706623 -2.633197 0.185552  O 5.817537 -0.358359 0.600384  O 1.658803 5.409947 0.259007  C 0.815322 6.533228 0.192559  H 0.225433 6.542556 -0.740717  H 0.126357 6.574297 1.054390  H 1.461685 7.419271 0.212496  C 6.612571 -1.516411 0.657793  H 6.381253 -2.124057 1.550244  H 6.486172 -2.140869 -0.244019  H 7.656520 -1.184256 0.715753  H 4.286773 1.647643 0.487633  H 3.065447 3.309423 0.372845  C -2.214928 -0.037214 0.096273  H -2.788168 -0.912186 0.444126  H -2.451561 0.788538 0.797951  C -2.610172 0.288501 -1.321886  C -2.589703 -0.822397 -2.389421  C -4.105253 0.499502 -1.675294  H -2.041856 1.157225 -1.718158  C -3.839872 -0.191559 -3.020495  H -2.814235 -1.795390 -1.918982  H -1.679410 -0.946308 -2.999334  H -4.736090 -0.127298 -1.019761  H -4.512672 1.525596 -1.672020  H -4.616670 -0.858941 -3.428006  H -3.573746 0.541754 -3.801769  **E^D^**  *E*_e_ : -2428.38573911  Ni -0.399848 -0.111648 0.330315  C 1.572827 2.019500 0.341289  C -0.552782 2.903840 0.279097  C -0.076273 4.210014 0.228709  C 1.309725 4.406054 0.231586  C 2.144362 3.282914 0.290670  C 2.350258 0.764953 0.413628  C 2.225413 -1.531066 0.527619  C 3.608098 -1.669549 0.563066  C 4.388899 -0.509210 0.516053  C 3.736470 0.726344 0.439728  H -1.630063 2.697638 0.276078  H -0.783629 5.039741 0.186968  H 1.582047 -2.417620 0.557650  H 4.045968 -2.667092 0.624019  N 0.238600 1.838333 0.330874  N 1.597707 -0.357918 0.454416  O 5.725980 -0.483305 0.538615  O 1.913761 5.598451 0.182917  C 1.126512 6.759709 0.120024  H 0.494021 6.774686 -0.785316  H 0.480269 6.862901 1.009787  H 1.816294 7.612201 0.083542  C 6.429427 -1.696158 0.615287  H 6.188521 -2.249331 1.540534  H 6.219890 -2.344513 -0.254104  H 7.497896 -1.446831 0.620745  H 4.344897 1.632623 0.403630  H 3.225245 3.440078 0.295230  C -2.330031 -0.422246 0.118802  H -2.533753 -1.515212 0.163553  H -2.878344 0.010737 0.982035  C -2.900406 0.109576 -1.176999  C -2.369665 -0.443584 -2.512990  C -4.321007 -0.278528 -1.669651  H -2.824163 1.218559 -1.214634  C -3.778831 -0.289832 -3.106075  H -2.102291 -1.509780 -2.387899  H -1.519368 0.068503 -2.997665  H -4.565157 -1.300470 -1.325571  H -5.172639 0.380804 -1.427622  H -4.145094 -1.062105 -3.803306  H -3.904534 0.694321 -3.590879  **[E-F]^T^**  *E*_e_ : -5234.81644361  Ni -1.837557 -3.806151 -1.271678  C -2.824344 -1.098702 -0.733703  C -2.849951 -1.525513 -3.001824  C -3.278581 -0.239739 -3.306430  C -3.472051 0.656353 -2.247192  C -3.238975 0.210009 -0.941343  C -2.575017 -1.671568 0.606268  C -1.786155 -3.479216 1.794154  C -2.093911 -2.890675 3.014451  C -2.676451 -1.619602 3.008939  C -2.919131 -1.006920 1.774722  H -2.672896 -2.261307 -3.795376  H -3.445865 0.039383 -4.347671  H -1.340948 -4.480204 1.761734  H -1.883129 -3.434232 3.936494  N -2.626031 -1.947463 -1.761098  N -2.004589 -2.894897 0.617078  O -3.031623 -0.934116 4.099707  O -3.865605 1.925287 -2.384273  C -4.109610 2.431659 -3.672275  H -3.203473 2.394258 -4.302346  H -4.922304 1.881503 -4.178547  H -4.413454 3.478822 -3.551150  C -2.826877 -1.512330 5.363833  H -3.389023 -2.456326 5.474829  H -1.756642 -1.708288 5.553223  H -3.191889 -0.792758 6.107275  H -3.394277 -0.023900 1.768829  H -3.384022 0.914833 -0.120210  C -1.043419 -4.371187 -3.007554  H -0.728171 -5.429903 -2.912238  H -1.792832 -4.336863 -3.825403  C 0.156590 -3.506832 -3.306071  C 1.271927 -3.418824 -2.244734  C 1.255976 -3.935314 -4.315744  H -0.157067 -2.471987 -3.564072  C 2.302896 -3.314348 -3.379450  H 1.351804 -4.385441 -1.712914  H 1.216501 -2.614221 -1.488241  H 1.336164 -5.037088 -4.332556  H 1.193935 -3.575326 -5.356909  H 3.270386 -3.828175 -3.257333  H 2.504836 -2.261674 -3.643356  C -4.671138 -6.520217 0.250302  C -3.568828 -4.960496 -1.090925  C -3.873607 -6.188885 -1.933957  C -5.055850 -6.805155 -1.192183  H -3.985560 -7.307375 0.633269  H -5.535988 -6.473652 0.936427  H -1.948700 -5.635802 -0.545295  H -4.088535 -5.923505 -2.981402  H -3.028818 -6.904648 -1.952076  H -5.989723 -6.272941 -1.448623  H -5.209275 -7.876872 -1.395716  O -4.021021 -5.268495 0.235145  Br -0.855975 -6.511169 0.066322  H -4.179806 -4.106376 -1.459644  **F^T^**  *E*_e_ : -2660.13692348  Ni -1.053766 -1.168206 0.418210  C 0.112438 1.100679 1.728312  C -2.166985 0.978845 2.189732  C -2.149080 2.135512 2.945020  C -0.917271 2.821705 3.074445  C 0.207788 2.294654 2.460860  C 1.220186 0.439571 1.086381  C 1.848436 -1.360670 -0.251403  C 3.187788 -1.029179 -0.192096  C 3.554435 0.112108 0.561143  C 2.561778 0.836119 1.202670  H -3.104720 0.425023 2.054922  H -3.069821 2.488624 3.410919  H 1.526783 -2.252998 -0.801089  H 3.918730 -1.647111 -0.714923  N -1.092771 0.457818 1.588790  N 0.875812 -0.661984 0.345156  O 4.820118 0.541531 0.709122  O -0.763227 3.968271 3.760069  C -1.876302 4.543315 4.385679  H -2.668381 4.804555 3.660454  H -2.307337 3.878146 5.156012  H -1.530922 5.464306 4.873307  C 5.857260 -0.166567 0.089345  H 5.921532 -1.208265 0.453194  H 5.747823 -0.181944 -1.010289  H 6.793310 0.349500 0.339997  H 2.853924 1.701379 1.802202  H 1.153605 2.832749 2.557929  C -2.874685 -1.462121 -0.243659  H -2.788862 -2.292197 -0.976587  H -3.567044 -1.799614 0.551317  C -3.337924 -0.208344 -0.938844  C -2.431776 0.420538 -2.017323  C -4.460792 -0.244061 -2.009357  H -3.592598 0.578052 -0.200200  C -3.694160 0.833997 -2.789372  H -1.879245 -0.375228 -2.552003  H -1.706804 1.194529 -1.708190  H -4.443107 -1.212597 -2.541009  H -5.498099 -0.039241 -1.695757  H -3.678564 0.776133 -3.889722  H -4.023941 1.847462 -2.503848  C 0.819918 -3.021345 2.651190  C -1.097001 -2.918897 1.366473  C -1.546870 -2.747914 2.801332  C -0.252137 -2.312621 3.480844  H 1.216631 -3.923438 3.152376  H 1.681825 -2.361527 2.429084  H -1.704771 -3.600199 0.749055  H -2.374757 -2.033271 2.926782  H -1.886473 -3.722197 3.207812  H -0.136030 -1.217829 3.413829  H -0.207404 -2.584307 4.546628  O 0.203050 -3.429128 1.442240  **[F-G]^T^**  *E*_e_ : -2660.11049499  Ni -0.959938 -1.164067 0.801991  C 0.095439 1.248477 1.861891  C -2.160354 1.107057 2.451165  C -2.145463 2.297488 3.163712  C -0.911010 2.998380 3.213763  C 0.190874 2.477005 2.569442  C 1.175933 0.634633 1.169434  C 1.853179 -1.200248 -0.122335  C 3.149477 -0.730938 -0.269633  C 3.460591 0.506671 0.356224  C 2.485091 1.176339 1.064911  H -3.101396 0.539190 2.382441  H -3.054231 2.654901 3.648217  H 1.562784 -2.153436 -0.587155  H 3.874437 -1.305887 -0.845834  N -1.117444 0.578006 1.821611  N 0.896130 -0.577461 0.559860  O 4.680867 1.081224 0.296651  O -0.746742 4.168115 3.867704  C -1.838593 4.736406 4.528755  H -2.668079 4.972697 3.836718  H -2.227676 4.081779 5.330627  H -1.488942 5.672867 4.984297  C 5.699014 0.443808 -0.417255  H 5.938652 -0.552702 -0.002083  H 5.446833 0.325450 -1.487331  H 6.593413 1.076779 -0.339049  H 2.746519 2.128807 1.531207  H 1.130635 3.031362 2.620941  C -2.837161 -1.741331 0.065302  H -2.851470 -2.571146 -0.663763  H -3.802315 -1.761160 0.603156  C -2.797896 -0.442064 -0.749299  C -1.718594 -0.195510 -1.822249  C -3.856091 -0.357742 -1.880719  H -2.889586 0.446801 -0.104164  C -2.812939 0.371716 -2.739549  H -1.320559 -1.154539 -2.203227  H -0.865448 0.448482 -1.553456  H -4.078144 -1.362749 -2.282586  H -4.811217 0.146328 -1.659707  H -2.769279 0.131367 -3.813423  H -2.896627 1.465915 -2.635385  C 0.022478 -3.698990 2.119089  C -2.011658 -2.822504 1.387184  C -1.928417 -2.587199 2.900775  C -0.441478 -2.748972 3.210381  H -0.121361 -4.757028 2.413231  H 1.075436 -3.559779 1.827896  H -2.830976 -3.525185 1.169370  H -2.330439 -1.611627 3.210606  H -2.520608 -3.364646 3.418471  H 0.083514 -1.782168 3.130591  H -0.257633 -3.151067 4.218121  O -0.791990 -3.425787 0.979630  **G^T^**  *E*_e_ : -2660.18921633  Ni -0.787468 -0.699060 2.031187  C 0.353169 1.824945 1.934165  C -1.849375 1.975931 2.716039  C -1.808031 3.357495 2.831712  C -0.589785 3.996590 2.479049  C 0.472502 3.236183 2.038508  C 1.385990 0.960918 1.475989  C 1.995418 -1.245222 1.004555  C 3.272084 -0.900563 0.581915  C 3.609239 0.478274 0.624949  C 2.676020 1.392924 1.065543  H -2.771701 1.442719 2.985901  H -2.684404 3.899670 3.187199  H 1.695256 -2.304118 0.987763  H 3.961919 -1.672445 0.240111  N -0.841370 1.216241 2.295031  N 1.078724 -0.389871 1.438696  O 4.815161 0.959901 0.251080  O -0.404180 5.332846 2.549628  C -1.451836 6.140798 2.997284  H -2.341308 6.071471 2.343696  H -1.757571 5.893375 4.030879  H -1.089954 7.177990 2.981395  C 5.792966 0.068670 -0.195461  H 6.067024 -0.672513 0.578369  H 5.477593 -0.476451 -1.104715  H 6.684954 0.662046 -0.438945  H 2.956902 2.448273 1.088201  H 1.399412 3.748237 1.770873  C -3.067599 -2.517336 0.149926  H -3.348623 -2.900325 -0.851349  H -3.776174 -2.968003 0.871415  C -3.191974 -1.009865 0.156710  C -2.409532 -0.175406 -0.875616  C -4.481397 -0.350803 -0.363444  H -2.972264 -0.616036 1.173316  C -3.611872 0.779495 -0.937163  H -2.293067 -0.739098 -1.820615  H -1.425972 0.227745 -0.577710  H -4.928797 -0.961936 -1.168842  H -5.274124 -0.099688 0.360069  H -3.889868 1.209479 -1.911915  H -3.496617 1.605092 -0.214913  C -0.634845 -3.689042 2.490079  C -1.679904 -3.030241 0.456811  C -1.523024 -4.539516 0.480244  C -0.351026 -4.734111 1.429466  H 0.263244 -3.286093 2.985941  H -1.319564 -4.069815 3.270195  H -0.949347 -2.578946 -0.249031  H -2.438983 -5.002601 0.892078  H -1.357765 -4.957156 -0.524308  H -0.273295 -5.750422 1.842702  H 0.598451 -4.507395 0.913147  O -1.283863 -2.621117 1.789364  **B^S^**  *E*_e_ : -5002.44458201  Ni -0.202050 -1.054179 0.037475  C 1.507073 1.191577 -0.138060  C -0.662794 1.910414 -0.388407  C -0.291870 3.249761 -0.357605  C 1.066319 3.557752 -0.214949  C 1.971806 2.499873 -0.101373  C 2.388831 0.022838 0.007307  C 2.483302 -2.238397 0.433983  C 3.864575 -2.271020 0.287411  C 4.530822 -1.076319 -0.012847  C 3.766901 0.084828 -0.154249  H -1.718398 1.644688 -0.516918  H -1.059854 4.019338 -0.454074  H 1.937873 -3.154567 0.682131  H 4.392070 -3.217601 0.418125  N 0.192307 0.888590 -0.285901  N 1.740215 -1.133096 0.300214  Br -1.794451 -2.600418 0.073185  O 5.857286 -0.955917 -0.183854  O 1.573653 4.799492 -0.169045  C 0.698985 5.890490 -0.261082  H 0.153869 5.899860 -1.222437  H -0.036792 5.897155 0.563586  H 1.310084 6.799841 -0.194886  C 6.659227 -2.098887 -0.067600  H 6.596251 -2.544240 0.941994  H 6.386479 -2.869152 -0.811873  H 7.695410 -1.785472 -0.249129  H 4.277284 1.015551 -0.413657  H 3.029891 2.733336 0.039359  C -3.601368 -2.028992 -0.701435  H -3.859897 -2.814157 -1.431003  H -4.301575 -2.060549 0.147470  C -3.475233 -0.678058 -1.325832  C -2.641094 -0.514291 -2.608445  C -4.676955 -0.087693 -2.097279  H -3.124282 0.041955 -0.559648  C -3.675415 0.512122 -3.097129  H -2.676434 -1.443028 -3.206763  H -1.584947 -0.217130 -2.497860  H -5.271834 -0.893649 -2.562950  H -5.366272 0.584398 -1.560323  H -3.944918 0.519514 -4.165210  H -3.388682 1.538760 -2.809933  **[B-C]^S^**  *E*_e_ : -5001.08119173  Ni -0.513949 -0.807329 0.749762  C 1.213756 1.547471 -0.043484  C -0.820922 2.575051 -0.220197  C -0.299411 3.829281 0.093964  C 1.074803 3.918704 0.342261  C 1.840987 2.749573 0.269858  C 1.968707 0.271804 -0.085345  C 1.983318 -2.010316 0.272118  C 3.298713 -2.134344 -0.153620  C 3.981692 -0.978753 -0.549633  C 3.291934 0.235520 -0.508120  H -1.897044 2.473748 -0.420712  H -0.959798 4.697520 0.137422  H 1.434016 -2.890953 0.616833  H 3.764948 -3.121120 -0.155877  N -0.102678 1.463664 -0.294423  N 1.308314 -0.848713 0.315191  Br -2.712008 -0.757411 1.273952  O 5.253928 -0.943396 -0.977344  O 1.725454 5.048133 0.663851  C 0.996329 6.239310 0.774454  H 0.512279 6.515207 -0.180103  H 0.220129 6.173781 1.558525  H 1.708126 7.028714 1.048605  C 5.976145 -2.142010 -1.043553  H 6.071831 -2.619408 -0.051314  H 5.511299 -2.863629 -1.739930  H 6.979854 -1.893998 -1.412263  H 3.803117 1.141087 -0.843910  H 2.907406 2.806202 0.503186  C -3.621413 -1.543241 -0.311109  H -3.307841 -2.599251 -0.328176  H -4.694314 -1.482760 -0.070283  C -3.267968 -0.829638 -1.581169  C -1.872702 -1.016234 -2.202634  C -3.868815 -1.386912 -2.891420  H -3.503693 0.247215 -1.485845  C -2.542651 -1.048589 -3.587800  H -1.460342 -2.003541 -1.924111  H -1.108026 -0.254951 -1.978903  H -4.016510 -2.479365 -2.815930  H -4.801815 -0.937291 -3.268290  H -2.157039 -1.757319 -4.337328  H -2.572749 -0.046187 -4.046670  **C^S^**  *E*_e_ : -2428.24023340  Ni 0.221903 -0.427529 -0.576565  C 1.746255 1.830089 -0.218632  C -0.276802 2.333091 -1.241406  C 0.030554 3.682498 -1.310986  C 1.263806 4.116565 -0.804090  C 2.127658 3.155798 -0.249826  C 2.580329 0.746595 0.335436  C 2.686380 -1.528409 0.727408  C 3.946266 -1.432639 1.299971  C 4.541793 -0.166029 1.387464  C 3.830229 0.940114 0.889087  H -1.229689 1.963141 -1.627559  H -0.690168 4.368998 -1.757089  H 2.195393 -2.503969 0.645276  H 4.439508 -2.334352 1.665135  N 0.547102 1.421715 -0.711528  N 2.011730 -0.479270 0.255456  O 5.730888 0.081676 1.905355  O 1.689936 5.365241 -0.803386  C 0.878050 6.390102 -1.345915  H 0.677210 6.212598 -2.415495  H -0.074387 6.474202 -0.796681  H 1.439014 7.325027 -1.236440  C 6.508601 -0.979045 2.427512  H 5.990230 -1.477159 3.263711  H 6.748065 -1.718347 1.645127  H 7.439104 -0.533355 2.796743  H 4.294504 1.925969 0.957270  H 3.089444 3.490165 0.143928  C -1.405097 -0.845517 -1.263814  H -1.474918 -0.913779 -2.366619  H -0.899508 -1.822631 -0.863589  C -2.728306 -0.586285 -0.629246  C -3.549047 0.620617 -1.140123  C -3.940134 -1.477361 -1.016344  H -2.641706 -0.543886 0.471780  C -4.822358 -0.222558 -0.983670  H -3.328825 0.816496 -2.206413  H -3.472798 1.568970 -0.582711  H -3.819903 -1.862757 -2.043841  H -4.186476 -2.321101 -0.353617  H -5.608690 -0.120411 -1.745753  H -5.281485 -0.075791 0.007010  **F^S^**  *E*_e_ : -2660.16951434  Ni -0.983682 -0.944499 0.932678  C 0.183051 1.491440 1.934953  C -2.103462 1.617169 2.092655  C -2.054414 2.821605 2.784606  C -0.800745 3.374577 3.063195  C 0.334830 2.686307 2.623697  C 1.320424 0.705996 1.424787  C 1.976396 -1.263506 0.436957  C 3.294820 -0.833494 0.325237  C 3.629241 0.432885 0.809918  C 2.609672 1.213004 1.366108  H -3.071140 1.158380 1.865038  H -2.986441 3.298176 3.092350  H 1.662329 -2.257928 0.097157  H 4.029626 -1.495819 -0.135488  N -1.027261 0.954642 1.671391  N 1.001025 -0.531250 0.982676  O 4.851750 0.973499 0.776498  O -0.595248 4.517081 3.725681  C -1.704145 5.242762 4.191397  H -2.356396 5.567001 3.361227  H -2.303441 4.654417 4.908676  H -1.313289 6.131665 4.702106  C 5.904965 0.234531 0.212965  H 6.076244 -0.710744 0.757839  H 5.717307 0.004435 -0.850796  H 6.806992 0.854773 0.287391  H 2.855732 2.217035 1.717542  H 1.317113 3.106749 2.848490  C -2.878132 -1.006358 0.524817  H -3.265613 -2.015623 0.285805  H -3.454165 -0.675777 1.417164  C -3.153859 -0.113706 -0.662703  C -2.591889 -0.517003 -2.039744  C -4.555737 -0.084684 -1.326487  H -2.851650 0.936037 -0.465561  C -3.861713 0.053394 -2.689498  H -2.562258 -1.618827 -2.122722  H -1.606379 -0.123427 -2.342263  H -5.040179 -1.072482 -1.222064  H -5.277845 0.690062 -1.015552  H -4.293575 -0.475206 -3.555277  H -3.729722 1.113682 -2.966983  C 0.652269 -3.919493 1.952559  C -1.007513 -2.867302 0.671179  C -1.682550 -3.563938 1.850763  C -0.541797 -3.627478 2.855764  H 0.897581 -5.000252 1.932876  H 1.570199 -3.388990 2.286791  H -1.468899 -3.123598 -0.298418  H -2.569581 -3.041282 2.236910  H -1.987417 -4.594592 1.575501  H -0.424071 -2.645018 3.347650  H -0.675122 -4.385568 3.644233  O 0.287306 -3.511341 0.655492  **[F-G]^S^**  *E*_e_ : -2660.11375320  Ni -0.276647 -1.229237 1.044475  C 0.961558 1.328998 1.814047  C -1.072515 1.186187 2.881793  C -0.935038 2.447469 3.453246  C 0.228379 3.174099 3.173360  C 1.189347 2.597438 2.339026  C 1.914323 0.641379 0.919392  C 2.324626 -1.223818 -0.368731  C 3.582602 -0.771841 -0.744281  C 4.013619 0.467117 -0.253772  C 3.154518 1.175408 0.587751  H -1.964043 0.573447 3.064650  H -1.726791 2.836716 4.095939  H 1.952887 -2.182264 -0.747835  H 4.194832 -1.384406 -1.408430  N -0.160535 0.634800 2.084648  N 1.495049 -0.556735 0.441659  O 5.198298 1.034613 -0.531340  O 0.499943 4.400470 3.647234  C -0.434996 5.025089 4.483832  H -1.405449 5.173361 3.976452  H -0.604821 4.449678 5.411896  H -0.023230 6.007291 4.749563  C 6.092744 0.354886 -1.368796  H 6.389620 -0.622059 -0.945738  H 5.668285 0.190440 -2.375944  H 6.987453 0.983708 -1.462842  H 3.489859 2.140973 0.972355  H 2.092723 3.171484 2.121108  C -1.660814 -1.807740 -0.284188  H -0.940842 -1.177930 -0.866372  H -1.616095 -2.815302 -0.732541  C -3.020514 -1.174351 -0.463736  C -3.191974 0.317620 -0.132511  C -3.505906 -0.849023 -1.895387  H -3.814716 -1.748953 0.053183  C -4.124818 0.445915 -1.346878  H -2.235825 0.849412 -0.294101  H -3.569550 0.584179 0.867762  H -2.643452 -0.634157 -2.552287  H -4.159608 -1.573642 -2.409635  H -4.061701 1.356985 -1.964212  H -5.180921 0.298512 -1.062937  C -3.609798 -2.423572 2.961855  C -1.702739 -2.279206 1.615052  C -2.276783 -3.689312 1.522436  C -3.011489 -3.815223 2.847221  H -3.794692 -2.105031 4.002495  H -4.576057 -2.359502 2.418381  H -0.706869 -2.401079 2.234596  H -3.007887 -3.780566 0.696740  H -1.486302 -4.437361 1.356251  H -3.767674 -4.615148 2.870430  H -2.298360 -3.994442 3.671277  O -2.661675 -1.553662 2.369545  **G^S^**  *E*_e_ : -2660.15685612  Ni -1.108577 -0.572080 2.522632  C 0.052345 1.890054 2.291441  C -1.914464 2.065338 3.507526  C -1.761476 3.431388 3.676976  C -0.651928 4.058217 3.077102  C 0.262543 3.266904 2.391263  C 0.971721 0.966902 1.644992  C 1.664531 -1.229375 1.465105  C 2.543865 -0.940025 0.434196  C 2.629790 0.393169 -0.012660  C 1.824248 1.344561 0.606404  H -2.785876 1.553011 3.928661  H -2.512511 3.985655 4.242188  H 1.605001 -2.259880 1.843748  H 3.165335 -1.735361 0.016991  N -1.071318 1.295629 2.803716  N 0.877738 -0.332297 2.081795  O 3.426126 0.816710 -1.017082  O -0.394690 5.378944 3.142842  C -1.269234 6.202347 3.859268  H -2.288958 6.195259 3.431786  H -1.332513 5.911648 4.924279  H -0.873056 7.224718 3.798994  C 4.225452 -0.118312 -1.678897  H 4.960965 -0.591286 -1.001376  H 3.623635 -0.916177 -2.153516  H 4.772864 0.420083 -2.464230  H 1.849084 2.373408 0.235956  H 1.161060 3.734422 1.980414  C -2.503530 -2.449325 -0.118829  H -2.494970 -3.018187 -1.071080  H -3.463543 -2.680864 0.381767  C -2.401798 -0.972187 -0.420796  C -1.211373 -0.453829 -1.248290  C -3.338820 -0.347284 -1.467193  H -2.451448 -0.400599 0.531033  C -2.171645 0.555098 -1.897607  H -0.872946 -1.217100 -1.975779  H -0.329858 -0.066608 -0.708492  H -3.589327 -1.081024 -2.256318  H -4.274620 0.123095 -1.123067  H -2.050804 0.782378 -2.968792  H -2.175657 1.506478 -1.339476  C -1.030548 -3.434491 3.036042  C -1.382601 -2.995291 0.733247  C -1.406842 -4.495727 0.953638  C -0.597085 -4.649055 2.230325  H -0.224688 -2.999468 3.652512  H -1.886252 -3.652021 3.700637  H -0.402573 -2.691530 0.297660  H -2.451032 -4.826866 1.105597  H -0.997290 -5.051780 0.096430  H -0.773834 -5.595712 2.762226  H 0.481490 -4.594625 2.000383  O -1.450558 -2.464938 2.070860  **H^D^**  *E*_e_ : -5002.35174437  Ni -0.583257 -0.337293 0.657008  C 1.211515 1.851450 0.271638  C -0.924784 2.672137 0.609616  C -0.507541 3.989312 0.504456  C 0.851147 4.235664 0.258238  C 1.714502 3.132563 0.145510  C 2.061878 0.641634 0.220048  C 2.089189 -1.664524 0.429596  C 3.464986 -1.725102 0.270070  C 4.162969 -0.525017 0.069558  C 3.433309 0.677244 0.052514  H -1.979809 2.455115 0.807249  H -1.234955 4.793878 0.619699  H 1.509359 -2.576253 0.610701  H 3.968275 -2.691829 0.312390  N -0.107908 1.624703 0.488474  N 1.400324 -0.523937 0.390530  Br -0.870445 -1.441195 2.618959  O 5.468994 -0.424303 -0.097867  O 1.396443 5.430217 0.128997  C 0.594267 6.590357 0.249343  H -0.189963 6.612666 -0.525561  H 0.132307 6.650959 1.248711  H 1.261305 7.448422 0.109678  C 6.275110 -1.587316 -0.075244  H 6.204254 -2.100428 0.898209  H 5.992286 -2.282477 -0.883217  H 7.307836 -1.256056 -0.231974  H 3.984273 1.610037 -0.081506  H 2.774470 3.325610 -0.029752  C -2.499344 -0.305884 0.374371  H -2.983759 -1.226862 0.719801  H -2.925732 0.590865 0.845443  C -2.118748 -0.242055 -1.055499  C -2.717024 -1.226302 -2.088914  C -2.522114 0.939626 -1.966570  H -0.975277 -0.338577 -1.172836  C -2.731595 -0.073202 -3.105897  H -3.728297 -1.535554 -1.781066  H -2.138264 -2.129231 -2.332283  H -3.465990 1.392179 -1.620459  H -1.785135 1.743336 -2.121754  H -3.640843 0.043328 -3.712082  H -1.866523 -0.111007 -3.786613  **[HOMO-I]^D^**  *E*_e_ : -5234.67987864  Ni -2.630728 -3.305745 -1.502455  C -4.672806 -1.286395 -1.045654  C -4.750361 -1.881163 -3.272077  C -5.772056 -0.986451 -3.550041  C -6.258589 -0.195477 -2.500063  C -5.688297 -0.363836 -1.228901  C -4.046041 -1.532216 0.271207  C -2.470632 -2.738184 1.450281  C -2.824749 -2.146974 2.653363  C -3.846798 -1.190439 2.648541  C -4.463267 -0.890683 1.423268  H -4.355839 -2.505584 -4.080592  H -6.167834 -0.919800 -4.564341  H -1.677858 -3.492398 1.419686  H -2.303919 -2.440695 3.565620  N -4.199898 -2.037325 -2.067551  N -3.046232 -2.443083 0.282824  O -4.291186 -0.540218 3.710972  O -7.221565 0.703196 -2.605630  C -7.844816 0.923119 -3.855928  H -7.117507 1.274239 -4.606978  H -8.341073 0.007880 -4.219720  H -8.600172 1.701250 -3.697968  C -3.719764 -0.796346 4.978580  H -3.863191 -1.849012 5.274699  H -2.643992 -0.553357 4.987071  H -4.239055 -0.148917 5.694345  H -5.269700 -0.155723 1.424829  H -6.073168 0.249500 -0.412620  C -2.069961 -3.742112 -3.305739  H -1.456144 -4.652349 -3.349268  H -2.927188 -3.802886 -3.993176  C -1.313298 -2.461574 -3.272990  C 0.199525 -2.412523 -3.580250  C -1.539191 -1.340869 -4.312953  H -1.431467 -1.961176 -2.249337  C -0.030427 -1.047872 -4.251144  H 0.469669 -3.197807 -4.304875  H 0.894533 -2.472198 -2.729545  H -1.842641 -1.769190 -5.283405  H -2.237988 -0.528699 -4.054515  H 0.483473 -0.862374 -5.205106  H 0.195888 -0.212083 -3.569928  C -3.620123 -6.734736 0.340199  C -3.659227 -5.151191 -1.325294  C -3.347694 -6.495911 -1.987631  C -3.860962 -7.492157 -0.949766  H -2.565840 -6.812935 0.672323  H -4.268256 -7.033820 1.176797  H -2.112422 -4.848618 -1.139039  H -3.832319 -6.583488 -2.972055  H -2.264651 -6.643158 -2.147843  H -4.939911 -7.676268 -1.083580  H -3.344980 -8.462139 -0.988041  O -3.928056 -5.379928 0.013948  Br -0.520580 -4.730010 -0.587550  H -4.504193 -4.619289 -1.795035  **I^D^**  *E*_e_ : -2660.03056743  Ni -1.136290 -1.232559 0.518852  C 0.050333 1.094705 1.755106  C -2.195647 0.989129 2.289809  C -2.142404 2.159816 3.031990  C -0.912063 2.826769 3.125363  C 0.199133 2.269437 2.469201  C 1.167734 0.424262 1.048645  C 1.779035 -1.397792 -0.236455  C 3.102538 -0.988426 -0.303328  C 3.461275 0.199632 0.350516  C 2.463122 0.908703 1.041244  H -3.141141 0.442960 2.191851  H -3.045624 2.532016 3.517288  H 1.463774 -2.331190 -0.716129  H 3.825577 -1.592032 -0.853504  N -1.142393 0.462934 1.666012  N 0.832522 -0.719444 0.412623  O 4.678046 0.715313 0.377302  O -0.703816 3.949737 3.789958  C -1.773433 4.573609 4.473600  H -2.577070 4.862425 3.775785  H -2.182225 3.915280 5.258299  H -1.363441 5.476075 4.940998  C 5.734708 0.063178 -0.299967  H 5.908860 -0.945284 0.111129  H 5.530358 -0.007782 -1.381334  H 6.631600 0.673308 -0.143948  H 2.748710 1.829376 1.553688  H 1.155267 2.790627 2.546239  C -2.942913 -1.502291 -0.109862  H -2.905851 -2.371964 -0.794441  H -3.688261 -1.712665 0.676194  C -3.171686 -0.227243 -0.878472  C -2.076622 0.265553 -1.859802  C -4.148058 -0.213454 -2.082386  H -3.437693 0.605272 -0.199589  C -3.191554 0.751332 -2.797493  H -1.537832 -0.596296 -2.299103  H -1.329234 0.995630 -1.499376  H -4.163661 -1.202645 -2.572181  H -5.187904 0.100231 -1.901612  H -3.045446 0.635116 -3.881691  H -3.453212 1.803060 -2.596731  C 0.584780 -3.131586 2.733975  C -1.330934 -2.970366 1.444619  C -1.758706 -2.768372 2.879472  C -0.448258 -2.368561 3.550614  H 0.843069 -4.107089 3.182010  H 1.525123 -2.575072 2.575501  H -1.938395 -3.640006 0.819451  H -2.574069 -2.041837 3.007166  H -2.121488 -3.734857 3.278646  H -0.286149 -1.280079 3.469230  H -0.411501 -2.625866 4.618683  O -0.021046 -3.388610 1.463506 |
| --- |
